# GPhos Ligand Enables Production of Chiral *N*‐Arylamines in a Telescoped Transaminase‐Buchwald‐Hartwig Amination Cascade in the Presence of Excess Amine Donor

**DOI:** 10.1002/chem.202103472

**Published:** 2021-10-08

**Authors:** Christian M. Heckmann, Francesca Paradisi

**Affiliations:** ^1^ School of Chemistry University of Nottingham University Park Nottingham NG7 2RD UK; ^2^ Dept. of Chemistry Biochemistry and Pharmaceutical Sciences University of Bern Freiestrasse 3 3012 Bern Switzerland

**Keywords:** Biocatalysis, Buchwald-Hartwig amination, chemo-enzymatic synthesis, palladium, transaminases

## Abstract

The combination of biocatalysis and chemocatalysis can be more powerful than either technique alone. However, combining the two is challenging due to typically very different reaction conditions. Herein, chiral *N*‐aryl amines, key features of many active pharmaceutical ingredients, are accessed in excellent enantioselectivity (typically>99.5 % *ee*) by combining transaminases with the Buchwald‐Hartwig amination. By employing a bi‐phasic buffer‐toluene system as well as the ligand GPhos, the telescoped cascade proceeded with up to 89 % overall conversion in the presence of excess alanine. No coupling to alanine was observed.

## Graphical abstract

**Chiral** ***N*** **‐arylamines** were accessed by combining transaminases with the Buchwald‐Hartwig amination (BHA), combining the high enantioselectivity of the former with the efficient arylation ability of palladium. The ligand GPhos was key for the successful arylation of α‐secondary amines in a biphasic water‐toluene system in the presence of excess alanine, achieving overall conversions of up to 89 % with >99.5 % *ee*. No coupling to alanine was observed.

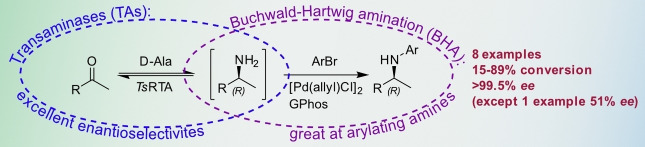

Biocatalysis is a powerful tool in the synthesis of fine chemicals;^[1]^ in particular, enzymes can achieve excellent enantioselectivities, which is highly relevant for the pharmaceutical industry.^[2]^ In synthesis routes, telescoping several reaction steps is often desirable as it reduces waste, material loss, and saves time by avoiding purification steps.^[3]^ However, combining chemical and enzymatic steps poses several key challenges, specifically in the compatibility of the reaction environments.^[4]^ Either the enzyme has to tolerate the conditions required by the chemical reaction (organic solvents, reagents such as heavy metals, high temperatures, high or low pH, etc.) or the chemical step has to be compatible with the environment required by the enzyme (aqueous medium, buffer salts, cofactors, mild temperature, mild pH, etc.). Additionally, biotransformations typically employ much more diluted conditions than chemical transformations. These challenges notwithstanding, there are several examples in the literature of such chemo‐enzymatic cascades which overcome some of these drawbacks by separating the reactions in time (e. g. one‐pot two‐step) or space (e. g. membrane reactors).[^4^, ^5^, ^6^, ^7^, ^8^]

Transaminases (TAs) are highly applicable to the synthesis of primary amines and can access either enantiomer depending on the enzyme used.^[9]^ However, active pharmaceutical ingredients (APIs) frequently contain secondary or tertiary amines, often with *N*‐aryl groups (Figure 1). While secondary and tertiary amines can be synthesised biocatalytically via the reductive amination of ketones with aliphatic amines using imine reductases (IREDs), this approach is more challenging when employing anilines and only a few examples with limited substrate scope have been reported.[^10^, ^11^] Chemo‐catalysed asymmetric reductive aminations often require undesirable reaction conditions, such as anhydrous organic solvents, high pressure, and high temperatures, and often result in lower enantiomeric excess than can be achieved biocatalytically.[^12^, ^13^, ^14^] Thus, an attractive strategy in the synthesis of chiral *N*‐arylamines is the combination of biocatalysis with subsequent arylation of the amine intermediate, employing the Pd‐catalysed Buchwald‐Hartwig amination (BHA). In 2020, the Turner group have reported for the first time the combination of amine dehydrogenases (AmDH) and IREDs with a BHA (Scheme 1A);^[15]^ however, they were unsuccessful in combining the BHA with TAs, as the presence of excess amine donor that is required for the TA step interfered with the BHA in their system. Compared to TAs, AmDHs typically have a narrower substrate scope^[16]^ and strong product inhibition^[17]^ and the IRED system could only be applied to preformed cyclic imines. Currently, the only example of combining TAs with Pd‐catalysis in a one‐pot system is from the Bornscheuer group who accessed α‐biarylamines by employing a Suzuki cross‐coupling followed by transamination (Scheme 1B).^[18]^ Herein, the TA‐BHA mediated synthesis of chiral *N*‐arylamines with excellent enantioselectivity and good to excellent conversions is reported, employing the crude biotransformation mixture in the BHA without the need for removal of excess alanine (Scheme 1C).

**Figure 1:**
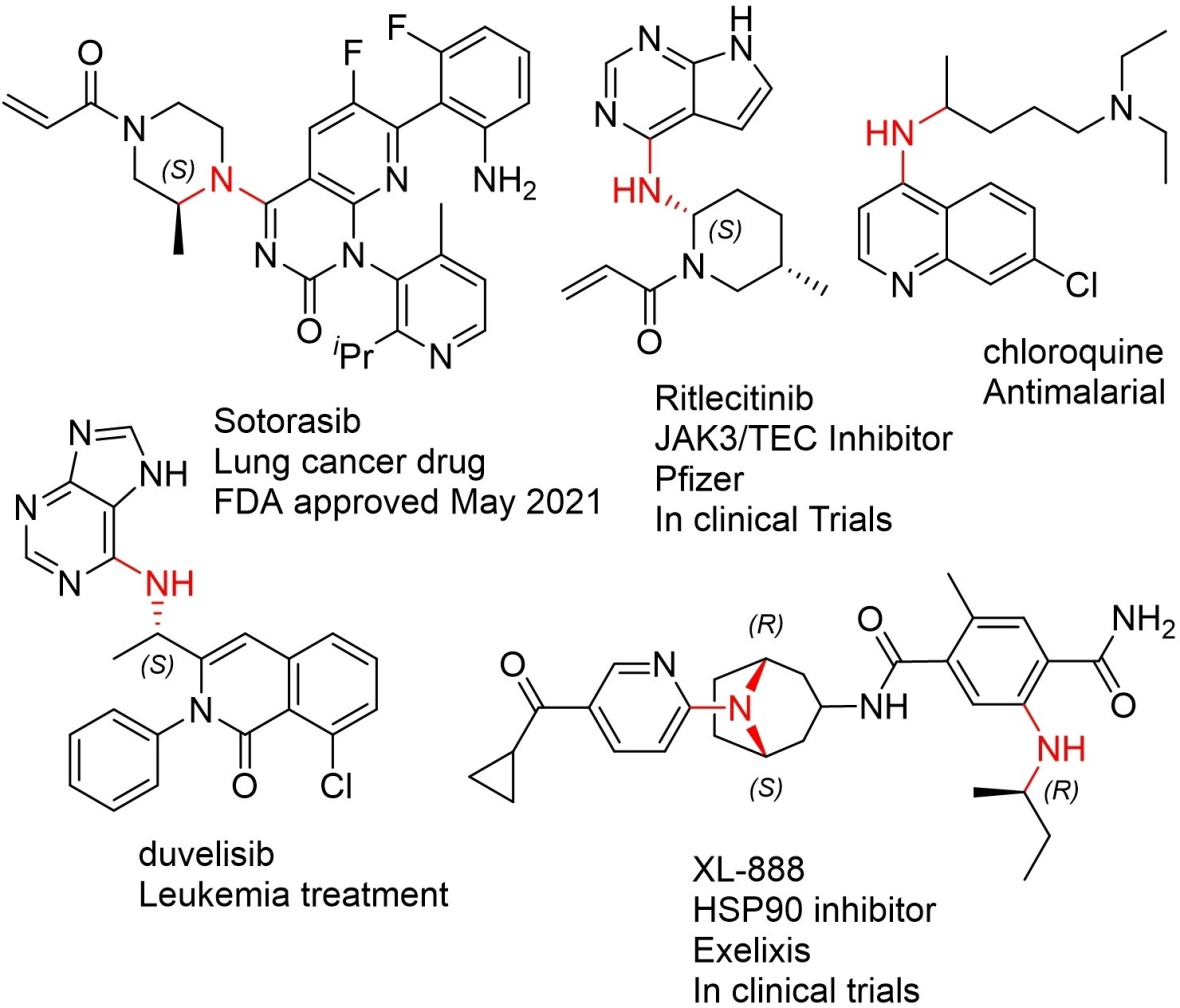
Selected structures of APIs containing chiral *N*‐arylamines.

**Scheme 1:**
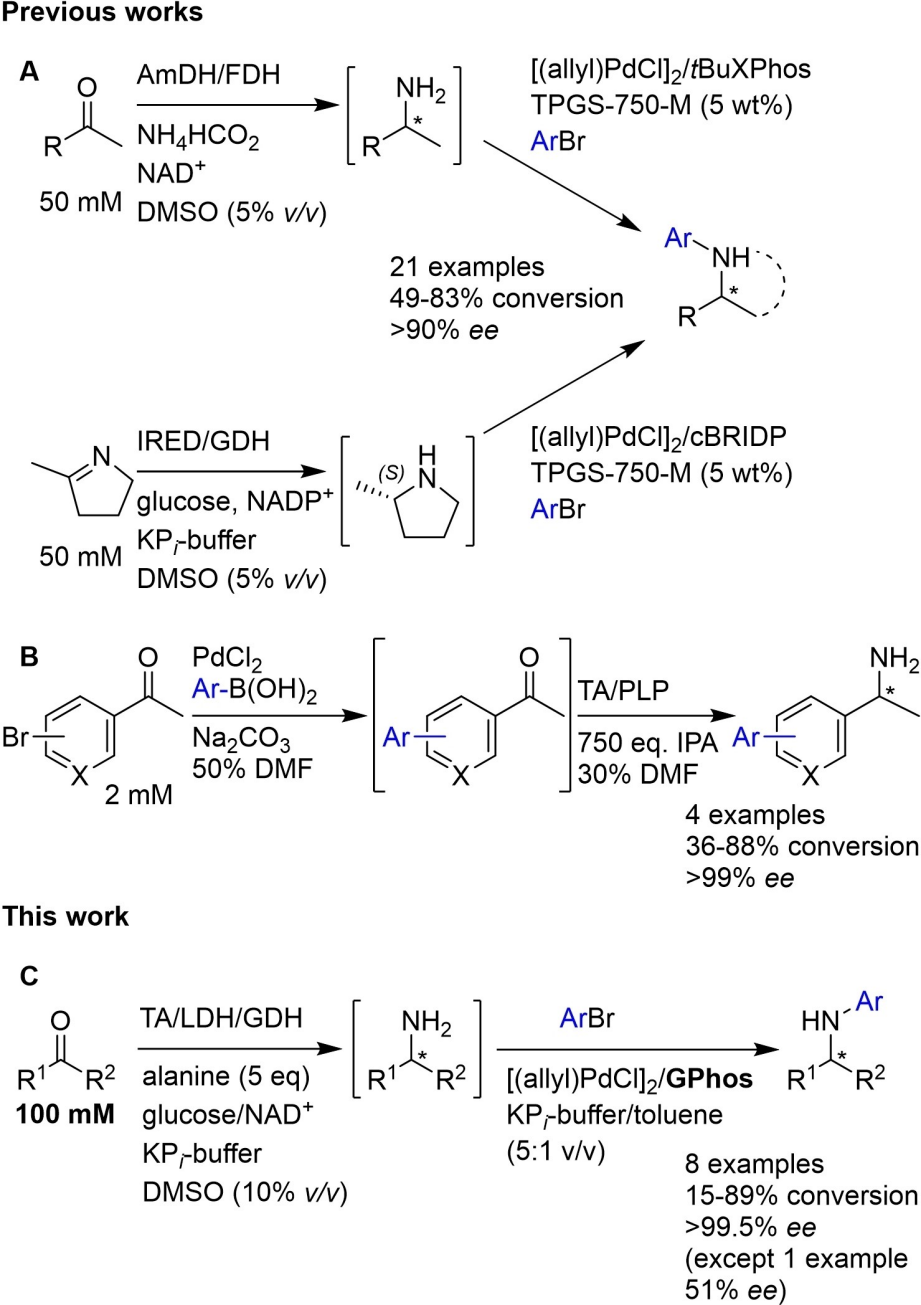
A: Chiral amines are produced from prochiral ketones using an AmDH (top) or a cyclic imine using an IRED (bottom), which are then arylated using a palladium catalyst to give chiral *N*‐arylamines.^[15]^ B: Suzuki cross‐coupling to produce bi‐aryl ketones which are then aminated using a transaminase biocatalyst.^[18]^ C: Chiral amines are produced from pro‐chiral ketones using a TA with alanine as the amine donor and arylated in the presence of excess alanine to give chiral *N*‐arylamines, enabled by the ligand GPhos. This reaction could be carried out using a higher substrate loading than in the previous examples.

Initially, two ligands, *t*BuXPhos and *t*BuBrettPhos, that had been reported to enable BHAs in water were investigated (Figure 2).^[19]^ For convenience, a biphasic system^[20]^ (employing the aqueous biotransformation and toluene) was chosen and the preformed Pd‐complex, a third‐generation Buchwald precatalyst, was added directly (Figure 2). Initial optimization was carried out using benzaldehyde as a model starting material, which is efficiently converted to benzylamine when using the (*R*)‐selective transaminase from *Thermomyces stellatus* (*Ts*RTA)^[21]^ with d‐alanine (5 equiv.) as the donor and employing an LDH/GDH pyruvate removal system to shift the equilibrium (Scheme 2). The results of the optimization are shown in Table 1. Crucially, the BHA required additional equivalents of base compared to standard conditions (entries 3–6), as some of the base is consumed in an acid‐base reaction with the protonated amines present in the biotransformation mixture (at pH 8). Sodium *t*‐butoxide was chosen based on Wagner et al.^[19]^ While essentially complete conversion to the *N*‐arylamine was achieved when using 10 mol% of *t*BuBrettphos‐Pd‐G3 (entry 3), using 5 mol% of the cheaper *t*BuXPhos‐Pd‐G3 still reached almost 80 % conversion (entry 7).

**Figure 2:**
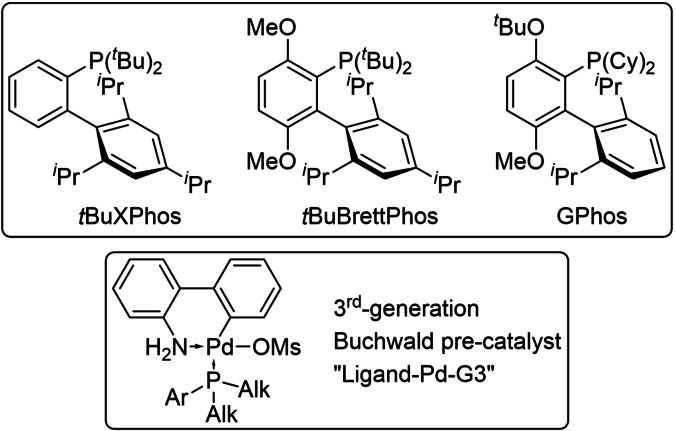
Structures of the ligands used (top) and 3^rd^‐generation Buchwald pre‐catalyst (bottom).

**Scheme 2:**
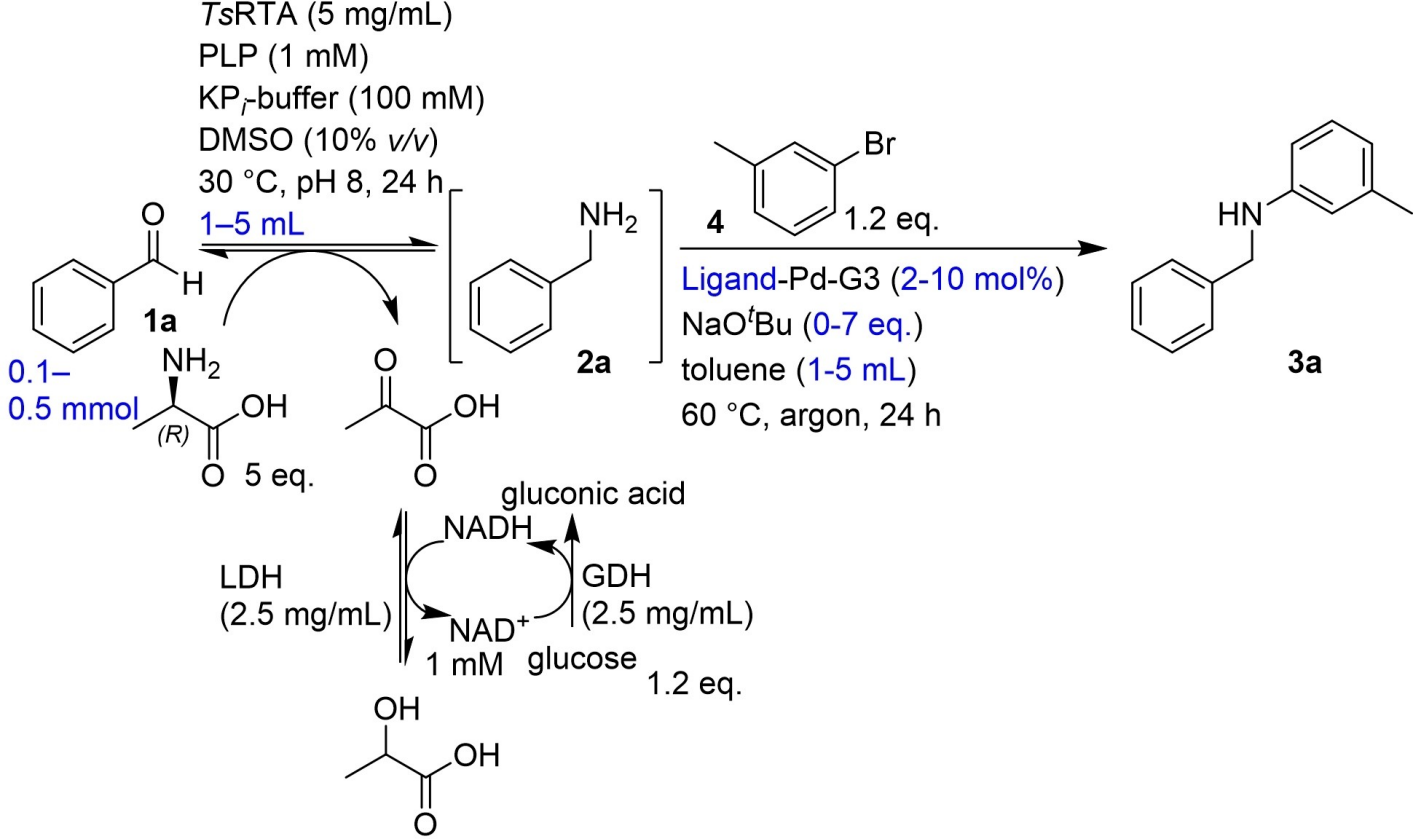
Model system used for the optimization of the BHA, showing all the components and by‐products from the transaminase‐catalysed biotransformation. Conditions optimized are shown in blue.

**Table 1:** Optimization of the BHA step, coming from a biotransformation of benzaldehyde with *Ts*RTA (Scheme 2).

Entry	Ligand	Catalyst loading [mol %]	Amount of 1 a [mmol]	Aqueous/toluene [*v/v*]	NaO*t*Bu [equiv.]	Conversion [%]^[a]^
1	*t*BuBrettPhos	2	0.1	1 : 1	7	28 (28)
2	*t*BuBrettPhos	5	0.1	1 : 1	7	81–92 (81–92)^[b]^
3	*t*BuBrettPhos	10	0.1	1 : 1	7	99 (97)
4	*t*BuBrettPhos	5	0.1	1 : 1	0	0 (0)
5	*t*BuBrettPhos	5	0.1	1 : 1	2	14 (14)
6	*t*BuBrettPhos	5	0.1	1 : 1	5	46 (45)
7	*t*BuXPhos	5	0.1	1 : 1	7	72–77 (71‐77)^[b]^
8	*t*BuXPhos	5	0.5	5 : 5	7	46–62 (45–62)^[b]^
9	*t*BuXPhos	5	0.5	5 : 1	7	75–83 (73–82)^[b]^
10	*t*BuXPhos	5	0.5	5 : 1^[c]^	7	93–94 (93–93)^[b]^

[a] Conversion of the BHA step followed by overall conversion in parentheses. Conversions determined by RP‐HPLC, comparing peak areas of benzaldehyde, benzylamine, and 3‐benzylaminotoluene, corrected for their response factor, after the BHA step. [b] Range observed across two independent experiments. [c] Precipitated protein was removed by centrifugation after biotransformation.

To improve the synthetic potential, reactions were scaled up to a 0.5 mmol scale, by increasing the volume of the biotransformation 5‐fold. Conversions dropped compared to the 0.1 mmol scale but could be improved by decreasing the ratio of toluene to the aqueous biotransformation (1 : 5 *v/v*) (Table 1 entries 8 and 9). Conversions were approximately 10 % higher when precipitated protein was removed from the biotransformation by centrifugation prior to the BHA (Table 1 entry 10). These conditions were then taken forward for the synthesis of chiral *N*‐arylamines. At no point coupling to alanine was observed. Alanine, presumably, exclusively acts as a poison of the catalyst rather than as a substrate.[^15^, ^22^] Partitioning of the alanine to the aqueous phase and the catalyst to the organic phase sufficiently slows this inactivation, allowing the reaction to occur (using the surfactant TPGS‐750‐M in preliminary experiments showed lower conversions in the presence of one equivalent of alanine when compared to the bi‐phasic system; data not shown).

Moving from benzaldehyde to the structurally similar *o*‐fluoroacetophenone (**1 b**) conversions in the BHA step were reduced significantly (Scheme 3). This is due to the increased steric hinderance of the secondary α‐carbon, which was confirmed indirectly when biotransformations employing the amine donor isopropylamine (IPA) were investigated. Here, selective arylation of benzylamine in the presence of excess IPA was observed, consistent with a faster reaction of the α‐primary amine. However, this selectivity disappeared when moving to the α‐secondary **2 b** (see Scheme S1). Pd‐black formation was observed in all reactions containing alanine (as well as in the reactions with benzaldehyde), indicating a lack of stability of the catalyst under these conditions. This Pd‐black is expected to be catalytically inactive.^[23]^

**Scheme 3:**
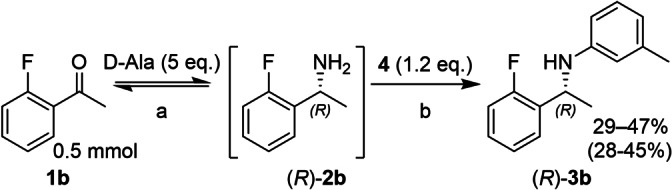
Telescoped TA‐BHA reaction starting from o‐fluoroacetophenone. a: d‐Glc (1.2 equiv.), NAD^+^ (1 mM), LDH (2.5 mg/mL), GDH (2.5 mg/mL), *Ts*RTA (5 mg/mL), PLP (1 mM), KP_*i*_ (100 mM), DMSO (10 % v/v), pH 8, 30 °C, 24 h; 5 mL. b: *t*BuXPhos‐Pd‐G3 (5 mol%), NaO*t*Bu (7 equiv.), toluene (1 mL), 60 °C, argon, 24 h. Conversion of the BHA step followed by overall conversion in parentheses. Conversions determined by RP‐HPLC, comparing peak areas of ketone, primary amine, and N‐arylamine, corrected for their response factor, after the BHA step. Range observed across two independent experiments. Precipitated protein was removed by centrifugation after biotransformation.

At this point in the investigation the Buchwald group reported a new ligand, GPhos, which is derived from BrettPhos (Figure 2).^[24]^ Crucially, it lacks the 4′‐*i*Pr group which enhances the amine substrate scope to α‐tertiary amines (in organic solvent). Given the similarity to *t*BuBrettPhos, it was decided to investigate whether GPhos could overcome the decreased performance with α‐secondary amines in the current aqueous system as well, by accelerating the coupling reaction relative to the catalyst inactivation in the presence of alanine. Pleasingly, conversions improved to 74 % with the new ligand (Scheme 4). Good conversions were also obtained starting from the aliphatic ketones phenoxy‐acetone (**1 c**) and hexan‐2‐one (**1 d**), while 2‐acetylthiazol (**1 e**) resulted in low conversions in the BHA. Starting from all four pro‐chiral ketones, *Ts*RTA exclusively produced the (*R*)‐enantiomer of the corresponding amine. This stereochemistry was retained after the BHA, except for **3 e**, which showed reduced *ee* of 51 %, indicating partial racemisation of the stereocentre, presumably by the Pd‐catalyst.^[25]^ Varying the aryl bromides employed in the BHA (Scheme 5), the *N*‐heterocycles pyridine (**5 a**) and isoquinoline (**5 b**) (common features in APIs)^[26]^ showed good conversions. Conversions improved with the electron‐poor benzonitrile (**5 c**), while the electron rich anisole (**5 d**) showed low conversions, complementing IRED catalysed reductive aminations with anilines which so far do not accept electron withdrawing substituents or pyridines.^[11]^

**Scheme 4:**
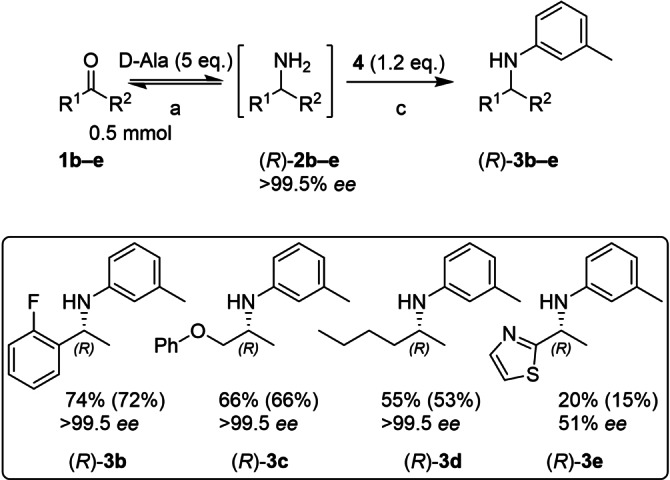
Ketone substrate scope of the telescoped TA‐BHA cascade, employing the ligand GPhos. a: d‐Glc (1.2 equiv.), NAD^+^ (1 mM), LDH (2.5 mg/mL), GDH (2.5 mg/mL), *Ts*RTA (5 mg/mL), PLP (1 mM), KP_*i*_ (100 mM), DMSO (10 % v/v), pH 8, 30 °C, 24 h; 5 mL. c: [Pd(allyl)Cl]_2_ (5 mol%), GPhos (6 mol%), NaO*t*Bu (7 equiv.), toluene (1 mL), 60 °C, argon, 24 h. Conversion of the BHA step followed by overall conversion in parentheses. Conversions determined by RP‐HPLC (3d: GC‐FID), comparing peak areas of ketone, primary amine, and N‐arylamine, corrected for their response factor, after the BHA step. Precipitated protein was removed by centrifugation after biotransformation. Enantiomeric excess was determined by chiral GC‐FID or RP‐HPLC, see Supporting Information.

**Scheme 5:**
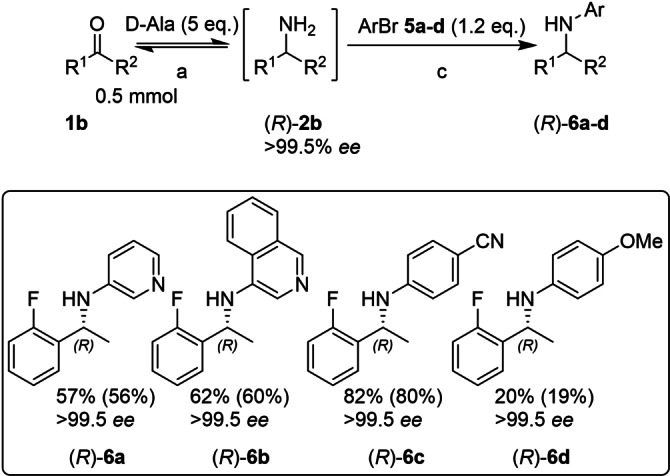
Ketone substrate scope of the telescoped TA‐BHA cascade, employing the ligand GPhos. a: D‐Glc (1.2 equiv.), NAD^+^ (1 mM), LDH (2.5 mg/mL), GDH (2.5 mg/mL), *Ts*RTA (5 mg/mL), PLP (1 mM), KP_*i*_ (100 mM), DMSO (10 % v/v), pH 8, 30 °C, 24 h; 5 mL. c: [Pd(allyl)Cl]_2_ (5 mol%), GPhos (6 mol%), NaO*t*Bu (7 equiv.), toluene (1 mL), 60 °C, argon, 24 h. Conversion of the BHA step followed by overall conversion in parentheses. Conversions determined by RP‐HPLC, comparing peak areas of ketone, primary amine, and N‐arylamine, corrected for their response factor, after the BHA step. Precipitated protein was removed by centrifugation after biotransformation. Enantiomeric excess was determined by chiral RP‐HPLC, see Supporting Information.

Finally, the catalyst loading was increased to 10 mol% for the reaction starting from **2 b** (Scheme 6), which increased the overall conversion to 89 %, with the optical purity fully maintained. In contrast, under otherwise identical conditions, *t*BuXPhos and *t*BuBrettPhos only reached 45 and 54 % overall conversion, respectively. Product **3 b** was isolated in 70 % isolated yield following silica column chromatography.

**Scheme 6:**
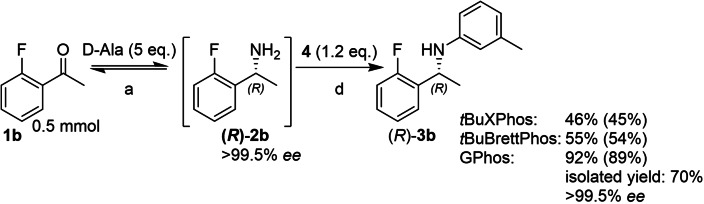
Increasing the catalyst loading in the BHA in the telescoped TA‐BHA cascade. a: d‐Ala (5 equiv.), d‐Glc (1.2 equiv.), NAD^+^ (1 mM), LDH (2.5 mg/mL), GDH (2.5 mg/mL), *Ts*RTA (5 mg/mL), PLP (1 mM), KP_*i*_ (100 mM), DMSO (10 % v/v), pH 8, 30 °C, 24 h; 5 mL. d: [Pd(allyl)Cl]_2_ (10 mol%), Ligand (12 mol%), NaO*t*Bu (7 equiv.), toluene (1 mL), 60 °C, argon, 24 h. Conversion of the BHA step followed by overall conversion in parentheses. Conversions determined by RP‐HPLC, comparing peak areas of ketone, primary amine, and N‐arylamine, corrected for their response factor, after the BHA step. Precipitated protein was removed by centrifugation after biotransformation. Isolated yield following column chromatography on silica gel (hexane/ethyl acetate (100:0 to 98 : 2 *v/v*)). Enantiomeric excess was determined by chiral RP‐HPLC, see Supporting Information.

In conclusion, the synthesis of chiral *N*‐arylamines in a sequential transaminase‐BHA cascade has been described. By using the ligand GPhos, up to 89 % overall conversion can be achieved starting from diverse pro‐chiral ketones, without intermediate purification steps. The current system can tolerate a large excess of alanine, although the formation of palladium black, also in the case of GPhos, indicates that the stability of the Pd‐catalyst needs to be further improved; coupling to alanine is not observed. As it has been reported that *t*BuXphos and *t*BuBrettPhos show superior performance in water compared to their cyclohexyl substituted analogues,^[19]^ the ^*t*^Bu‐substituted analogue of GPhos might further improve the current system. The use of different bases as well as co‐solvents^[27]^ could also be explored.

While a one‐pot two‐step reaction was possible, removing the precipitated protein from the biotransformation in a quick centrifugation improved conversion. The removal of the soluble enzymes may further improve the results; however, doing so by for example ultrafiltration^[8]^ would further complicate the reaction set‐up, and eliminate its main advantage which is the minimal processing that is required prior to the BHA step.

## Conflict of interest

The authors declare no conflict of interest.

## Supporting information

As a service to our authors and readers, this journal provides supporting information supplied by the authors. Such materials are peer reviewed and may be re‐organized for online delivery, but are not copy‐edited or typeset. Technical support issues arising from supporting information (other than missing files) should be addressed to the authors.

Supporting Information

## References

[1] R. A. Sheldon , D. Brady , M. L. Bode , Chem. Sci. 2020, 11, 2587–2605. 32206264 10.1039/c9sc05746c PMC7069372

[2] A. Fryszkowska , P. N. Devine , Curr. Opin. Chem. Biol. 2020, 55, 151–160. 32169795 10.1016/j.cbpa.2020.01.012

[3] P. N. Devine , R. M. Howard , R. Kumar , M. P. Thompson , M. D. Truppo , N. J. Turner , Nat. Chem. Rev. 2018, 2, 409–421.

[4] F. Rudroff , M. D. Mihovilovic , H. Gröger , R. Snajdrova , H. Iding , U. T. Bornscheuer , Nat. Catal. 2018, 1, 12–22.

[5] E. Burda , W. Hummel , H. Gröger , Angew. Chem. Int. Ed. 2008, 47, 9551–9554; 10.1002/anie.200801341 18942686

[6] S. Borchert , E. Burda , J. Schatz , W. Hummel , H. Gröger , J. Mol. Catal. B 2012, 84, 89–93.

[7] M. Cortes-Clerget , N. Akporji , J. Zhou , F. Gao , P. Guo , M. Parmentier , F. Gallou , J. Y. Berthon , B. H. Lipshutz , Nat. Commun. 2019, 10, 1–10. 31092815 10.1038/s41467-019-09751-4 PMC6520378

[8] J. Latham , J. M. Henry , H. H. Sharif , B. R. K. Menon , S. A. Shepherd , M. F. Greaney , J. Micklefield , Nat. Commun. 2016, 7, 1–8. 10.1038/ncomms11873 PMC4906404 27283121

[9] I. Slabu , J. L. Galman , R. C. Lloyd , N. J. Turner , ACS Catal. 2017, 7, 8263–8284.

[10] S. P. France , R. M. Howard , J. Steflik , N. J. Weise , J. Mangas-Sanchez , S. L. Montgomery , R. Crook , R. Kumar , N. J. Turner , ChemCatChem 2018, 10, 510–514.

[11] G. D. Roiban , M. Kern , Z. Liu , J. Hyslop , P. L. Tey , M. S. Levine , L. S. Jordan , K. K. Brown , T. Hadi , L. A. F. Ihnken , M. J. B. Brown , ChemCatChem 2017, 9, 4475–4479.

[12] M. Imai , T. Watanabe , M. Hatta , S. C. Das , M. Ozawa , K. Shinya , G. Zhong , A. Hanson , H. Katsura , S. Watanabe , C. Li , E. Kawakami , S. Yamada , M. Kiso , Y. Suzuki , E. A. Maher , G. Neumann , Y. Kawaoka , Nature 2012, 486, 420–428. 22722205 10.1038/nature10831 PMC3388103

[13] V. Skrypai , S. E. Varjosaari , F. Azam , T. M. Gilbert , M. J. Adler , J. Org. Chem. 2019, 84, 5021–5026. 30973732 10.1021/acs.joc.8b03073

[14] A. V. Malkov , S. Stončius , P. Kočovský , Angew. Chem. Int. Ed. 2007, 46, 3722–3724; 10.1002/anie.200700165 17415728

[15] S. C. Cosgrove , M. P. Thompson , S. T. Ahmed , F. Parmeggiani , N. J. Turner , Angew. Chem. Int. Ed. 2020, 59, 18156–18160; 10.1002/anie.202006246 PMC7590080 32628797

[16] V. Tseliou , T. Knaus , M. F. Masman , M. L. Corrado , F. G. Mutti , Nat. Commun. 2019, 10, 3717. 31420547 10.1038/s41467-019-11509-x PMC6697735

[17] A. Pushpanath , E. Siirola , A. Bornadel , D. Woodlock , U. Schell , ACS Catal. 2017, 7, 3204–3209.

[18] A. W. H. Dawood , J. Bassut , R. O. M. A. de Souza , U. Bornscheuer , Chem. Eur. J. 2018, 24, 16009–16013. 30156347 10.1002/chem.201804366

[19] P. Wagner , M. Bollenbach , C. Doebelin , F. Bihel , J.-J. Bourguignon , C. Salomé , M. Schmitt , Green Chem. 2014, 16, 4170–4178.

[20] J. R. Naber , S. L. Buchwald , Angew. Chem. Int. Ed. 2010, 49, 9469–9474; 10.1002/anie.201004425 21038337

[21] C. M. Heckmann , L. J. Gourlay , B. Dominguez , F. Paradisi , Front. Bioeng. Biotechnol. 2020, 8, 707. 32793563 10.3389/fbioe.2020.00707 PMC7387707

[22] Y. J. G. Renault , R. Lynch , E. Marelli , S. V. Sharma , C. Pubill-Ulldemolins , J. A. Sharp , C. Cartmell , P. Cárdenas , R. J. M. Goss , Chem. Commun. 2019, 55, 13653–13656. 10.1039/c9cc02554e 31593201

[23] T. Noël , S. L. Buchwald , Chem. Soc. Rev. 2011, 40, 5010–5029. 21826351 10.1039/c1cs15075h

[24] S. D. McCann , E. C. Reichert , P. L. Arrechea , S. L. Buchwald , J. Am. Chem. Soc. 2020, 142, 15027–15037. 32786769 10.1021/jacs.0c06139 PMC8057821

[25] S. Wagaw , R. A. Rennels , S. L. Buchwald , J. Am. Chem. Soc. 1997, 119, 8451–8458.

[26] E. Vitaku , D. T. Smith , J. T. Njardarson , J. Med. Chem. 2014, 57, 10257–10274. 25255204 10.1021/jm501100b

[27] J. C. Yang , D. Niu , B. P. Karsten , F. Lima , S. L. Buchwald , Angew. Chem. Int. Ed. 2016, 55, 2531–2535; 10.1002/anie.201509922 26756279

