# Supplementary material for: GPhos Ligand Enables Production of Chiral N‐Arylamines in a Telescoped Transaminase‐Buchwald‐Hartwig Amination Cascade in the Presence of Excess Amine Donor
Source: Chemistry. 2021 Oct 8;27(67):16616–20. doi: 10.1002/chem.202103472 (PMC9292530; doi:10.1002/chem.202103472)
Supplement: Supplementary file 1 — Supporting Information [file CHEM-27-16616-s001.pdf]

Chemistry–A European Journal

Supporting Information

GPhos Ligand Enables Production of Chiral *N*-Arylamines in a Telescoped Transaminase-Buchwald-Hartwig Amination Cascade in the Presence of Excess Amine Donor

Christian M. Heckmann* and Francesca Paradisi*

1 Table of Contents

2 Supporting Figures 5

3 Materials and Methods 5

3.1 Materials 5

3.2 Expression of TsRTA and GDH 5

3.3 Expression of HEwT 6

3.4 Enzyme lyophilization 6

3.5 Activity assays 6

3.6 General method for the sequential cascade to produce N-aryl amines using GPhos 6

3.7 Synthesis of N-arylamine standards 7

3.7.1 3-benzylaminotoluene hydrochloride **3a** 7

3.7.2 Synthesis of all other N-arylamine standards 8

3.8 Analytical techniques 12

3.8.1 Reverse-phase HPLC analysis of conversions 12

3.8.2 Chiral GC-FID 12

3.8.3 Chiral RP-HPLC 13

4 Chromatograms of enantiomeric excess determination 14

4.1 Biotransformations 14

4.1.1 o-fluoro-α-methylbenzylamine **2b**, acetylated (GC-FID) 14

4.1.2 2-phenoxyisopropylamine **2c**, acetylated (GC-FID) 15

4.1.3 hexan-2-amine **2d**, acetylated (GC-FID) 15

4.1.4 1-(thiazol-2-yl)ethylamine **2e**, acetylated (GC-FID) 16

4.2 Combined Cascade 16

4.2.1 3-(hexan-2-amino)toluene **3d**, underivatized (GC-FID) 16

4.2.2 3-(o-F-methylbenzylamino)toluene **3b**, acetylated (RP-HPLC) 17

4.2.3 3-(2-phenoxyisopropylamino)toluene **3c**, acetylated (RP-HPLC) 17

4.2.4 3-(1-(thiazol-2-yl)ethylamino)toluene **3e**, underivatized (RP-HPLC) 18

4.2.5 3-(o-F-methylbenzylamino)pyridine **6a**, trifluoroacetylated (RP-HPLC) 18

4.2.6 4-(o-F-methylbenzylamino)isoquinoline **6b**, trifluoroacetylated (RP-HPLC) 19

4.2.7 4-(o-F-methylbenzylamino)benzonitrile **6c**, trifluoroacetylated (RP-HPLC) 19

4.2.8 4-(o-F-methylbenzylamino)anisole **6d**, acetylated (RP-HPLC) ... 20

5 NMR-spectra ... 21

5.1 (R)-3-(o-fluoro-α-methylbenzylamino)toluene (R)-**3b** ... 21

5.1.1 ^{1}H-NMR ... 21

5.1.2 ^{13}C-{^{1}H}-NMR ... 22

5.1.3 ^{19}F-NMR ... 23

5.1.4 ^{1}H-^{1}H-COSY ... 24

5.1.5 ^{1}H-^{13}C-HSQC-ME ... 25

5.1.6 ^{1}H-^{13}C-HMBC ... 26

5.2 3-benzylaminotoluene hydrochloride **3a** ... 27

5.2.1 ^{1}H-NMR ... 27

5.2.2 ^{13}C-{^{1}H}-NMR ... 28

5.2.3 ^{1}H-^{1}H-COSY ... 29

5.2.4 ^{1}H-^{13}C-HSQC-ME ... 30

5.2.5 ^{1}H-^{13}C-HMBC ... 31

5.3 3-isopropylaminotoluene ... 32

5.3.1 ^{1}H-NMR ... 32

5.3.2 ^{13}C-{^{1}H}-NMR ... 33

5.3.3 ^{1}H-^{1}H-COSY ... 34

5.3.4 ^{1}H-^{13}C-HSQC-ME ... 35

5.3.5 ^{1}H-^{13}C-HMBC ... 36

5.4 3-(o-fluoro-α-methylbenzylamino)toluene **3b** ... 37

5.4.1 ^{1}H-NMR ... 37

5.4.2 ^{13}C-{^{1}H}-NMR ... 38

5.4.3 ^{19}F-NMR ... 39

5.4.4 ^{1}H-^{1}H-COSY ... 40

5.4.5 ^{1}H-^{13}C-HSQC-ME ... 41

5.4.6 ^{1}H-^{13}C-HMBC ... 42

5.5 (S)-3-(2-phenoxyisopropylamino)toluene (S)-**3c** ... 43

5.5.1 ^{1}H-NMR ... 43

5.5.2 ^{13}C-{^{1}H}-NMR ... 44

5.5.3 ^{1}H-^{1}H-COSY ... 45

5.5.4 ^{1}H-^{13}C-HSQC-ME ... 46

5.5.5 ^{1}H-^{13}C-HMBC 47
5.6 3-(hexan-2-amino)toluene **3d** 48
5.6.1 ^{1}H-NMR 48
5.6.2 ^{13}C-{^{1}H}-NMR 49
5.6.3 ^{1}H-^{1}H-COSY 50
5.6.4 ^{1}H-^{13}C-HSQC-ME 51
5.6.5 ^{1}H-^{13}C-HMBC 52
5.7 3-(1-(thiazol-2-yl)ethylamino)toluene **3e** 53
5.7.1 ^{1}H-NMR 53
5.7.2 ^{13}C-{^{1}H}-NMR 54
5.7.3 ^{1}H-^{1}H-COSY 55
5.7.4 ^{1}H-^{13}C-HSQC-ME 56
5.7.5 ^{1}H-^{13}C-HMBC 57
5.8 3-(o-fluoro-α-methylbenzylamino)pyridine **6a** 58
5.8.1 ^{1}H-NMR 58
5.8.2 ^{13}C-{^{1}H}-NMR 59
5.8.3 ^{19}F-NMR 60
5.8.4 ^{1}H-^{1}H-COSY 61
5.8.5 ^{1}H-^{13}C-HSQC-ME 62
5.8.6 ^{1}H-^{13}C-HMBC 63
5.9 4-(o-fluoro-α-methylbenzylamino)isoquinoline **6b** 64
5.9.1 ^{1}H-NMR 64
5.9.2 ^{13}C-{^{1}H}-NMR 65
5.9.3 ^{19}F-NMR 66
5.9.4 ^{1}H-^{1}H-COSY 67
5.9.5 ^{1}H-^{13}C-HSQC-ME 68
5.9.6 ^{1}H-^{13}C-HMBC 69
5.10 4-(o-fluoro-α-methylbenzylamino)benzonitirile **6c** 70
5.10.1 ^{1}H-NMR 70
5.10.2 ^{13}C-{^{1}H}-NMR 71
5.10.3 ^{19}F-NMR 72
5.10.4 ^{1}H-^{1}H-COSY 73
5.10.5 ^{1}H-^{13}C-HSQC-ME 74

5.10.6 ^{1}H-^{13}C-HMBC 75

5.11 4-(o-fluoro-α-methylbenzylamino)anisole **6d** 76

5.11.1 ^{1}H-NMR 76

5.11.2 ^{13}C-{^{1}H}-NMR 77

5.11.3 ^{19}F-NMR 78

5.11.4 ^{1}H-^{1}H-COSY 79

5.11.5 ^{1}H-^{13}C-HSQC-ME 80

5.11.6 ^{1}H-^{13}C-HMBC 81

6 Supporting References 81

2 Supporting Figures

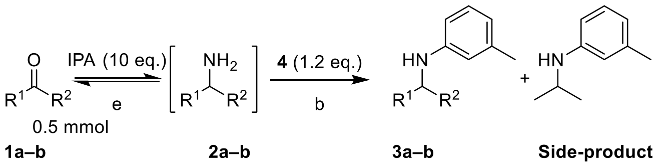

3a: 63–68% (63–68%); Ratio to side-product: 1.3–1.5
3b: 25–28% (22–25%); Ratio to side-product: 0.14–0.15
Expected ratio assuming no selectivity: 0.11

Scheme S1: Telescoped TA-BHA cascade with IPA as the amine donor. Unlike with alanine, coupling to the amine donor was observed as a side-product. e: HEwT (5 mg/mL), PLP (1 mM), KP_i (100 mM), DMSO (10% *v/v*), pH 8, 30 °C, 24 h; 5 mL. b: *t*BuXPhos-Pd-G3 (**3a**: 5 mol%, **3b**: 10 mol%), NaO^tBu (7 eq.), toluene (1 mL), 60 °C, argon, 24 h. Conversion of the BHA step followed by overall conversion in parentheses. Conversions determined by RP-HPLC, comparing peak areas of ketone, primary amine, and *N*-arylamine, corrected for their response factor, after the BHA step. Range observed across two independent experiments. Precipitated protein removed by centrifugation after biotransformation.

3 Materials and Methods

3.1 Materials

All cells used were propagated from lab stocks. Unstained Protein Standard, Broad Range from New England Biolabs® was the weight marker used for SDS–PAGE. Centrifugation: for small volumes the accuSpin™ Micro 17R centrifuge from Fisher Scientific™ and for large volumes the Heraeus™ Multifuge™ X3R from Thermo Scientific were used. Activity assays as well as DNA and protein quantification were carried out using the Epoch 2 Microplate Spectrophotometer from BioTek Instruments. Cells were lysed using a Fisherbrand™ Model 120 Sonic Dismembrator. Reagents were purchased from Arcos organics, Sigma Aldrich, Thermo Fisher, Alfa Aesar, Activate Scientific, Apollo Scientific, or Fluorochem and used without further purification. Restriction enzymes, polymerases and ligases were purchased from New England Biolabs. LDH-4 was provided by Johnson Matthey, and is commercially available. NMR spectra were obtained using a Bruker 400 MHz NMR spectrometer (Bruker AV3400 or AV3400HD) and referenced relative to the residual protonated solvent peak. ESI-MS data were obtained on a Bruker MicroTOF spectrometer.

3.2 Expression of TsRTA[1] and GDH[2]

The plasmid containing the gene was transformed into BL21 STAR (DE3) *E. coli* cells. 300 mL of TB-lac (amp 100 µg/mL or kan 50 µg/mL) were inoculated with a single colony and incubated at 37 °C, with shaking (180 rpm, 19 mm throw) for 4 h followed by 25 °C with shaking (180 rpm, 19 mm throw) for 20 h. Cells were harvested by centrifugation (4500 g, 15 min, 4 °C) and stored at -20 °C either as pellets or in resuspension buffer.

3.3 *Expression of HEwT*[3]

E. coli BL21 (DE3) or BL21 STAR™ (DE3) were transformed with pMP89a–HEwT and grown on selective LB–agar plates (amp 100 µg/mL) at 37 °C overnight. ZYP–AI medium[4] (300 mL; amp 100 µg/mL) was inoculated with a single colony of transformed *E. coli* and incubated for 20 h at 37 °C, 180 rpm (19 mm throw). Cells were harvested (4500 g, 20 min, 4 °C) and stored at –20 °C either as pellets or in loading buffer.

3.4 *Enzyme lyophilization*

Pellets were resuspended in buffer (potassium phosphate (50 mM), PLP (0.1 mM (HEwT), 1 mM (*Ts*RTA)), pH 8.0; 3:1 *v:w*), lysed on ice by sonication ($^1/_4$″ probe ($^1/_8$″ for <2 mL), 50% amplitude, 5 s on, 5 s off, for 12 min), and cell debris was removed by centrifugation (25,500 g, 4 °C, 60 min. The cfe was filtered (0.2 µm), frozen, and lyophilized to obtain a beige powder, which was stored at -20 °C.

3.5 *Activity assays*

Activity assays were based on the method by Schätzle *et al.*[5] as applied in Cerioli *et al.*[3], in UV-free 96-well plates using the EPOCH 2 plate reader at 30 °C, following the production of acetophenone from SMBA at 245 nm (pathlength 0.84 cm, calculated according to $\frac{A_{977}-A_{900}}{0.18}$); ε = 12.6 mM^{-1} cm^{-1}).

3.6 *General method for the sequential cascade to produce N-aryl amines using GPhos*

Biotransformations were set up in a final volume of 5 mL, containing the ketone (or aldehyde) substrate (0.5 mmol), PLP (1 mM), DMSO (10% *v/v*), and KP_i-buffer (100 mM), D-Ala (5 eq.), D-Glc (1.2 eq.), NAD^+ (1 mM), *Ts*RTA (lyo. cfe, 25 mg), GDH (lyo. cfe, 12.5 mg), and LDH-4 (lyo. cfe, 12.5 mg). Ketones were added from 10-fold concentrated stocks in DMSO, NAD^+ was added from a 100-fold concentrated stock in water. The other components were added from 10-fold pH adjusted stocks in buffer. Biotransformations were incubated at 30 °C with gentle shaking for 24h. Reaction mixtures were stored frozen at -20 °C or used directly in the BHA step.

Sodium *t*-butoxide (336 mg, 3.50 mmol, 7 eq.), $[Pd(allyl)Cl]_2$ (9.2 mg, 25 µmol, 5 mol%), and GPhos (16.1 mg, 30 µmol, 6 mol%) were placed in a flask which was flushed with argon, then the aryl halide (0.6 mmol, 1.2 eq.) was added while the flask was under argon. Next, toluene (1 mL) that had been thoroughly degassed (10 min sparging with argon under sonication) was added and the suspension sparged with argon for 5 min with vigorous stirring. Finally, the biotransformation mixture (which had been centrifuged (4800 g (4700 rpm), 5 min) to remove precipitated protein and degassed in the same way as the toluene) was added and the solution sparged for a further 2 min. Then, the solution was heated to 60 °C with vigorous stirring under argon (balloon) for 24 h. After cooling to RT,

samples of both the aqueous and organic phase were taken and analysed by RP-HPLC to assess conversions. Alternatively, reactions were extracted with ethyl acetate (3×10 mL) and the combined organic phases analysed by GC-FID (following acetylation); *ee*s were determined following extraction as above, either by chiral GC-FID or chiral RP-HPLC. See below for details on the chromatography and derivatization strategies.

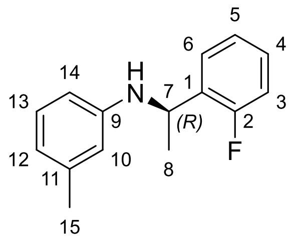

(*R*)-3-(o-fluoro-α-methylbenzylamino)toluene, starting from *o*-fluoroacetophenone and coupling with 3-bromotoluene was synthesised as above but using Pd(allyl)Cl]$_2$ (18.3 mg, 50 µmol, 10 mol%), and GPhos (32.2 mg, 60 µmol, 12 mol%). After the extraction into ethyl acetate, solvent were removed *in vacuo*, and the product purified by flash chromatography on silica gel using hexane/ethyl acetate (100:0 to 98:2 *v/v*), giving (*R*)-3-(*o*-fluoro-α-methylbenzylamino)toluene as a viscous bright-yellow oil (79.8 mg, 0.348 mmol, 70% yield). ^{1}H-NMR (400 MHz, CDCl$_3$) δ 1.53 (3 H, d (*J* 6.7 Hz), C8*H*$_3$), 2.22 (3 H, s, C15*H*$_3$), 4.81 (1 H, q (*J* 6.7 Hz), C7*H*), 6.33 (1 H, dd (*J* 8.1, 2.4 Hz), C14*H*), 6.38 (1 H, t (*J* 2.1 Hz), C10*H*), 6.49 (1 H, d (*J* 7.4 Hz), C12*H*), 6.99 (1 H, t (*J* 7.8 Hz), C13*H*), 7.01–7.09 (2 H, m, C3*H*+C5*H*), 7.19 (1 H, tdd (*J* 7.5, 5.3, 1.9 Hz), C4*H*), 7.38 (1 H, td (*J* 7.9, 1.9 Hz), C6*H*); ^{13}C-NMR (101 MHz, CDCl$_3$) δ 21.6 (*C*15), 23.3 (*C*8), 47.6 (d ($J_{C\text{-}F}$ 2.9 Hz), *C*7), 110.4 (*C*14), 114.2 (*C*10), 115.4 (d ($J_{C\text{-}F}$ 22.1 Hz), *C*3), 118.6 (*C*12), 124.4 (d ($J_{C\text{-}F}$ 3.5 Hz), *C*5), 127.2 (d ($J_{C\text{-}F}$ 4.5 Hz), *C*6), 128.3 (d ($J_{C\text{-}F}$ 8.2 Hz), *C*4), 129.0 (*C*13), 131.7 (d ($J_{C\text{-}F}$ 13.2 Hz), *C*1), 138.9 (*C*11), 146.7 (*C*9), 160.5 (d ($J_{C\text{-}F}$ 244.8 Hz), *C*2); ^{19}F-NMR (376 MHz, CDCl$_3$) δ -120.4 (ddd ($J_{F\text{-}H}$ 10.7, 7.7, 5.3 Hz), C2*F*). m/*z* [M+H]$^+$ calculated: 230.1340, found: 230.1345.

3.7 *Synthesis of N-arylamine standards*

3.7.1 *3-benzylaminotoluene hydrochloride **3a***

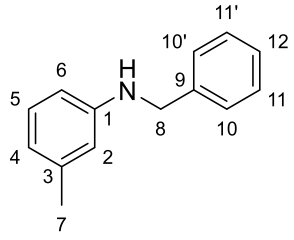

Benzylamine (98.3 µL, 96.4 mg, 0.9 mmol), 3-bromotoluene (121.2 µL, 171.03 mg, 1 mmol), [(cinnamyl)PdCl]$_2$ (5.7 mg, 11 µmol), 1.1 mol%), *t*BuXPhos (18.7, 44 µmol, 4.4 mol%), and TBAB (16.1 mg, 50 µmol, 5 mol%) were added to toluene (1 mL) and degassed by sparging with argon for 5 min. A degassed (15 min sparging with argon) aqueous solution of potassium hydroxide (2 M, 1 mL) was added and the reaction heated to 50 °C under vigorous stirring (1200 rpm) for 19 h. Aqueous sodium hydroxide (5M, 200 µL) was added and the reaction mixture extracted with ethyl acetate (3×3 mL). The combined organics were washed with brine (2 mL), dried over MgSO$_4$, and filtered through a silica plug (3 cm in Pasteur pipette). Methanolic HCl (approx. 3 M, prepared from methanol and acetyl chloride, 0.5 mL) was added and after cooling at -20 °C the resulting precipitate was obtained by vacuum filtration, washed with a mixture of ice cold hexane and

ethyl acetate (6:4 (*v:v*), 5 mL), giving 3-benzylaminotoluene hydrochloride as white fine needles (157.7 mg, 0.675 mmol, 75% yield). ^{1}H-NMR (400 MHz, $CDCl_3$) δ 2.26 (3 H, s, C7H_3), 4.32 (2 H, s, C8*H*), 7.08–7.19 (4 H, m, C2*H*+C4*H*+C5*H*+C6*H*), 7.21–7.26 (3 H, m, C11*H*+C11′*H*+C12*H*), 7.37 (2 H, dd (*J* 6.6, 2.9 Hz), C10*H*+C10′*H*), 11.69 (2 H, br s, NH_2); ^{13}C-NMR (101 MHz, $CDCl_3$) δ 21.2 (*C*7), 56.0 (*C*8), 120.9 (*C*6 or 5), 124.2 (*C*2 or 4), 128.6 (*C*11+*C*11′), 129.3 (*C*5, 6, or 12), 129.4 (*C*5, 6, or 12), 129.5 (*C*9), 130.0 (*C*2 or 4), 131.1 (*C*10+*C*10′), 134.2 (*C*1), 140.0 (*C*3). m/*z* $[M+H]^+$ calculated: 198.1277, found: 198.1279.

3.7.2 *Synthesis of all other N-arylamine standards*

Reactions containing amine (1.00 mmol), aryl halide (1.20 mmol), *t*BuXPhos-Pd-G3 (39.7 mg, 50.0 µmol, 5 mol%), and sodium *t*-butoxide (192.2 mg, 2.00 mmol) were set up as follows: solid reagents were placed in a flask which was flushed with argon, then liquid reagents were added while the flask was under argon. Next, toluene (1 mL) that had been thoroughly degassed (10 min sparging with argon under sonication) was added and the suspension sparged with argon for 2 min with vigorous stirring. Finally, aqueous buffer (1 mL; potassium phosphate (50 mM), DMSO 10 % (*v/v*), pH 8) was added and the solution sparged for a further 2 min. Then, the solution was heated to 60 °C with vigorous stirring under argon for 16-24 h. Reactions were allowed to cool and extracted with ethyl acetate (3×3 mL). The organic phases were combined, and solvents removed *in vacuo*. Compounds were then purified by flash chromatography on silica gel.

3.7.2.1 *3-isopropylaminotoluene*

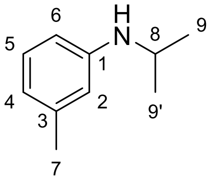

From isopropylamine (1 mL of a 1 M stock in the aqueous buffer) and 3-bromotoluene (145.6 µL, 205.2 mg, 1.20 mmol). Purified by flash chromatography on silica gel using hexane/ethyl acetate (10:0 to 9:1 *v/v*), giving 3-isopropylaminotoluene as a mandarin-coloured oil (117.0 mg, 0.784 mmol, 78% yield). ^{1}H-NMR (400 MHz, $CDCl_3$) δ 1.21 (6 H, d (*J* 6.3 Hz), C9H_3+C9′H_3), 2.28 (3 H, s, C7H_3), 3.63 (1 H, hept (*J* 6.3 Hz), C8*H*), 6.40–6.44 (2 H, m, C2*H*+6*H*), 6.51 (1 H, d (*J* 7.5 Hz), C4*H*), 7.06 (1 H, td (*J* 7.3, 1.5 Hz), C5*H*); ^{13}C-NMR (101 MHz, $CDCl_3$) δ 21.6 (*C*7), 23.0 (*C*9+*C*9′), 44.3 (*C*8), 110.5 (*C*6), 114.1 (*C*2), 118.0 (*C*4), 129.1 (*C*5), 139.0 (*C*3), 147.4 (*C*1). m/*z* $[M+H]^+$ calculated: 150.1277, found: 150.1277.

3.7.2.2 *3-(o-fluoro-α-methylbenzylamino)toluene* ***3b***

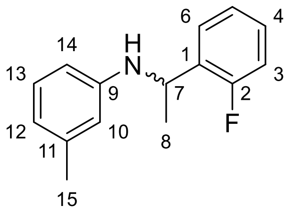

From *o*-fluoro-α-methylbenzylamine (130.2 µL, 1.00 mmol) and 3-bromotoluene (145.6 µL, 205.2 mg, 1.20 mmol), using *t*BuXPhos-Pd-G3 (31.5 mg, 40.0 µmol, 4 mol%). Purified by flash chromatography on silica gel using hexane/ethyl acetate (100:0 to 98:2 *v/v*), giving 3-(*o*-fluoro-α-methylbenzylamino)toluene as a pale-yellow oil (192.4 mg, 0.839 mmol, 84% yield). ^{1}H-NMR (400 MHz, $CDCl_3$) δ 1.53 (3 H, d (*J* 6.7 Hz), C8H_3), 2.22 (3 H, s, C15H_3), 4.08 (1 H, br

s, N*H*), 4.81 (1 H, q (*J* 6.7 Hz), C7*H*), 6.32 (1 H, dd (*J* 8.1, 2.4 Hz), C14*H*), 6.38 (1 H, t (*J* 2.0 Hz), C10*H*), 6.49 (1 H, d (*J* 7.4 Hz), C12*H*), 6.99 (1 H, t (*J* 7.7 Hz), C13*H*), 7.01–7.09 (2 H, m, C3*H*+C5*H*), 7.19 (1 H, tdd (*J* 7.5, 5.3, 1.8 Hz), C4*H*), 7.38 (1 H, td (*J* 7.8, 1.7 Hz), C6*H*); ^{13}C-NMR (101 MHz, CDCl$_3$) δ 21.6 (*C*15), 23.3 (*C*8), 47.5 (d (J_{C-F} 2.9 Hz), *C*7), 110.3 (*C*14), 114.2 (*C*10), 115.4 (d (J_{C-F} 21.7 Hz), *C*3), 118.6 (*C*12), 124.4 (d (J_{C-F} 3.4 Hz), *C*5), 127.2 (d (J_{C-F} 4.5 Hz), *C*6), 128.3 (d (J_{C-F} 8.1 Hz), *C*4), 129.0 (*C*13), 131.7 (d (J_{C-F} 13.2 Hz), *C*1), 138.9 (*C*11), 146.8 (*C*9), 160.5 (d (J_{C-F} 244.4 Hz), *C*2); ^{19}F-NMR (376 MHz, CDCl$_3$) δ -120.4 (ddd (J_{F-H} 10.7, 7.6, 5.1 Hz), C2*F*). m/*z* [M+H]$^+$ calculated: 230.1340, found: 230.1343.

*3.7.2.3 (S)-3-(2-phenoxyisopropylamino)toluene (S)-**3c***

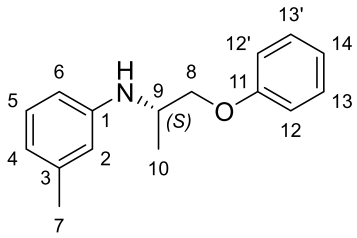

From (*S*)-2-phenoxyisopropylamine hydrochloride[1] (159.6 mg, 0.850 mmol) and 3-bromotoluene (123.8 µL, 174.5 mg, 1.02 mmol), using *t*BuXPhos-Pd-G3 (13.5 mg, 17.0 µmol, 2 mol%), and sodium *t*-butoxide (163.4 mg, 1.70 mmol). Purified by flash chromatography on silica gel using hexane/ethyl acetate (10:0 to 9:1 *v/v*), giving (*S*)-3-(2-phenoxyisopropylamino)toluene as a pale-yellow oil (125.0 mg, 0.518 mmol, 61% yield). ^{1}H-NMR (400 MHz, CDCl$_3$) δ 1.37 (3 H, d (*J* 6.3 Hz), C10H_3), 2.28 (3 H, s, C7H_3), 3.85–3.94 (2 H, m, C9*H*+C8H_a), 4.00–4.07 (1 H, m, C8H_b), 6.47–6.52 (2 H, m, C2*H*+6*H*), 6.56 (1 H, d (*J* 7.5 Hz), C4*H*), 6.91 (2 H, dd (*J* 8.7, 1.2 Hz), C12*H*+C12'*H*), 6.97 (1 H, tt (*J* 7.4, 1.1 Hz), C14*H*), 7.08 (1 H, td (*J* 7.4, 1.2 Hz), C5*H*), 7.29 (2 H, dd (*J* 8.7, 7.3 Hz), C13*H*+C13'*H*); ^{13}C-NMR (101 MHz, CDCl$_3$) δ 18.2 (*C*10), 21.6 (*C*7), 48.2 (*C*9), 70.8 (*C*8), 110.7 (*C*6), 114.3 (*C*2), 114.6 (*C*12+*C*12'), 118.7 (*C*4), 120.9 (*C*14), 129.2 (*C*5), 129.5 (*C*13+*C*13'), 139.2 (*C*3), 146.9 (*C*1), 158.8 (*C*11). m/*z* [M+H]$^+$ calculated: 242.1539 , found: 242.1545.

*3.7.2.4 3-(hexan-2-amino)toluene **3d***

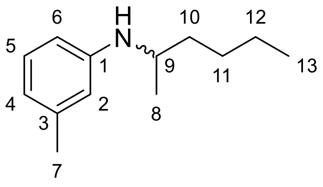

From 2-aminohexane (132.4 µL, 1.00 mmol) and 3-bromotoluene (145.6 µL, 205.2 mg, 1.20 mmol), using *t*BuXPhos-Pd-G3 (31.5 mg, 40.0 µmol, 4 mol%). Purified by flash chromatography on silica gel using hexane/ethyl acetate (100:0 to 98:2 *v/v*), giving 3-(hexan-2-amino)toluene as a slightly hazy yellow oil (156.2 mg, 0.816 mmol, 82% yield). ^{1}H-NMR (400 MHz, CDCl$_3$) δ 0.91 (3 H, t (*J* 6.8 Hz), C13H_3), 1.17 (3 H, d (*J* 6.3 Hz), C8H_3), 1.21–1.48 (5 H, m, C11H_2+12H_2+C10H_a), 1.51-1.63 (1 H, m, C10H_b), 2.27 (3 H, s, C7*H*), 3.44 (1 H, h (*J* 6.2 Hz), C9*H*), 6.37–6.43 (2 H, m, C2*H*+C6*H*), 6.50 (1 H, d (*J* 7.4 Hz), C4*H*), 7.05 (1 H, td (*J* 7.3, 1.3 Hz), C5*H*); ^{13}C-NMR (101 MHz, CDCl$_3$) δ 14.1 (*C*13), 20.8 (*C*8), 21.6 (*C*7), 22.8 (*C*12), 28.3 (*C*11), 36.9 (*C*10), 48.5 (*C*9), 110.3 (*C*6), 114.0 (*C*2), 117.8 (*C*4), 129.1 (*C*5), 139.0 (*C*3), 147.6 (*C*1). m/*z* [M+H]$^+$ calculated: 192.1747, found: 192.1748.

3.7.2.5 3-(1-(thiazol-2-yl)ethylamino)toluene **3e**

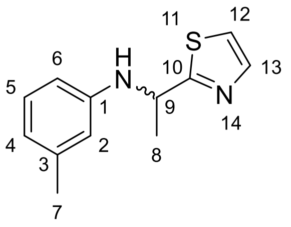

From 1-(thiazol-2-yl)ethylamine (128.2 mg, 1.00 mmol) and 3-bromotoluene (145.6 µL, 205.2 mg, 1.20 mmol). Purified by flash chromatography on silica gel using hexane/ethyl acetate (100:0 to 8:2 *v/v*), giving an impure bees-wax-like residue (150.9 mg) which was recrystalized from hexane with a drop of deuterated chloroform, giving 3-(1-(thiazol-2-yl)ethylamino)toluene as short off-white needles (110.1 mg, 0.504 mmol, 50% yield). ^{1}H-NMR (400 MHz, $CDCl_3$) *δ* 1.66 (3 H, d (*J* 6.7 Hz), C8H_3), 2.24 (3 H, s, C7H_3), 4.06 (1 H, br s, N*H*), 4.88 (1 H, q (*J* 6.7 Hz), C9*H*), 6.42 (1 H, dd (*J* 8.0, 2.5 Hz), C6*H*), 6.46 (1 H, t (*J* 2.0 Hz), C2*H*), 6.57 (1 H, d (*J* 7.4 Hz), C4*H*), 7.04 (1 H, t (*J* 7.7 Hz), C5*H*), 7.21 (1 H, d (*J* 3.3 Hz), C12*H*), 7.73 (1 H, d (*J* 3.3 Hz), C13*H*); ^{13}C-NMR (101 MHz, $CDCl_3$) *δ* 21.6 (*C*7), 23.5 (*C*8), 52.4 (*C*9), 110.6 (*C*6), 114.4 (*C*2), 118.7 (*C*12), 119.5 (*C*4), 129.1 (*C*5), 139.1 (*C*3), 142.7 (*C*13), 146.5 (*C*1), 177.5(d (*C*10). m/*z* [M+H]$^+$ calculated: 219.0950, found: 219.0952.

3.7.2.6 3-(o-fluoro-α-methylbenzylamino)pyridine **6a**

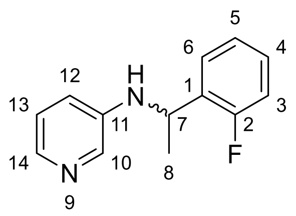

From *o*-fluoro-α-methylbenzylamine (130.2 µL, 1.00 mmol) and 3-bromopyridine (115.6 µL, 189.6 mg, 1.20 mmol). Purified by flash chromatography on silica gel using hexane/ethyl acetate (5:5 to 2:8 *v/v*), giving 3-(*o*-fluoro-α-methylbenzylamino)pyridine as an off-white powder (177.8 mg, 0.822 mmol, 82% yield). ^{1}H-NMR (400 MHz, $CDCl_3$) *δ* 1.56 (3 H, d (*J* 6.7 Hz), C8H_3), 4.24 (1 H, br s, N*H*), 4.75–4.84 (1 H, m, C7*H*), 6.73 (1 H, ddd (*J* 8.3, 2.9, 1.3 Hz), C12*H*), 6.98 (1 H, dd (*J* 8.3, 4.7 Hz), C13*H*), 7.01–7.09 (2 H, m, C3*H*+C5*H*), 7.16–7.24 (1 H, m, C4*H*), 7.32 (1 H, td (*J* 7.8, 1.9 Hz), C6*H*), 7.90 (1 H, dd (*J* 4.8, 1.3 Hz), C14*H*), 8.01 (1 H, d (*J* 3.0 Hz), C10*H*); ^{13}C-NMR (101 MHz, $CDCl_3$) *δ* 23.2 (*C*8), 47.4 (d ($J_{C\text{-}F}$ 2.9 Hz), *C*7), 115.7 (d ($J_{C\text{-}F}$ 21.8 Hz), *C*3), 119.0 (*C*12), 123.7 (*C*13), 124.5 (d ($J_{C\text{-}F}$ 3.6 Hz), *C*5), 127.0 (d ($J_{C\text{-}F}$ 4.4 Hz), *C*6), 128.7 (d ($J_{C\text{-}F}$ 8.1 Hz), *C*4), 130.7 (d ($J_{C\text{-}F}$ 13.1 Hz), *C*1), 136.4 (*C*10), 138.7 (*C*14), 142.9 (*C*11), 160.5 (d ($J_{C\text{-}F}$ 245.1 Hz), *C*2); ^{19}F-NMR (376 MHz, $CDCl_3$) *δ* -120.3 (ddd ($J_{F\text{-}H}$ 10.8, 7.6, 5.2 Hz), C2*F*). m/*z* [M+H]$^+$ calculated: 217.1136, found: 217.1143.

3.7.2.7 4-(o-fluoro-α-methylbenzylamino)isoquinoline **6b**

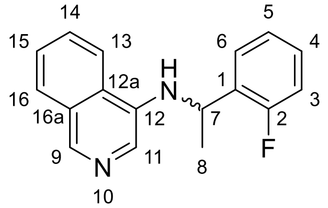

From *o*-fluoro-α-methylbenzylamine (130.2 µL, 1.00 mmol) and 4-bromoisoquinoline (249.7 mg, 1.20 mmol). Purified by flash chromatography on silica gel using hexane/ethyl acetate (9:1 to 7:3 *v/v*), giving two fractions of 4-(*o*-fluoro-α-methylbenzylamino)isoquinoline, an impure off-white powder (97.7 mg) and a coral powder (88.2 mg, 0.331 mmol, 33% yield). ^{1}H-NMR (400 MHz, $CDCl_3$) *δ* 1.69 (3 H, d (*J* 6.7 Hz), C8H_3), 4.65 (1 H, br d (*J* 5.9 Hz), N*H*), 5.04 (1 H, p (*J* 6.4 Hz), C7*H*), 6.99–7.09 (2 H, m, C3*H*+C5*H*), 7.20 (1 H, dddd (*J* 8.8, 7.2, 5.3, 1.8 Hz), C4*H*), 7.36 (1 H, td (*J* 7.7, 1.8 Hz), C6*H*), 7.58 (1 H, ddd (*J* 8.0, 6.9, 1.1 Hz), C15*H*), 7.66 (1 H, s, C11*H*), 7.69 (1 H, ddd (*J* 8.4, 6.8,

1.4 Hz), C14*H*), 7.89 (1 H, d (*J* 8.1 Hz), C16*H*), 7.92 (1 H, d (*J* 8.6 Hz), C13*H*), 8.64 (1 H, s, C9*H*); ^{13}C-NMR (101 MHz, CDCl$_3$) δ 23.4 (*C*8), 47.8 (d ($J_{C\text{-}F}$ 3.2 Hz), *C*7), 115.8 (d ($J_{C\text{-}F}$ 21.9 Hz), *C*3), 119.0 (*C*13), 124.5 (d ($J_{C\text{-}F}$ 3.3 Hz), *C*5), 124.5 (*C*11), 125.8 (*C*12a), 126.9 (d ($J_{C\text{-}F}$ 5.1 Hz), *C*6), 126.9 (*C*15), 128.2 (*C*16), 128.4 (*C*16a), 128.7 (d ($J_{C\text{-}F}$ 8.5 Hz), *C*4), 129.1 (*C*14), 130.6 (d ($J_{C\text{-}F}$ 13.0 Hz), *C*1), 136.1 (*C*12), 142.1 (*C*9), 160.6 (d ($J_{C\text{-}F}$ 245.6 Hz), *C*2); ^{19}F-NMR (376 MHz, CDCl$_3$) δ -120.0 (ddd ($J_{F\text{-}H}$ 10.8, 7.7, 5.3 Hz), C2*F*). m/*z* [M+H]$^+$ calculated: 267.1292, found: 267.1305.

*3.7.2.8 4-(o-fluoro-α-methylbenzylamino)benzonitirile **6c***

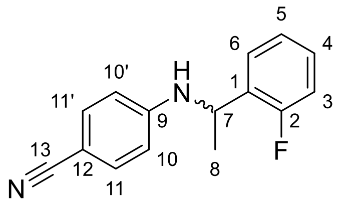

From *o*-fluoro-α-methylbenzylamine (130.2 µL, 1.00 mmol) and 4-bromobenzonitrile (218.4 mg, 1.20 mmol). Purified by flash chromatography on silica gel using hexane/ethyl acetate (100:0 to 85:15 to 80:20 *v/v*), giving an impure bright yellow powder (153.6 mg), which was recrystalized from ethyl acetate/hexane, giving 4-(*o*-fluoro-α-methylbenzylamino)benzonitirile as a bright orange-yellow powder (127.2 mg, 0.529 mmol, 53% yield). ^{1}H-NMR (400 MHz, CDCl$_3$) δ 1.61 (3 H, d (*J* 6.7 Hz), C8H_3), 4.69 (1 H, br s, N*H*), 4.88 (1 H, q (*J* 6.7 Hz), C7*H*), 6.53 (2 H, d (*J* 8.4), C10*H*+C10′*H*), 7.05–7.16 (2 H, m, C3*H*+C5*H*), 7.26 (1 H, ddd (*J* 7.4, 5.3, 1.9 Hz), C4*H*), 7.32 (1 H, td (*J* 8.1, 2.0 Hz), C6*H*), 7.38 (2 H, d (*J* 8.6 Hz), C11*H*+C11′*H*); ^{13}C-NMR (101 MHz, CDCl$_3$) δ 23.0 (*C*8), 47.3 (d ($J_{C\text{-}F}$ 3.0 Hz), *C*7), 99.3 (*C*12), 112.9 (*C*10+*C*10′), 115.8 (d ($J_{C\text{-}F}$ 21.7 Hz), *C*3), 120.3 (*C*13), 124.6 (d ($J_{C\text{-}F}$ 3.5 Hz), *C*5), 126.9 (d ($J_{C\text{-}F}$ 4.4 Hz), *C*6), 129.0 (d ($J_{C\text{-}F}$ 8.3 Hz), *C*4), 130.2 (d ($J_{C\text{-}F}$ 13.0 Hz), *C*1), 133.7 (*C*11+C11′), 149.9 (*C*9), 160.4 (d ($J_{C\text{-}F}$ 245.4 Hz), *C*2); ^{19}F-NMR (376 MHz, CDCl$_3$) δ -120.1 (ddd ($J_{F\text{-}H}$ 10.8, 7.5, 5.2 Hz), C2*F*). m/*z* [M+H]$^+$ calculated: 241.1136, found: 241.1136.

*3.7.2.9 4-(o-fluoro-α-methylbenzylamino)anisole **6d***

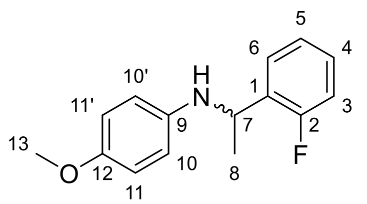

From *o*-fluoro-α-methylbenzylamine (130.2 µL, 1.00 mmol) and 4-bromoanisole (150.2 µL, 224.4 mg, 1.20 mmol). Purified by flash chromatography on silica gel using hexane/ethyl acetate (100:0 to 95:5 to 90:10 *v/v*), giving 4-(*o*-fluoro-α-methylbenzylamino)anisole as a bright green-yellow oil (188.1 mg, 0.767 mmol, 77% yield). ^{1}H-NMR (400 MHz, CDCl$_3$) δ 1.53 (3 H, d (*J* 6.7 Hz), C8H_3), 3.70 (3 H, s, C13H_3), 4.75 (1 H, q (*J* 6.7 Hz), C7*H*), 6.46–6.53 (2 H, m, C10*H*+C10′*H*), 6.68–6.73 (2 H, m, C11*H*+C11′*H*), 7.00–7.09 (2 H, m, C3*H*+C5*H*), 7.19 (1 H, tdd (*J* 7.3, 5.2, 1.8 Hz), C4*H*), 7.37 (1 H, td (*J* 7.6, 1.8 Hz), C6*H*); ^{13}C-NMR (101 MHz, CDCl$_3$) δ 23.4 (*C*8), 48.3 (d ($J_{C\text{-}F}$ 2.7 Hz), *C*7), 55.7 (*C*13), 114.6 (*C*10+*C*10′), 114.8 (*C*11+C11′), 115.4 (d ($J_{C\text{-}F}$ 21.9 Hz), *C*3), 124.4 (d ($J_{C\text{-}F}$ 3.5 Hz), *C*5), 127.3 (d ($J_{C\text{-}F}$ 5.1 Hz), *C*6), 128.2 (d ($J_{C\text{-}F}$ 8.5 Hz), *C*4), 131.9 (d ($J_{C\text{-}F}$ 13.4 Hz), *C*1), 141.0 (*C*9), 152.1 (*C*12), 160.5 (d ($J_{C\text{-}F}$ 244.5 Hz), *C*2); ^{19}F-NMR (376 MHz, CDCl$_3$) δ -120.5 (ddd ($J_{F\text{-}H}$ 10.6, 7.7, 5.4 Hz), C2*F*). m/*z* [M+H]$^+$ calculated: 246.1289, found: 246.1293.

3.8 *Analytical techniques*

3.8.1 *Reverse-phase HPLC analysis of conversions*

Samples were analysed using a ThermoFisher Ultimate 3000 Reverse-phase HPLC (diode array detector) on a Waters XBridge C18 column (3.5 µm, 2.1 x 150 mm) at 45 °C with a flow rate of 0.8 mL/min. Injection volume 2-5 µL. The following methods were used (A: 0.1% TFA in water, B: 0.1% TFA in acetonitrile):

Gradient 1: 0 min 95% A 5% B; 1 min 95% A 5% B; 5 min 5% A 95% B; 5.10 min 0% A 100% B; 6.60 min 0% A 100% B; 7 min 95% A 5% B; 10 min 95% A 5% B. UV chromatograms were obtained at 210, 240, 250, 265, and 290. Retention times in min: benzaldehyde (3.54), benzylamine (1.06), phenoxyacetone (3.86), 2-phenoxyisopropylamine (3.05), *o*-F-MBA (2.067), *o*-F-acetophenone (4.03), 3-benzylaminotoluene (4.00), 3-isopropylaminotoluene (3.29), 3-(*o*-F-methylbenzylamino)toluene (4.48), 3-(hexan-2-amino)toluene (4.02), 3-(2-phenoxyisopropylamino)toluene (4.18), 3-(*o*-F-methylbenzylamino)pyridine (3.63), 4-(*o*-F-methylbenzylamino)isoquinoline (3.92), 4-(*o*-F-methylbenzylamino)benzonitrile (4.97), 4-(*o*-F-methylbenzylamino)anisole (3.82), 3-bromotoluene (5.23), 4-bromoanisole (4.90), 3-bromopyridine (1.08), 4-bromoisoquinoline (3.53), 4-bromobenzonitrile (4.46).

Gradient 2: 0 min 100% A 0% B; 1 min 100% A 0% B; 5 min 5% A 95% B; 5.10 min 0% A 100% B; 6.60 min 0% A 100% B; 7 min 100% A 0% B; 10 min 100% A 2% B. UV chromatograms were obtained at 210, 240 and 290 nm. Retention times in min: 2-acetylthiazole (3.33), 1-(thiazol-2-yl)ethylamine (1.21), 3-(1-(thiazol-2-yl)ethylamino)toluene (4.42).

Conversions were calculated by comparing the areas of starting material and product, corrected by their response factor.

3.8.2 *Chiral GC-FID*

Biotransformations (1 mL) were basified with NaOH (5M, 100 µL) and extracted with EtOAc (2×500 µL). Extracted BHA samples (0.5 mL) or extracted biotransformations were derivatized with 20 µL each triethylamine and acetic anhydride (under these conditions *N*-arylamines were not acetylated) and analysed by GC-FID: Thermo Scientific™ Trace™ 1310 GC equipped with an Agilent CHIRASIL-DEX CB (25 m x 0.25 mm x 0.25 µm) column:

0 min 40 °C, 2 min 40 °C, 7.5 min 150 °C, 12.5 min 150 °C, 14.167 min 200 °C, 18.167 min 200 °C. Injector temperature 230 °C, split ratios 1:10 to 1:100, continuous flow 1.7 mL/min, FID temperature 250 °C, injection volume 1 µL. Helium was used as carrier gas. Retention times in min (primary amines

acetylated): (*S*)-*o*-fluoro-α-methylbenzylamine (11.0), (*R*)-*o*-fluoro-α-methylbenzylamine (11.1), (*S*)-hexan-2-amine (8.0), (*R*)-hexan-2-amine (8.1), (*S*)-2-phenoxyisopropylamine (14.6), (*R*)-2-phenoxyisopropylamine (14.7), (*S*)-1-(thiazol-2-yl)ethylamine (11.8), (*R*)-1-(thiazol-2-yl)ethylamine (11.9); hexan-2-one (4.8), (*S*)-3-(hexan-2-amino)toluene (12.1), (*R*)-3-(hexan-2-amino)toluene (12.2), 3-isopropylaminotoluene (8.5).

Conversions were calculated by comparing the areas of starting material and product, corrected by their response factor. Enantiomers assigned based on authentic standards, except for 1-(thiazol-2-yl)ethylamine, where enantiopure standards were not available. Here, the elution order (which is consistently (*S*) before (*R*) for all primary amines studied) as well the complementary selectivities of *Ts*RTA ((*R*)-selective) and HEwT ((*S*)-selective) were used to assign absolute stereochemistry.

3.8.3 Chiral RP-HPLC

3.8.3.1 Solvent-less Acetylation or Trifluoroacetylation

For purified compounds, approx. 1 mg or 1 µL were used. For EtOAc extracts of reactions, an aliquot containing approx. 1 mg was taken, the solvent removed *in vacuo*, and the remaining residue was used. To this sample were added triethylamine (20 µL) and acetic anhydride (20 µL), or a mixture (60 µL) triethylamine and trifluoroacetic anhydride (2:3 *v/v*), prepared on ice (exothermic!). After incubation at 30 °C, 900 rpm for 16-24 h (40 °C, 48 h for **6b**), 10 µL of the supernatant was diluted with 990 µL ACN/HCl (0.2%) (1:1 *v/v*) and analysed by chiral RP-HPLC.

3.8.3.2 Chiral RP-HPLC method

The samples, derivatized and prepared as described above, were analysed by reverse-phase HPLC (diode array detector) on a Phenomenex Lux Cellulose-2 chiral column (5 µm, 44.6 x 250 mm); injection volume 2-20 µL, at ambient temperature with a flow rate of 1 mL/min, with the following isocratic methods: A: 0.1% TFA in water, B: 0.1% TFA in acetonitrile (Retention times in min):

50% A, 50% B; acetylated: (*S*)-4-(*o*-F-methylbenzylamino)anisole (14.6), (*R*)-4-(*o*-F-methylbenzylamino)anisole (15.6);

55% A, 45% B; acetylated: (*S*)-3-(*o*-F-methylbenzylamino)toluene (26.3), (*R*)-3-(*o*-F-methylbenzylamino)toluene (27.8), (*S*)-3-(2-phenoxyisopropylamino)toluene (34.5), (*R*)-3-(2-phenoxyisopropylamino)toluene (37.1);

60% A, 40% B; trifluoroacetylated: (*S*)-4-(*o*-F-methylbenzylamino)benzonitrile (41.1), (*R*)-4-(*o*-F-methylbenzylamino)benzonitrile (42.6), Rot1-(*S*)-4-(*o*-F-methylbenzylamino) isoquinoline (16.4), Rot1-(*R*)-4-(*o*-F-methylbenzylamino)isoquinoline (19.7), Rot2-(*S*)-4-(*o*-F-

methylbenzylamino)isoquinoline (21.3), Rot2-(*R*)-4-(*o*-F-methylbenzylamino)isoquinoline (21.9) (Rot1, Rot2 = rotamers 1 & 2);

70% A, 30% B; no derivatization: (*S*)-3-(1-(thiazol-2-yl)ethylamino)toluene (17.6), (*S*)-3-(1-(thiazol-2-yl)ethylamino)toluene (18.3); trifluoroacetylated: (*S*)-3-(*o*-F-methylbenzylamino)pyridine (32.8), (*R*)-3-(*o*-F-methylbenzylamino)pyridine (38.2).

Absolute stereochemistry inferred from the selectivity of the enzyme.

4 Chromatograms of enantiomeric excess determination

4.1 *Biotransformations*

*4.1.1 o-fluoro-α-methylbenzylamine **2b**, acetylated (GC-FID)*

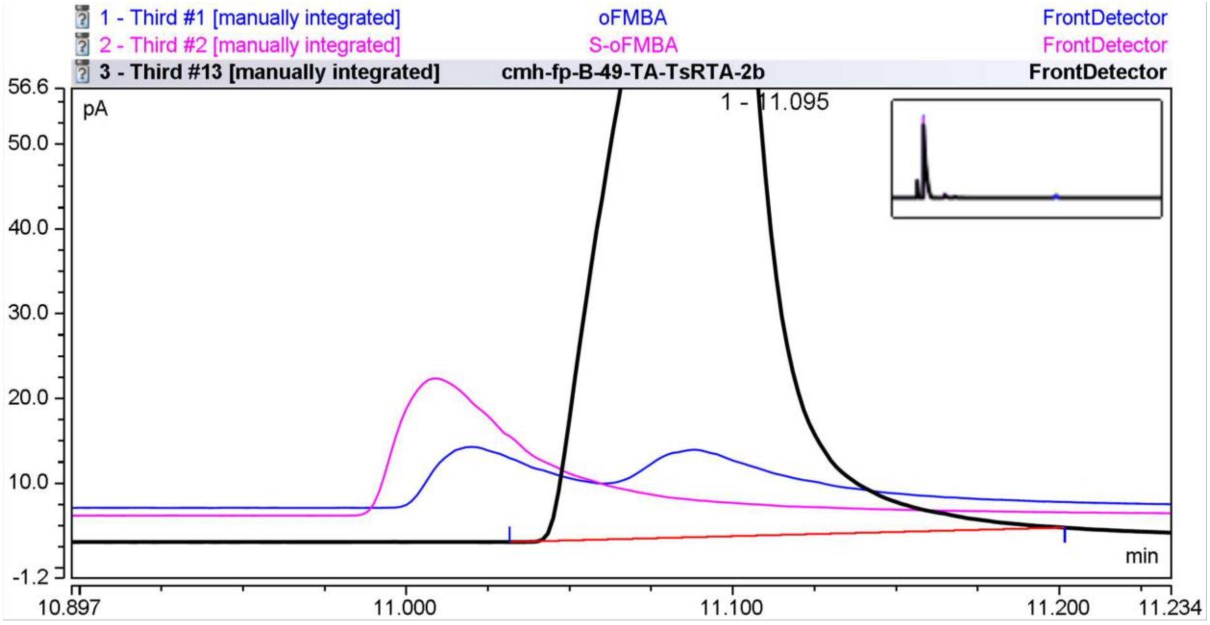

4.1.2 *2-phenoxyisopropylamine **2c**, acetylated (GC-FID)*

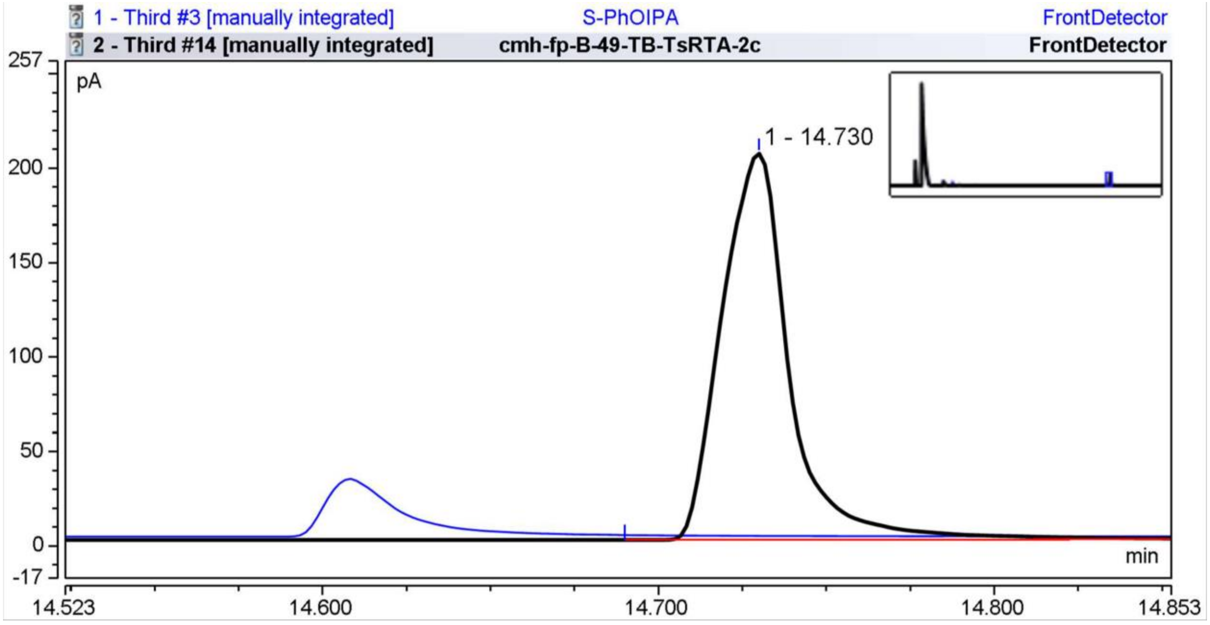

4.1.3 *hexan-2-amine **2d**, acetylated (GC-FID)*

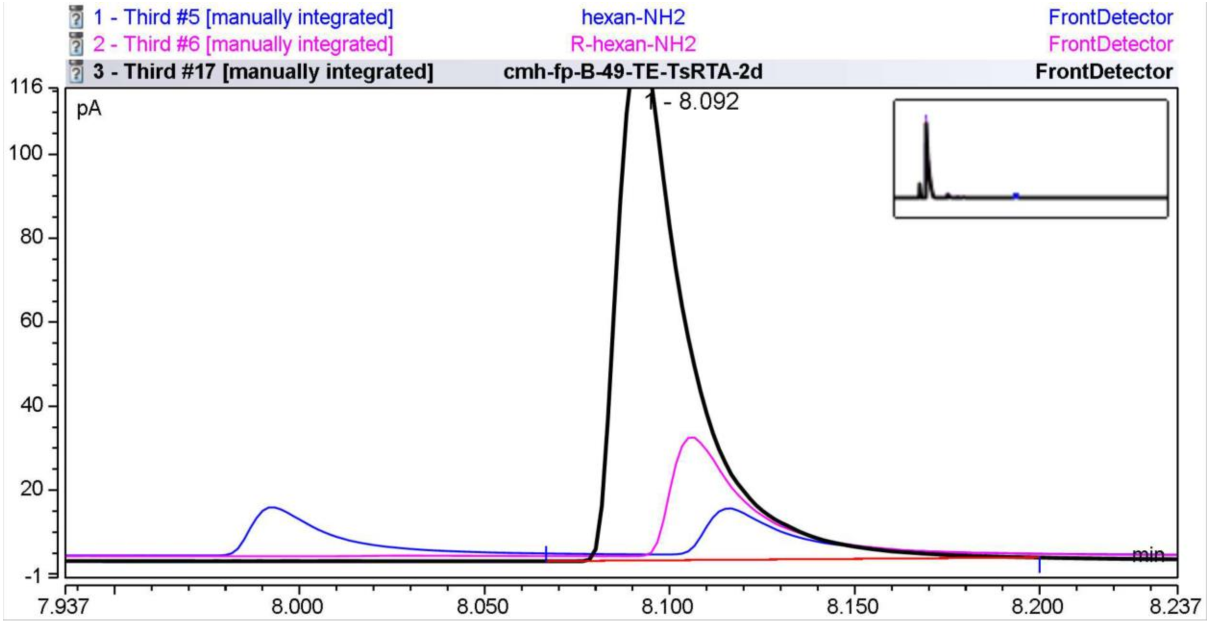

4.1.4 1-(thiazol-2-yl)ethylamine **2e**, acetylated (GC-FID)

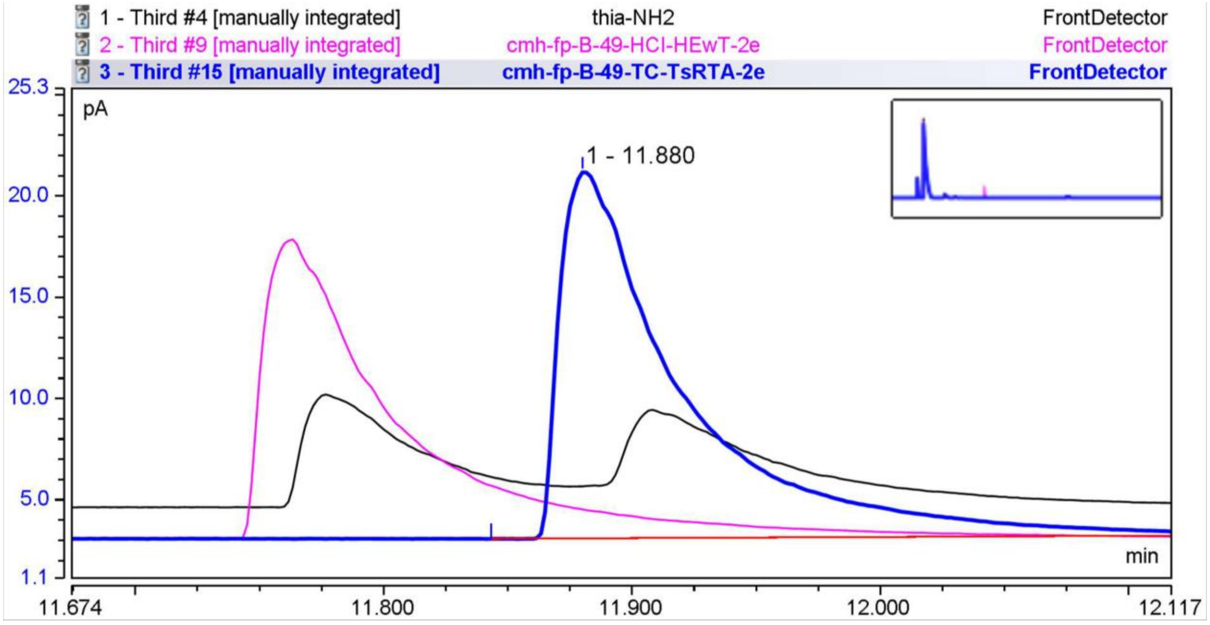

4.2 Combined Cascade

4.2.1 3-(hexan-2-amino)toluene **3d**, underivatized (GC-FID)

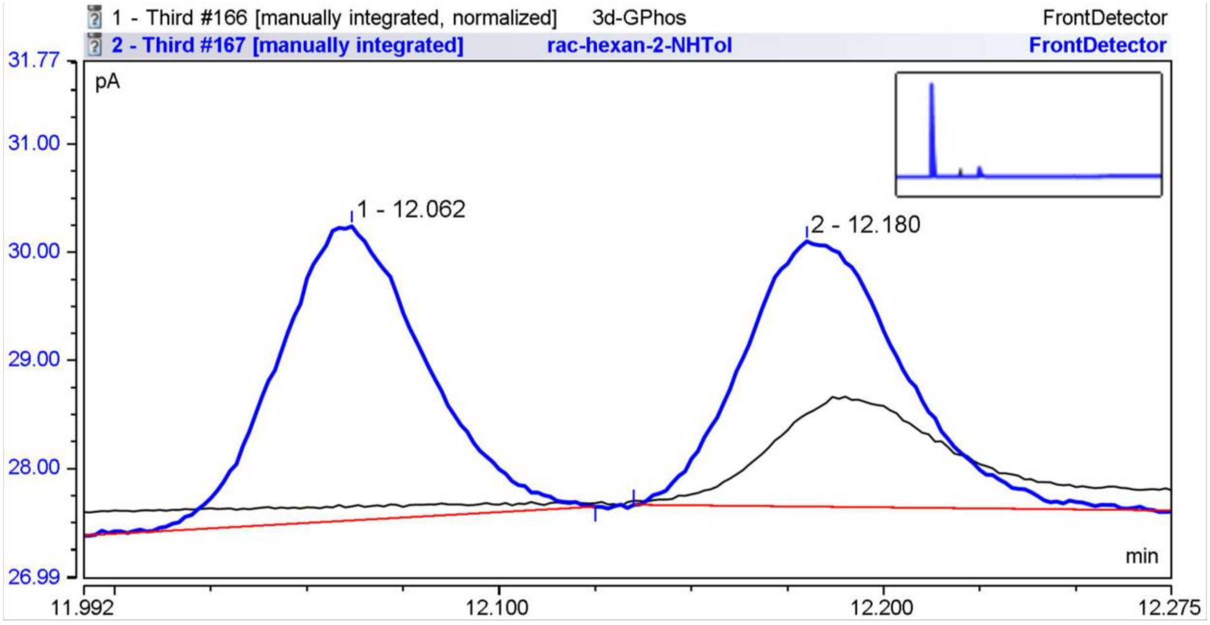

4.2.2 3-(o-F-methylbenzylamino)toluene ***3b***, acetylated (RP-HPLC)

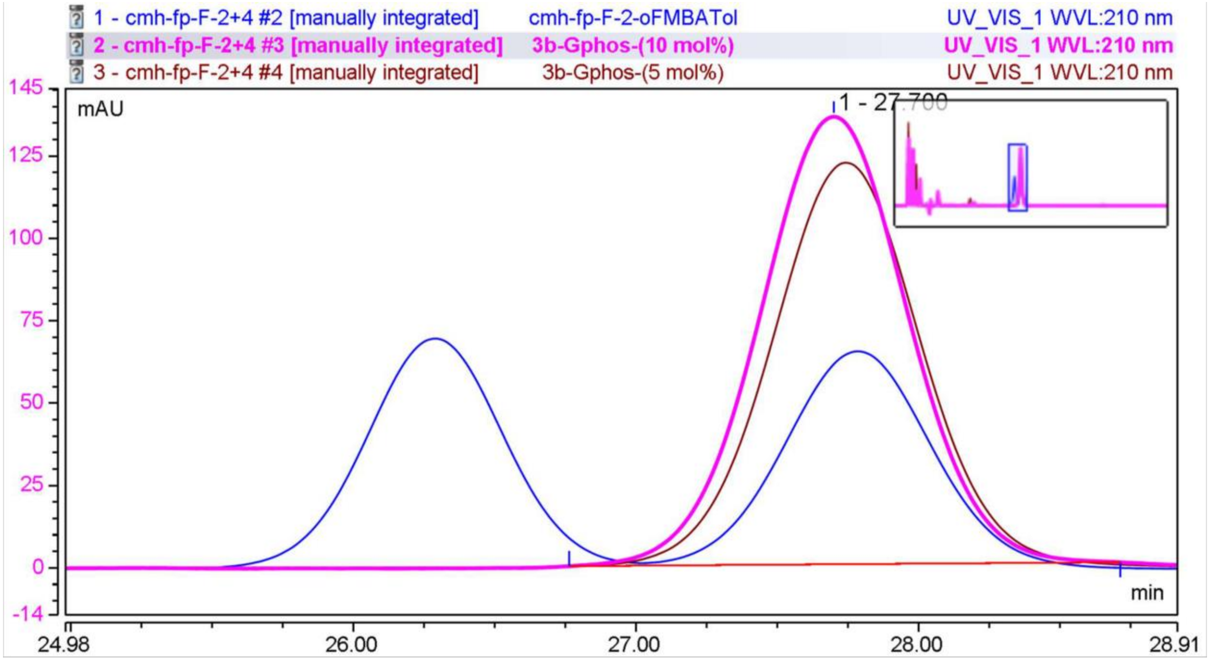

4.2.3 3-(2-phenoxyisopropylamino)toluene ***3c***, acetylated (RP-HPLC)

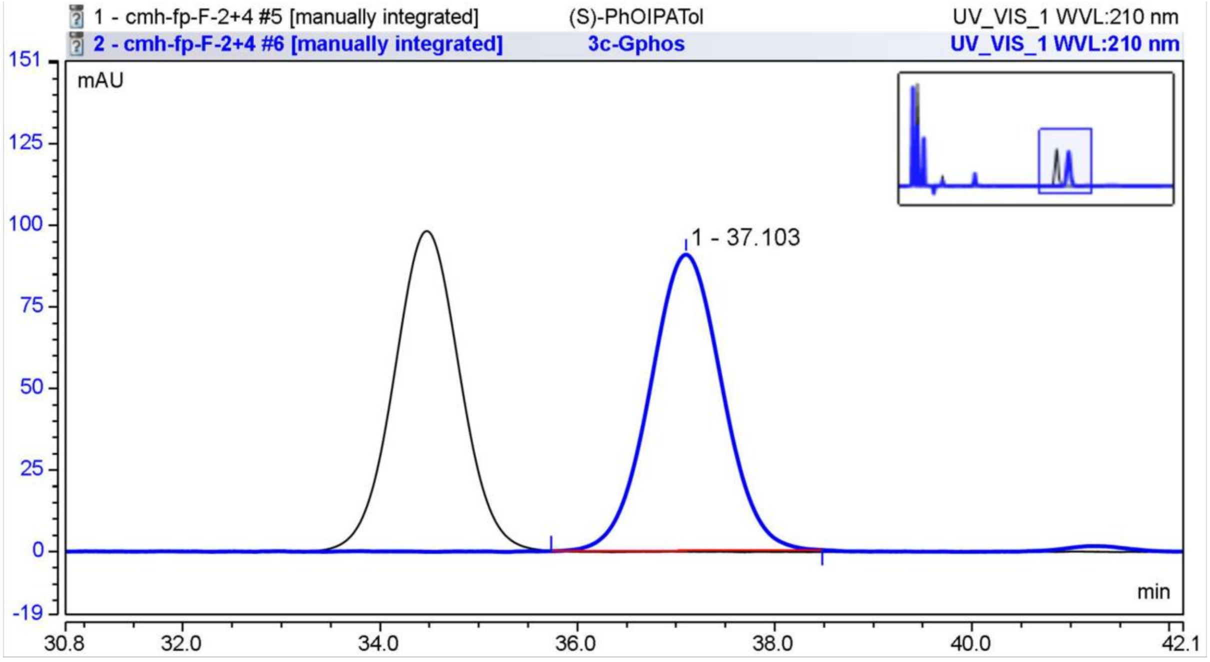

4.2.4 3-(1-(thiazol-2-yl)ethylamino)toluene ***3e***, *underivatized (RP-HPLC)*

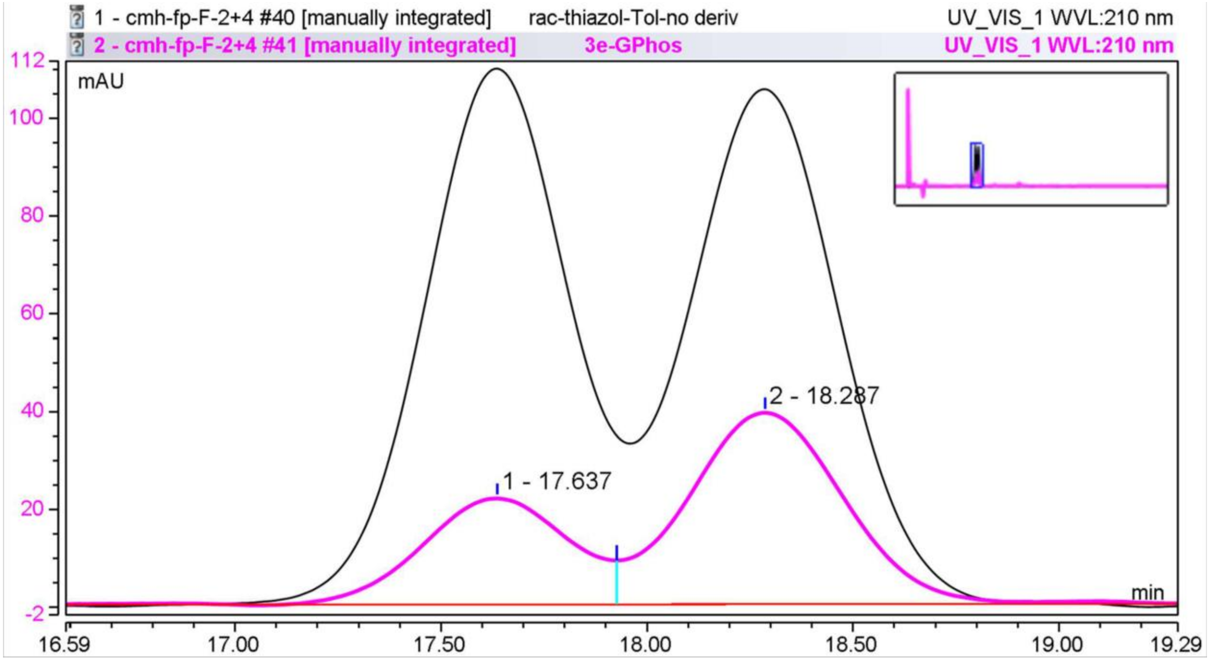

4.2.5 3-(o-F-methylbenzylamino)pyridine ***6a***, *trifluoroacetylated (RP-HPLC)*

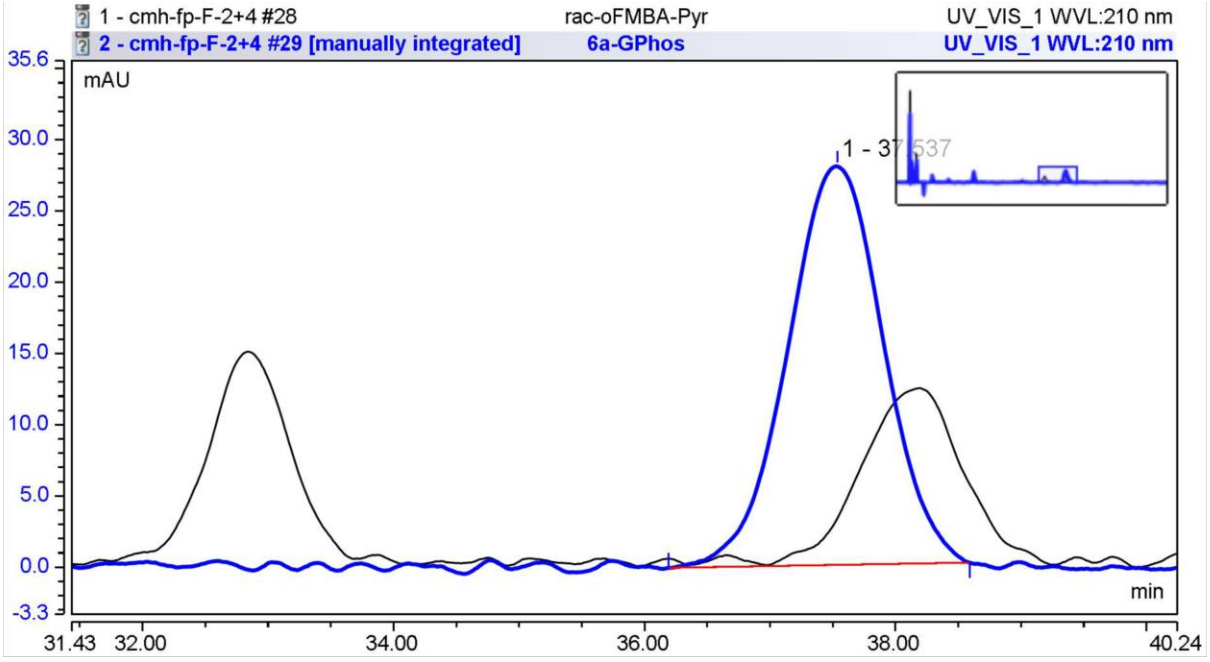

*4.2.6 4-(o-F-methylbenzylamino)isoquinoline **6b**, trifluoroacetylated (RP-HPLC)*

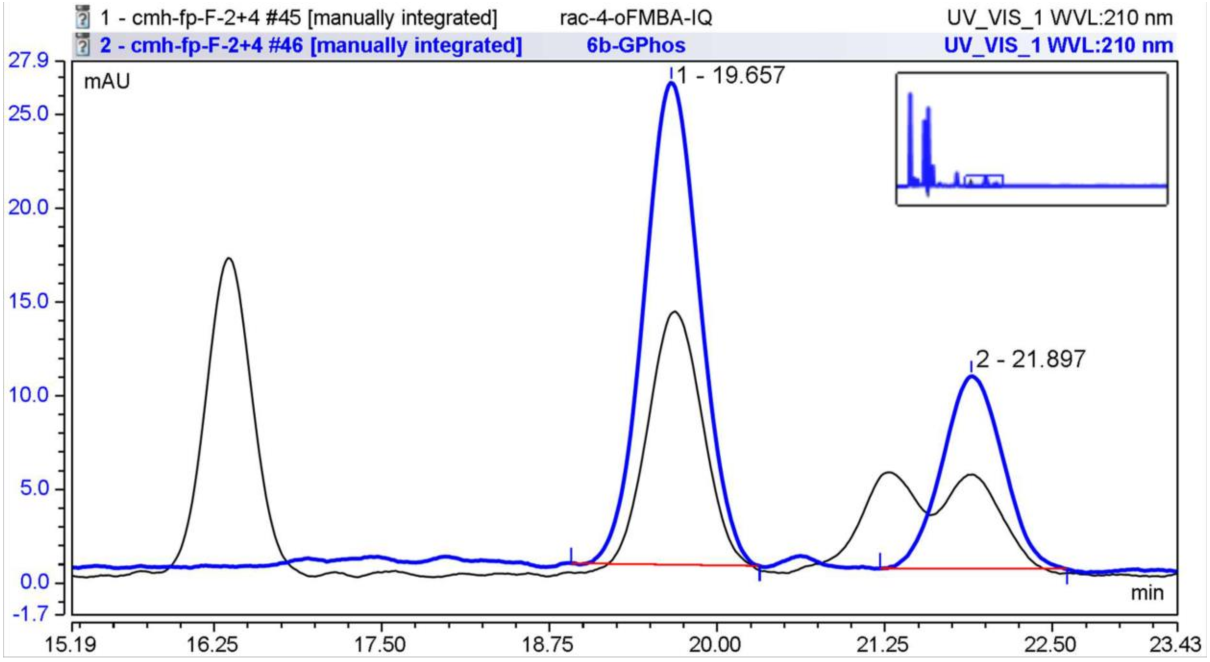

*4.2.7 4-(o-F-methylbenzylamino)benzonitrile **6c**, trifluoroacetylated (RP-HPLC)*

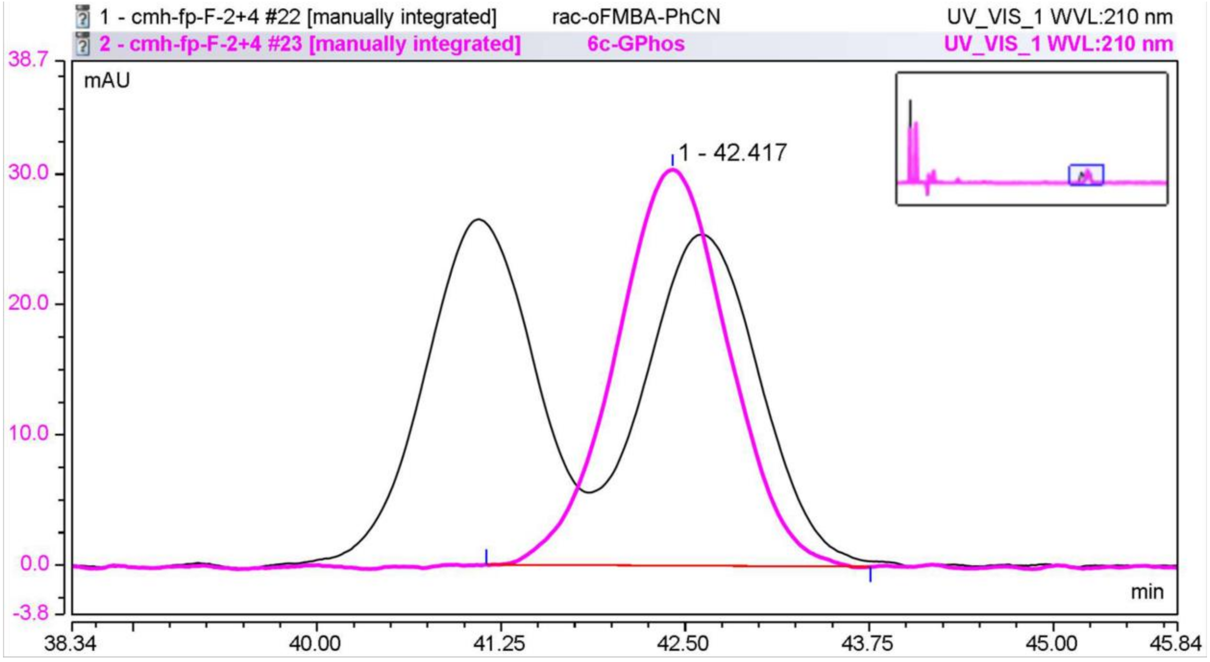

*4.2.8 4-(o-F-methylbenzylamino)anisole **6d**, acetylated (RP-HPLC)*

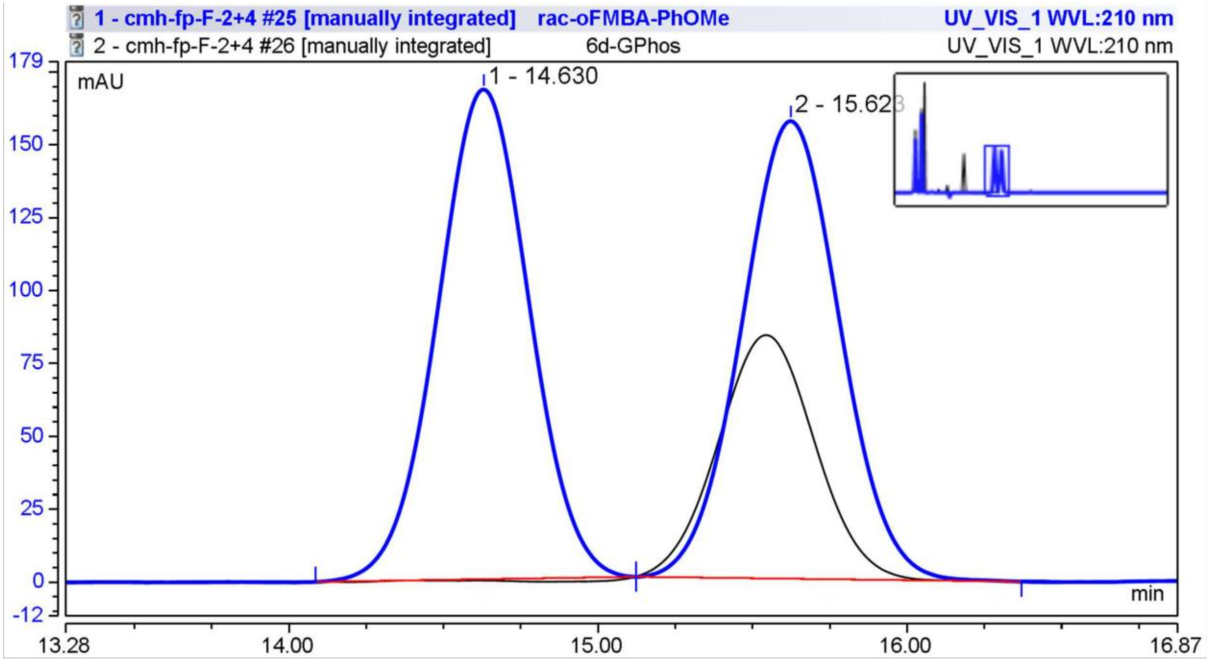

5 NMR-spectra

*5.1 (R)-3-(o-fluoro-α-methylbenzylamino)toluene (R)-**3b***

5.1.1 ^{1}H-NMR

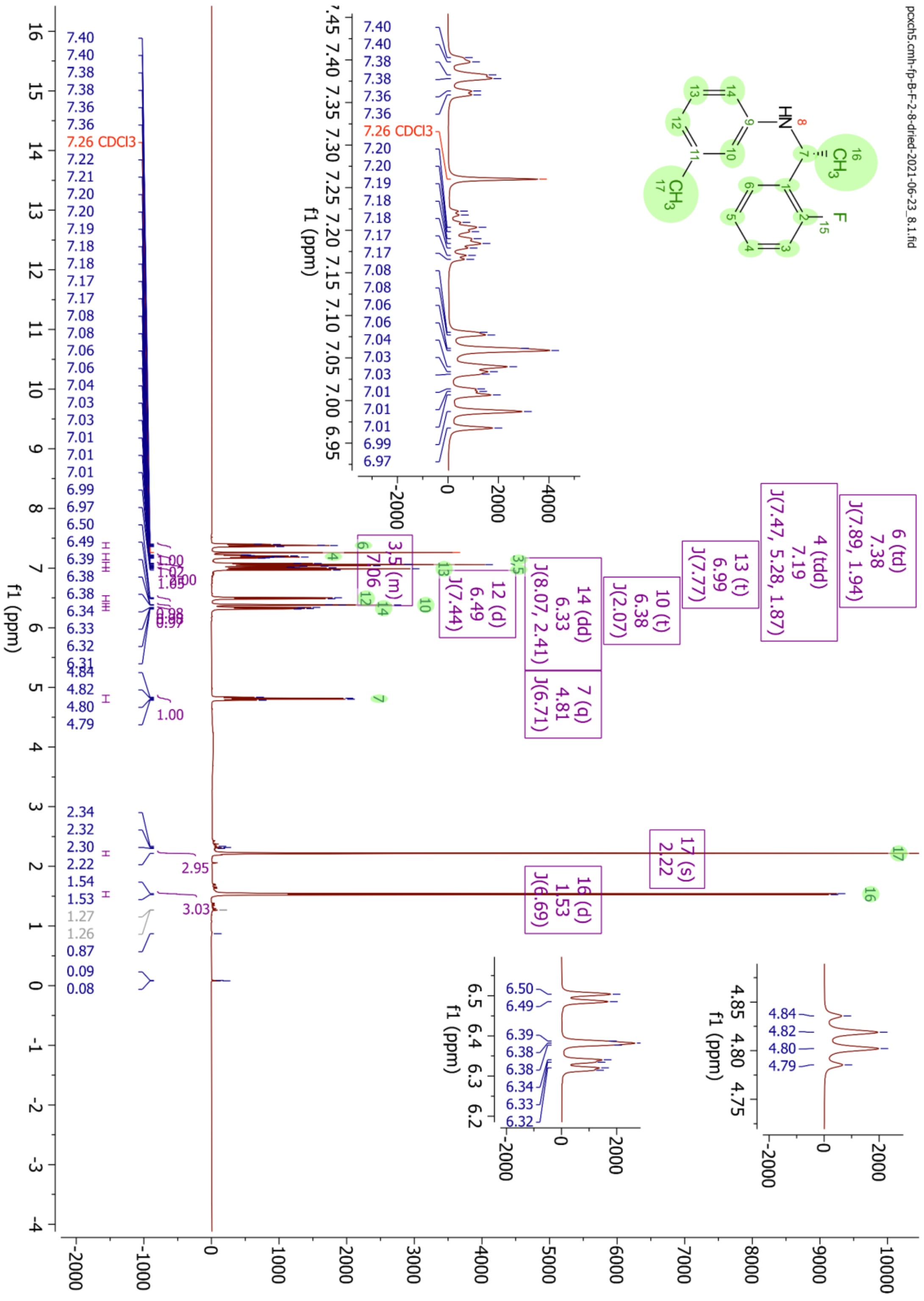

5.1.2 ^{13}C-{^{1}H}-NMR

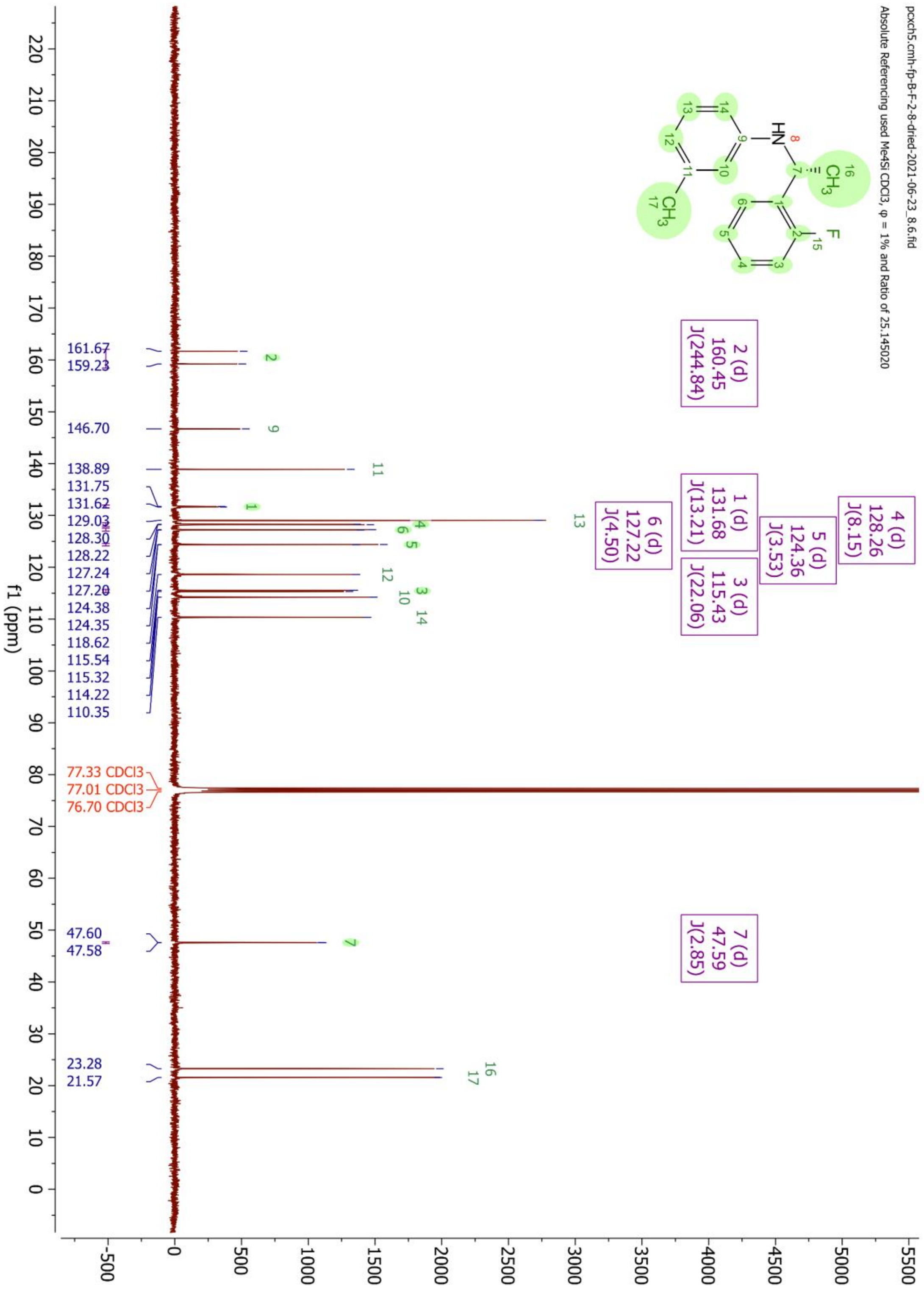

5.1.3 ^{19}F-NMR

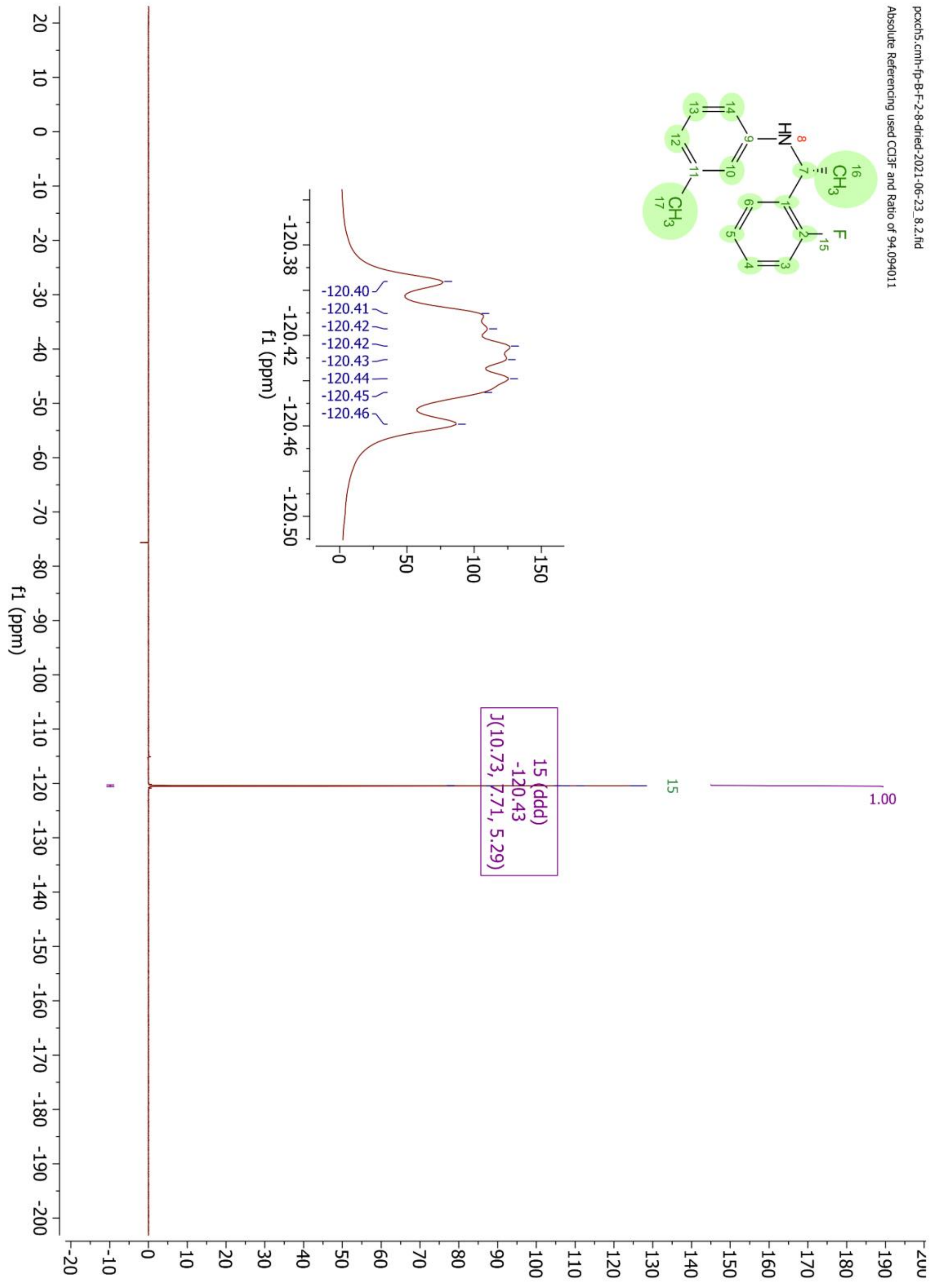
pcxch5.cmh-fp-B-F-2-8-dried-2021-06-23_8.2.fid
Absolute Referencing used CCl3F and Ratio of 94.094011
15 (ddd)
-120.43
J(10.73, 7.71, 5.29)
1.00
f1 (ppm)

5.1.4 ^1H-^1H-COSY

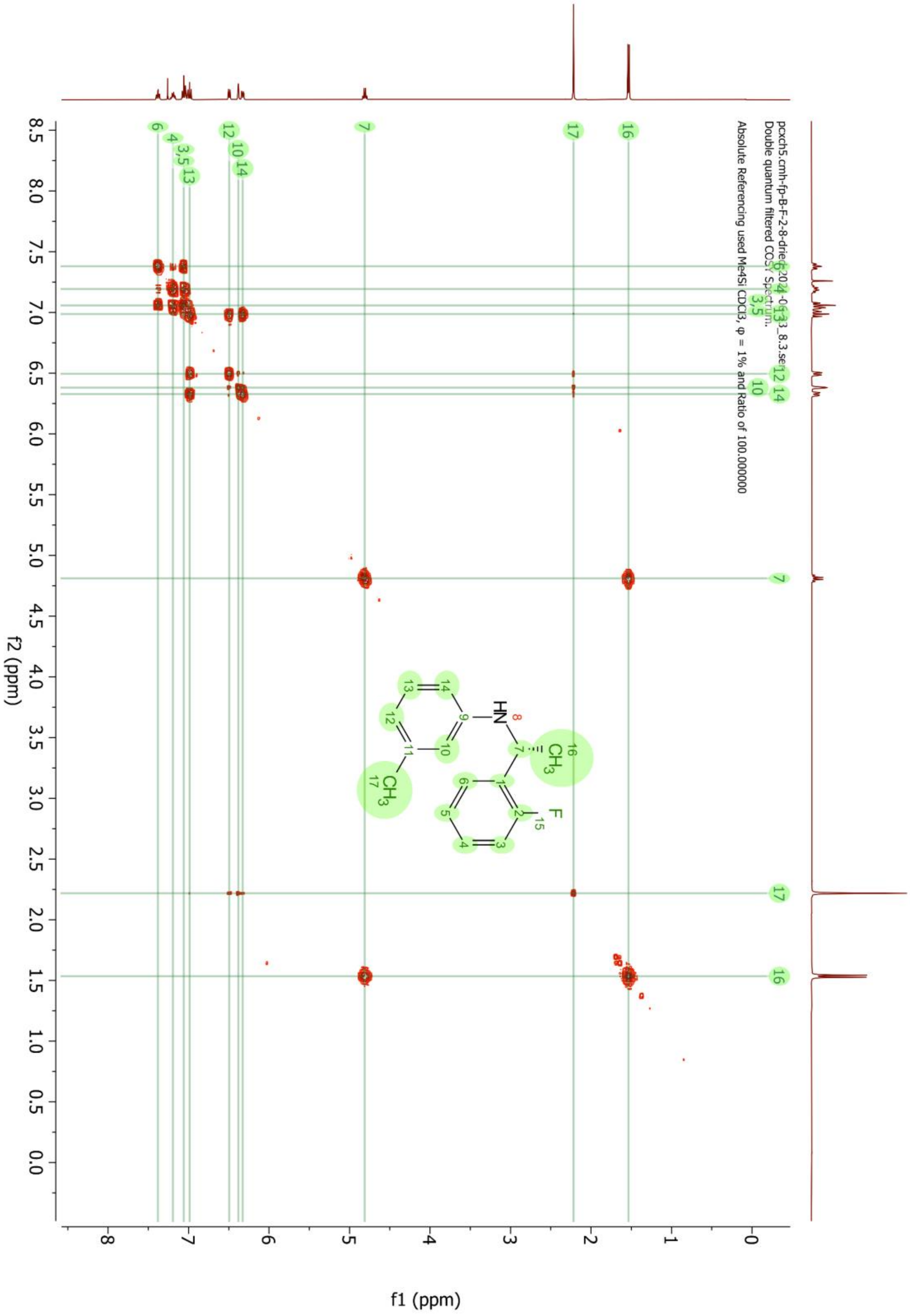

5.1.5 ¹H-¹³C-HSQC-ME

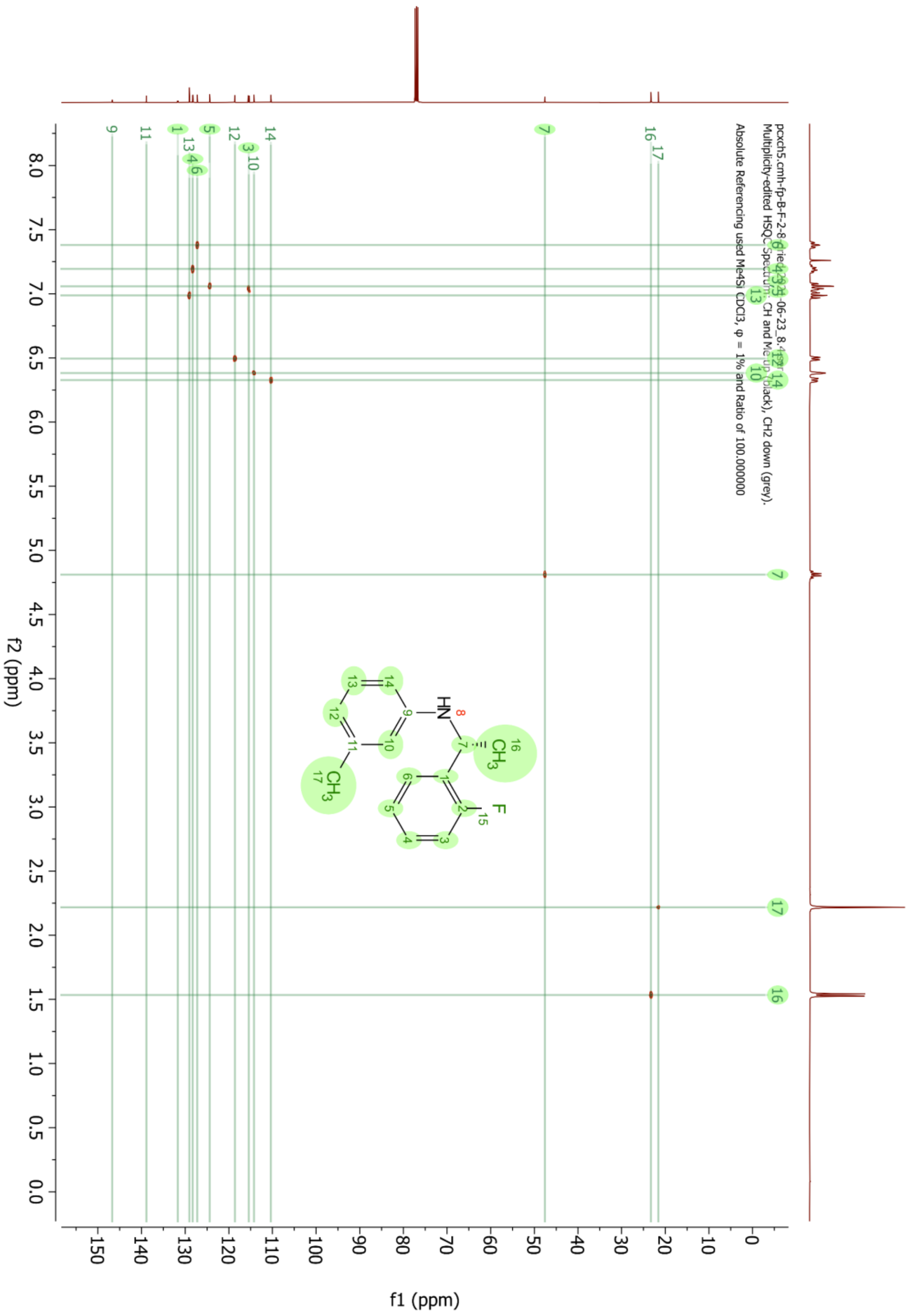

5.1.6 ^{1}H-^{13}C-HMBC

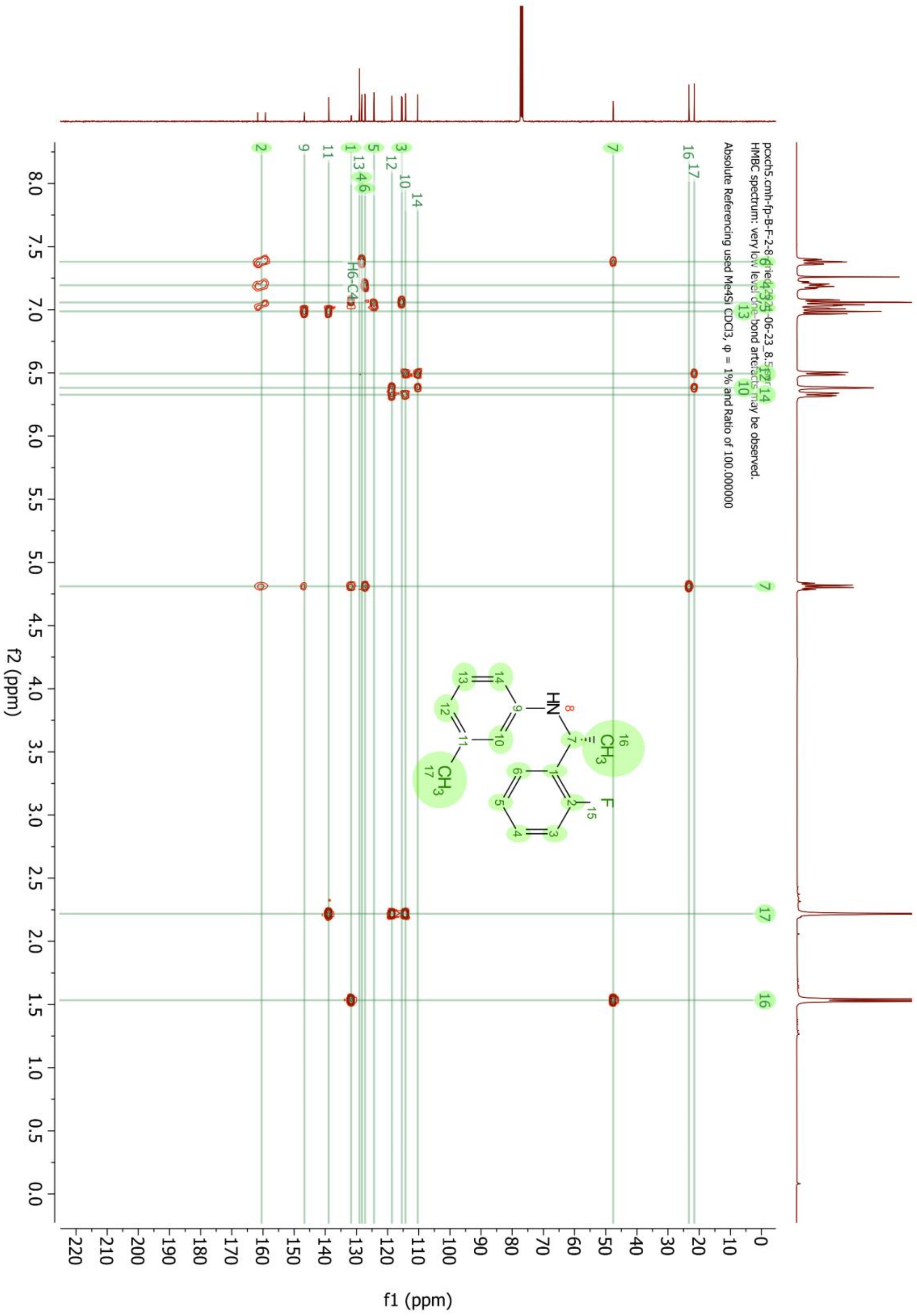

5.2 *3-benzylaminotoluene hydrochloride **3a***

5.2.1 *^{1}H-NMR*

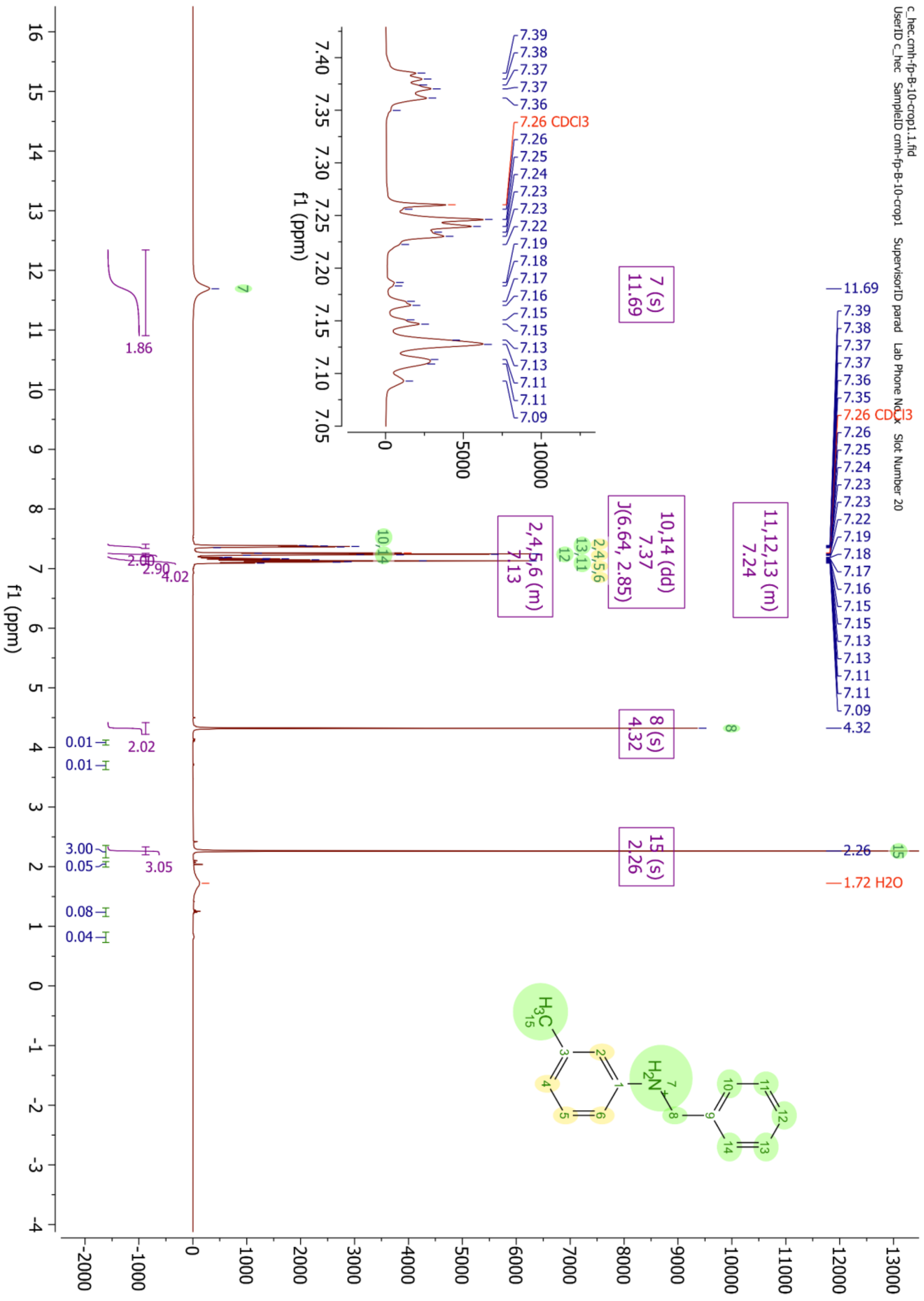

5.2.2 ^{13}C-{^{1}H}-NMR

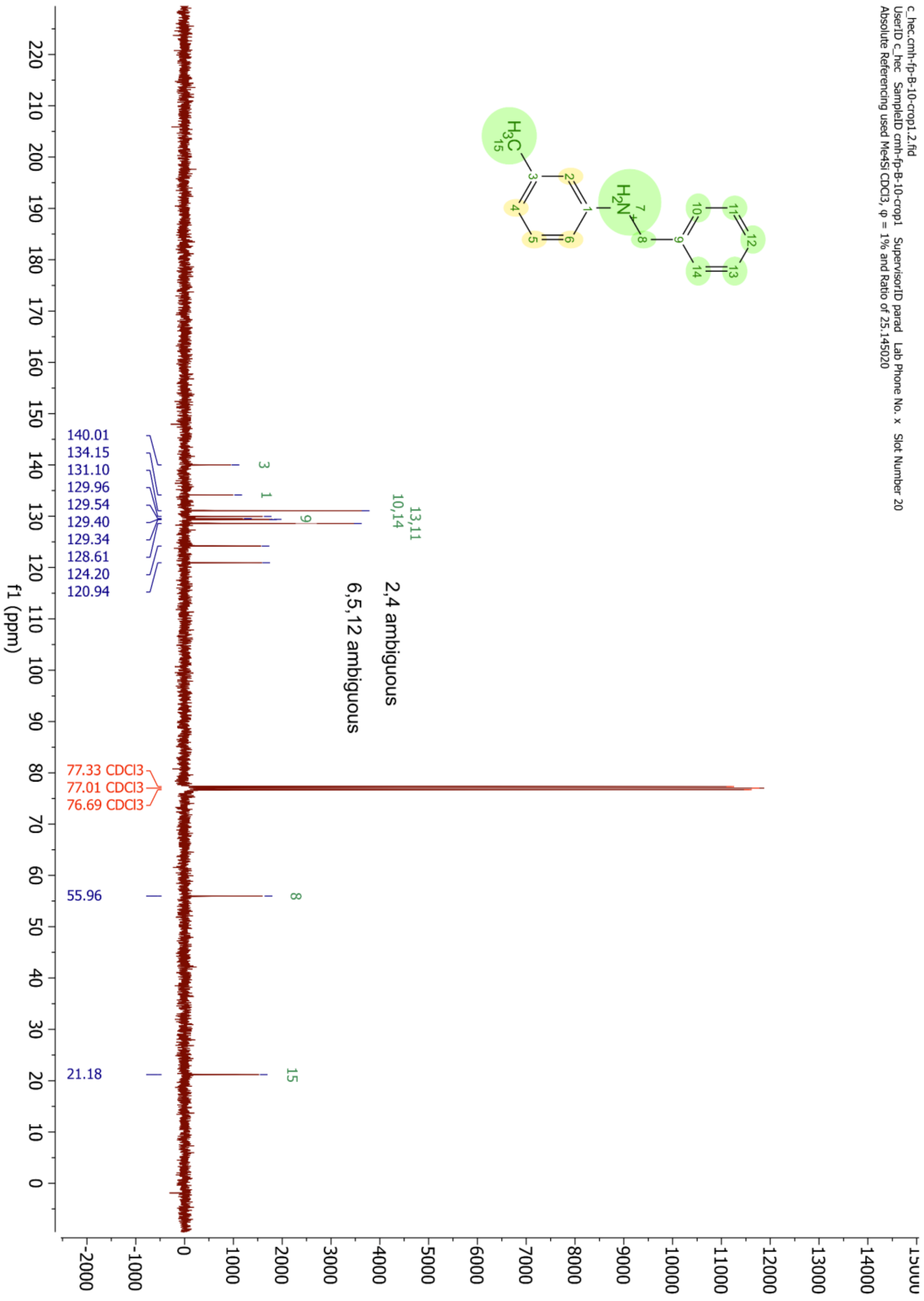

5.2.3 ^{1}H-^{1}H-COSY

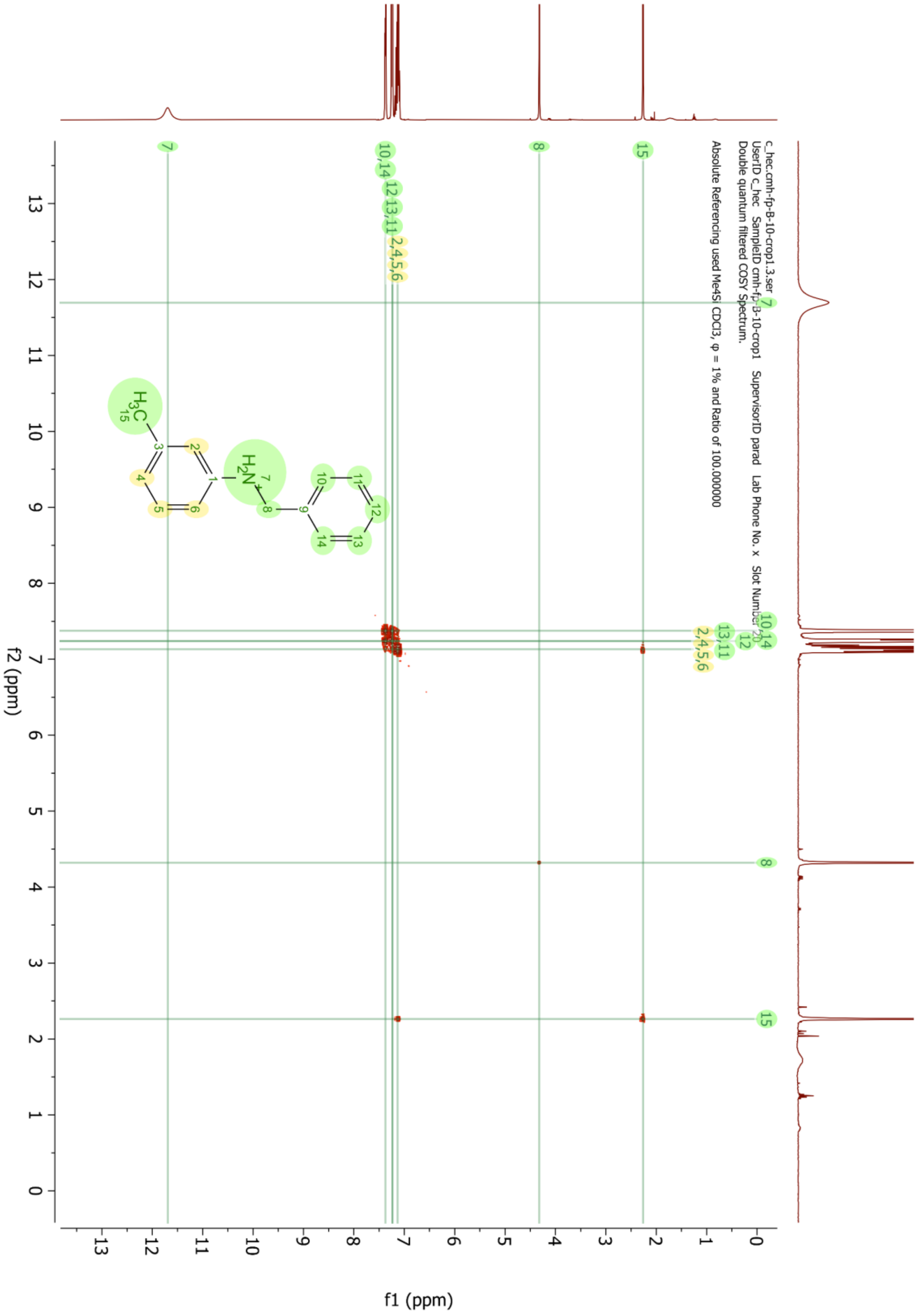

5.2.4 ^{1}H-^{13}C-HSQC-ME

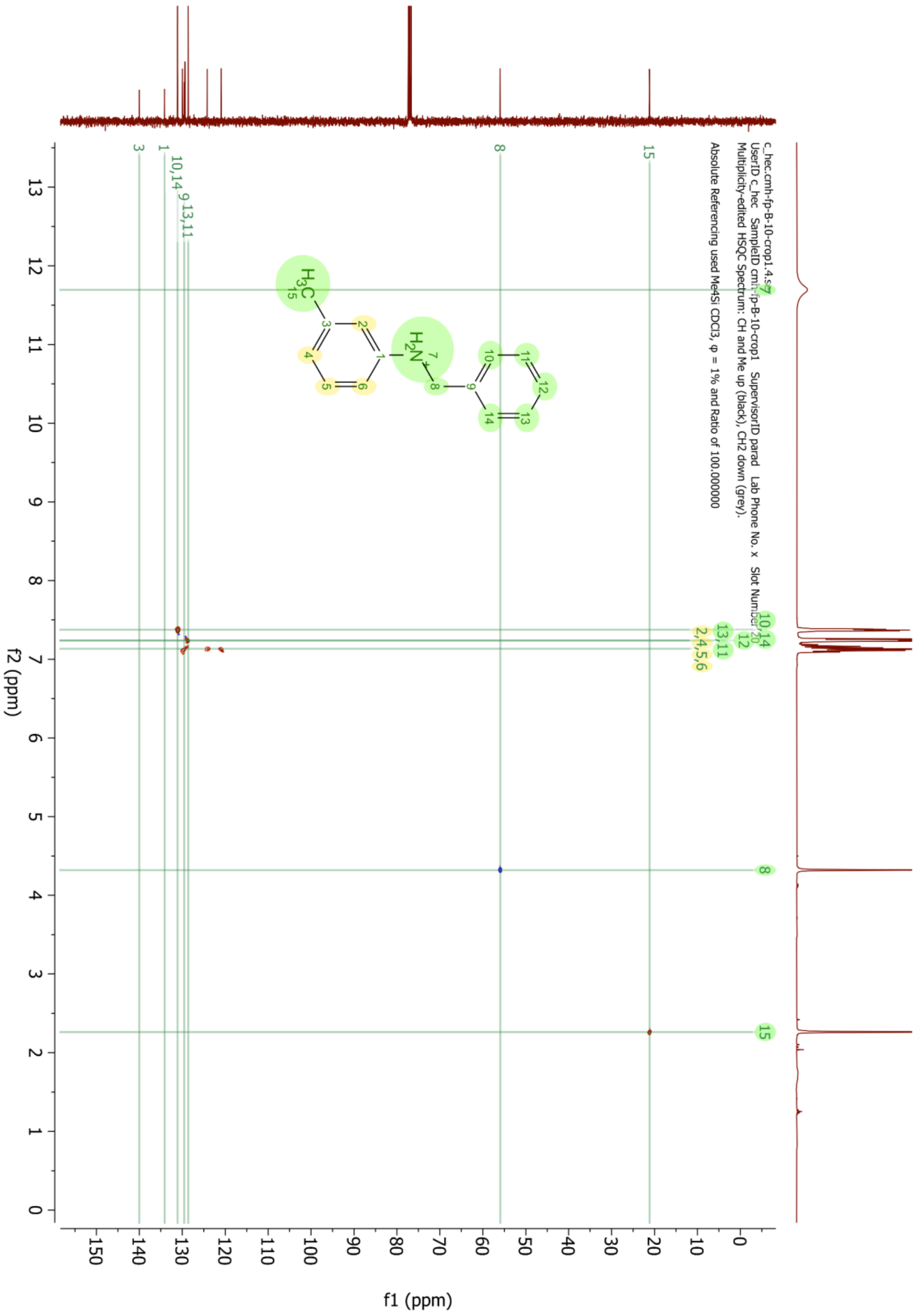

5.2.5 ^{1}H-^{13}C-HMBC

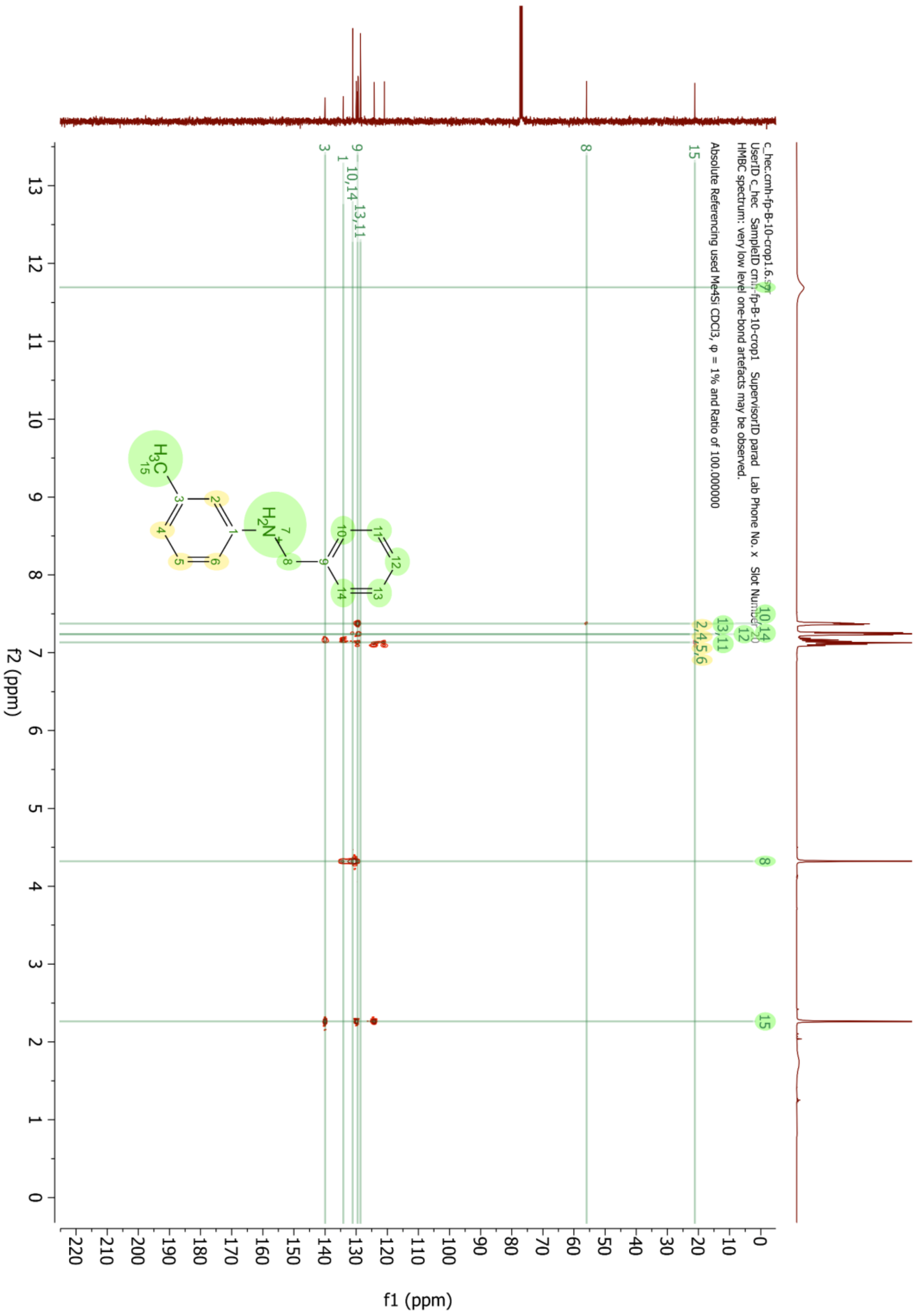

5.3 *3-isopropylaminotoluene*

5.3.1 *¹H-NMR*

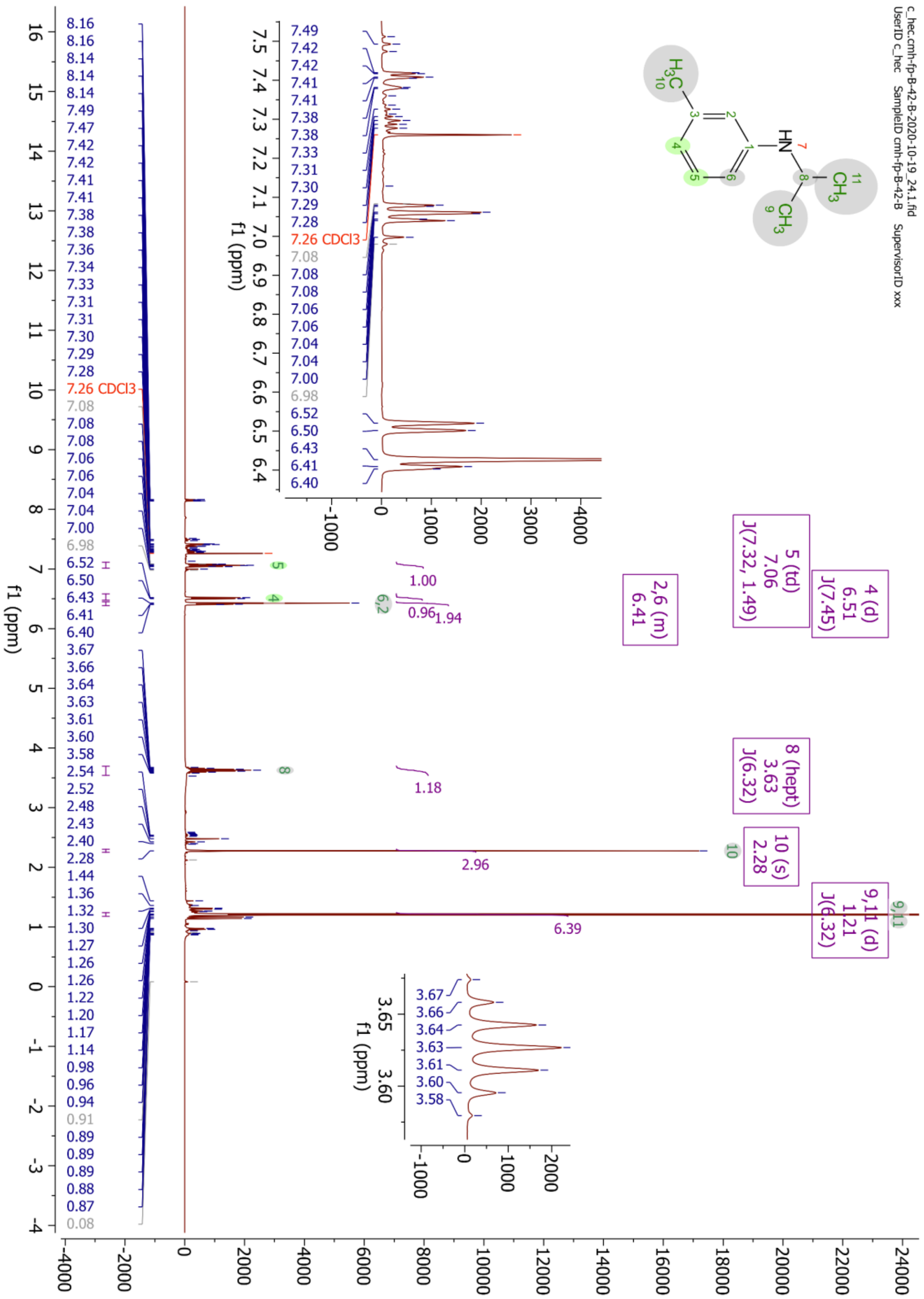

5.3.2 $^{13}C\text{-}\{^{1}H\}$-NMR

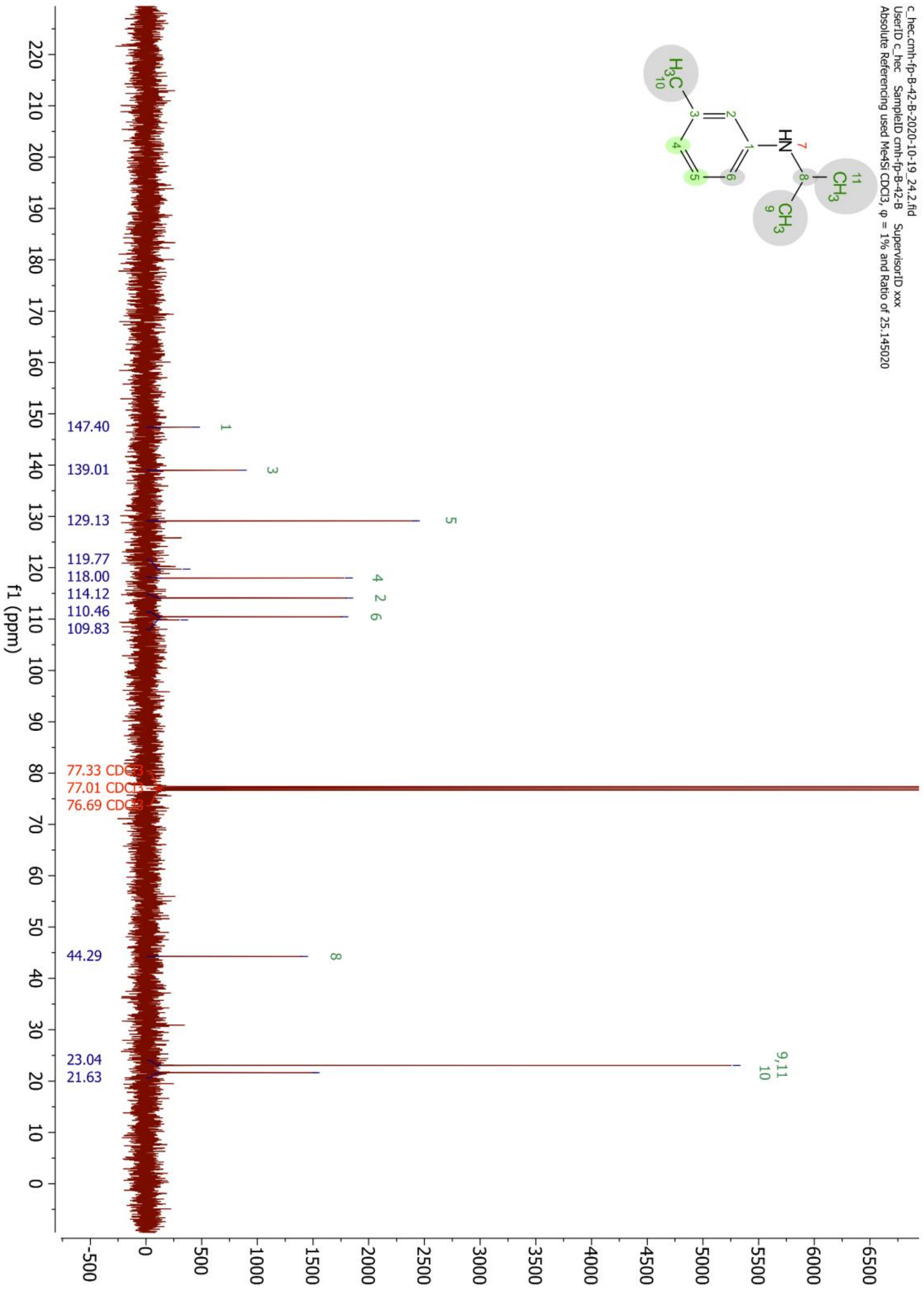

5.3.3 ^{1}H-^{1}H-COSY

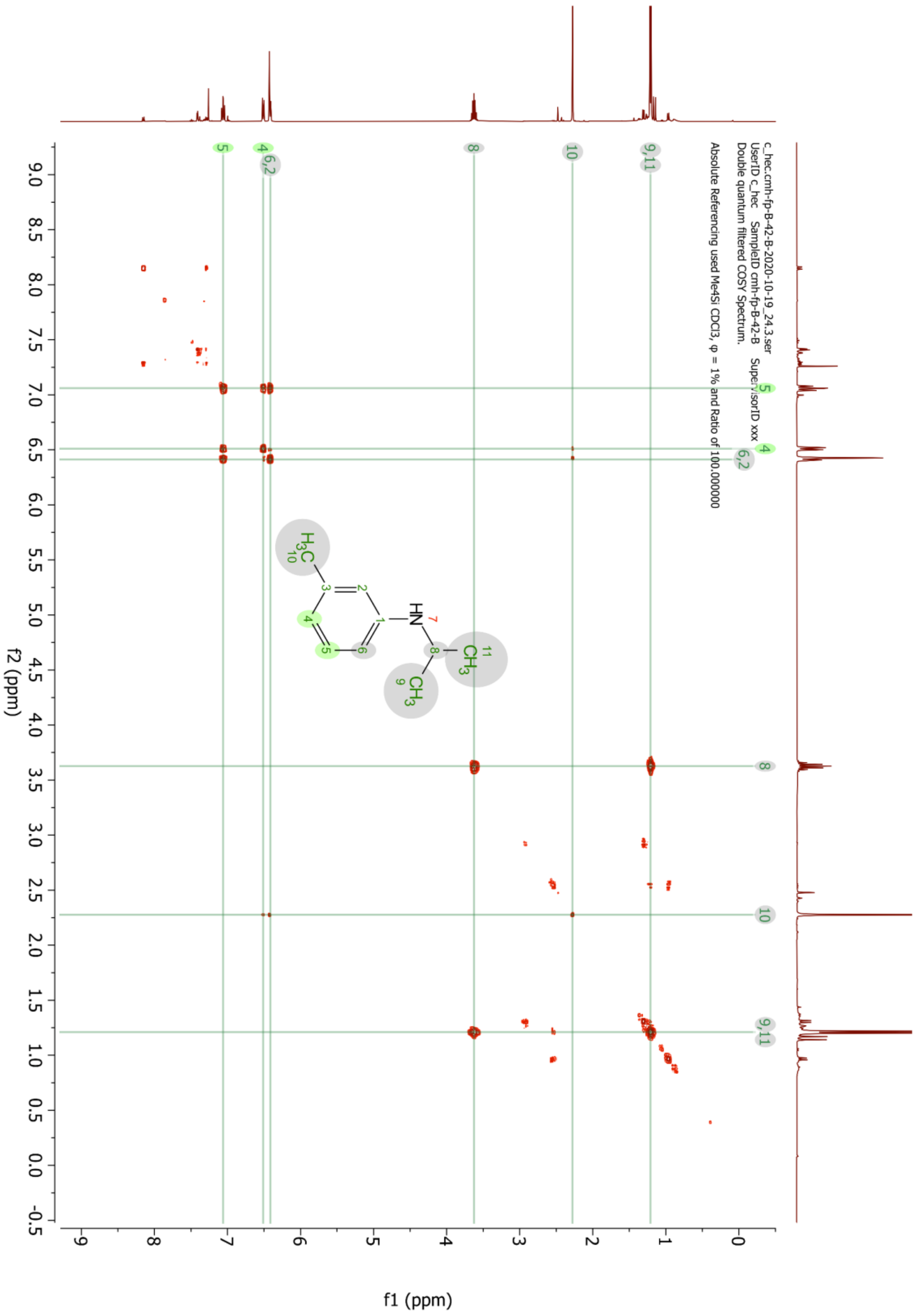

5.3.4 ^{1}H-^{13}C-HSQC-ME

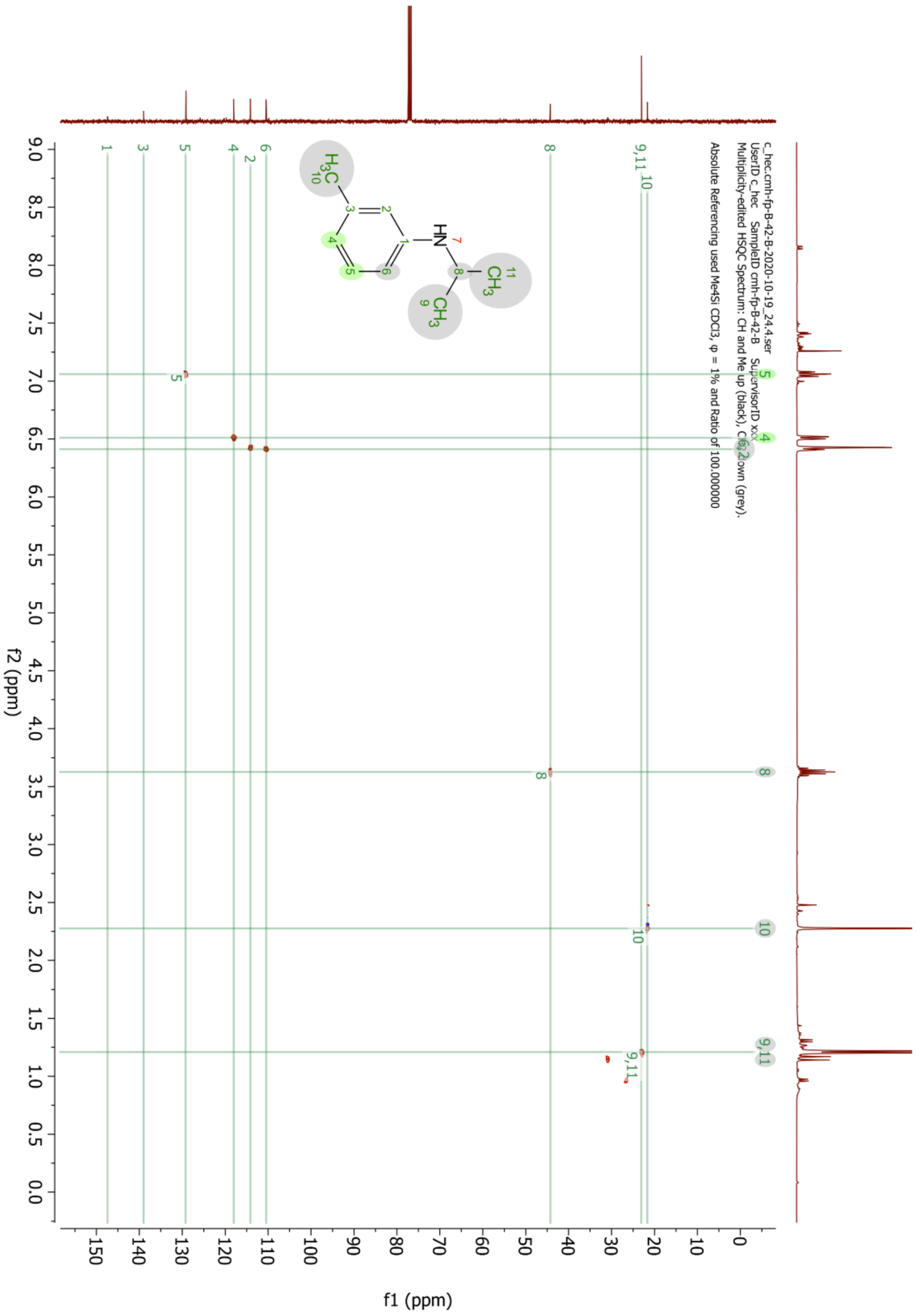

5.3.5 ^{1}H-^{13}C-HMBC

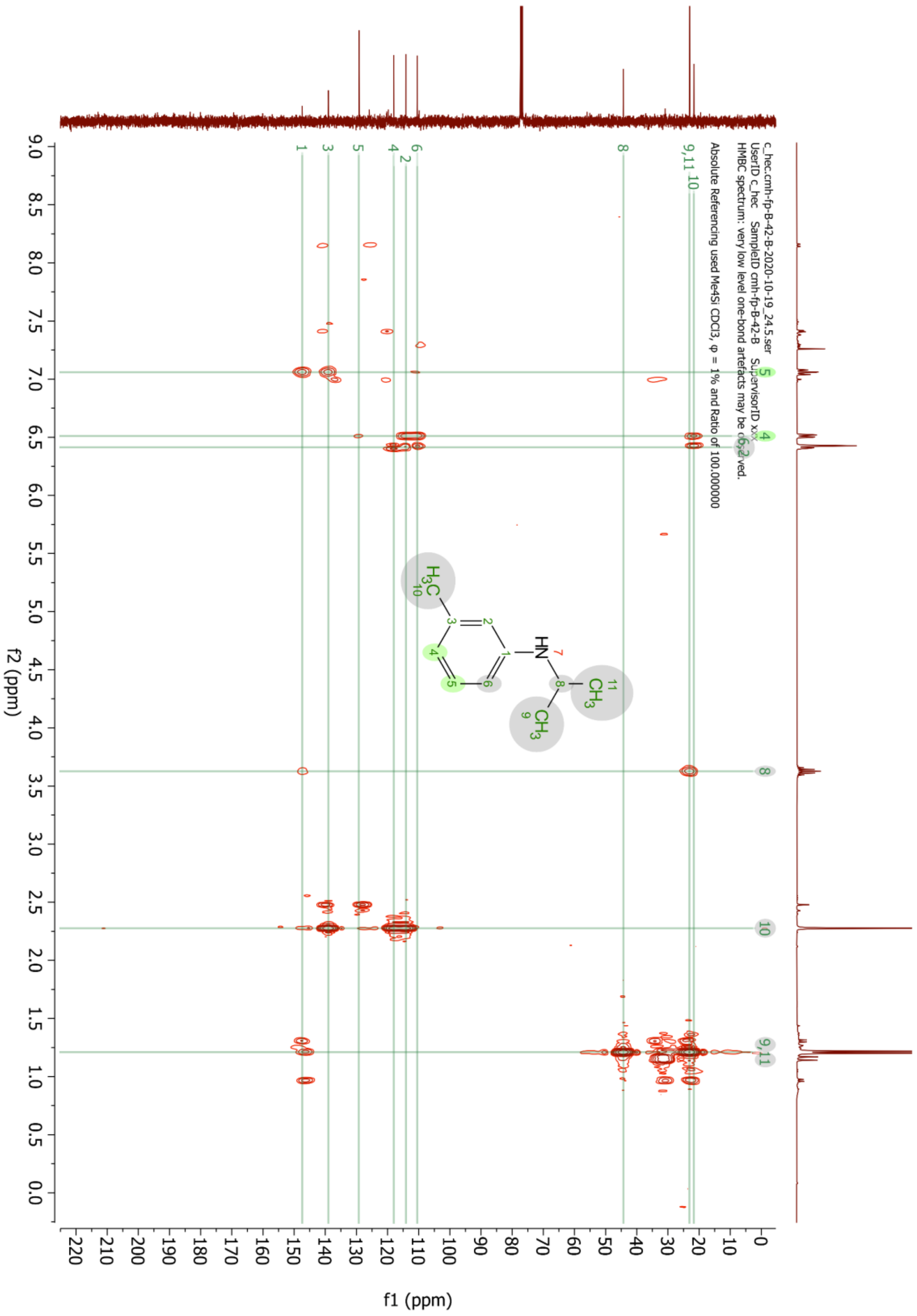

5.4 *3-(o-fluoro-α-methylbenzylamino)toluene* ***3b***

5.4.1 *^{1}H-NMR*

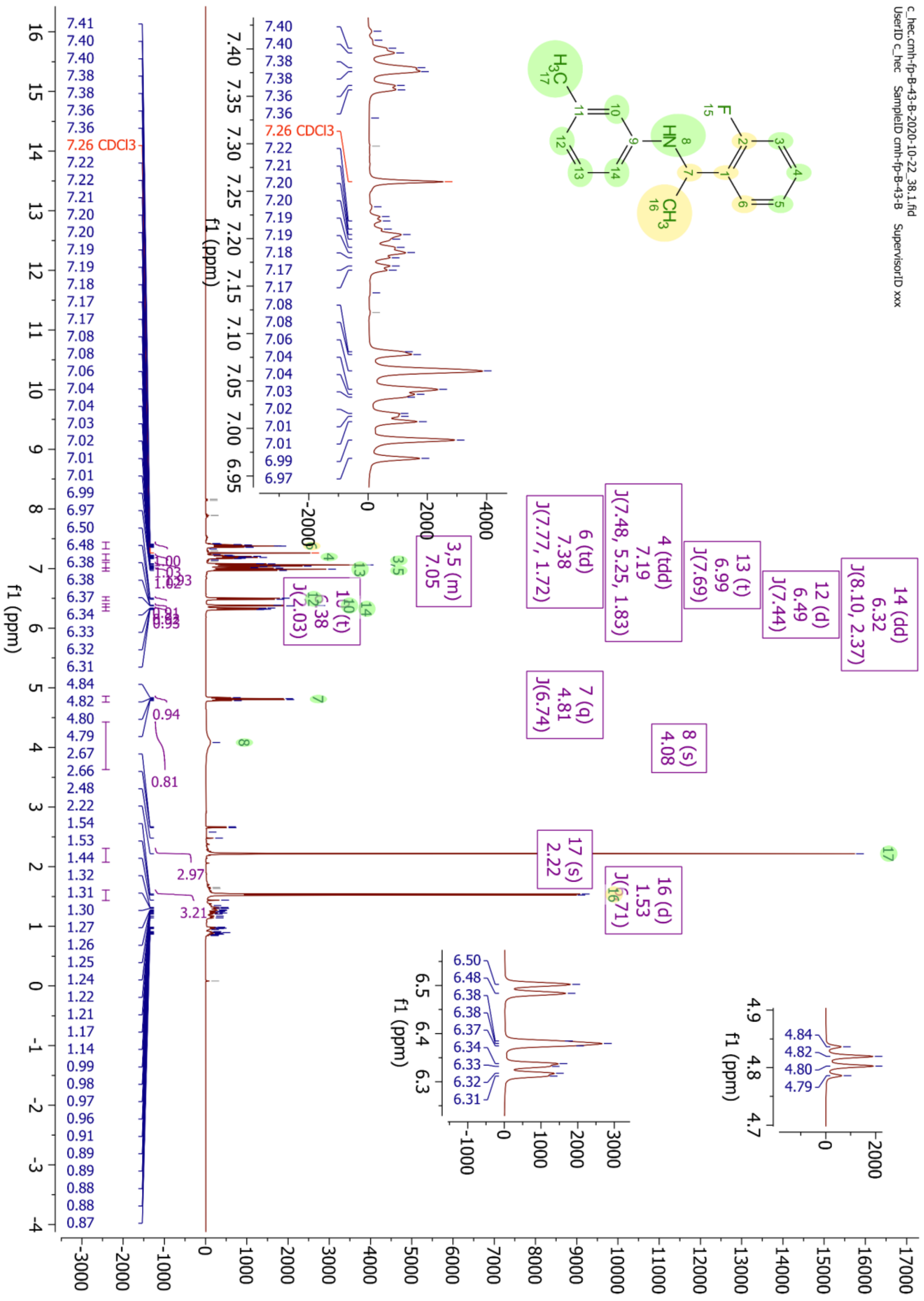

5.4.2 ^{13}C-{^{1}H}-NMR

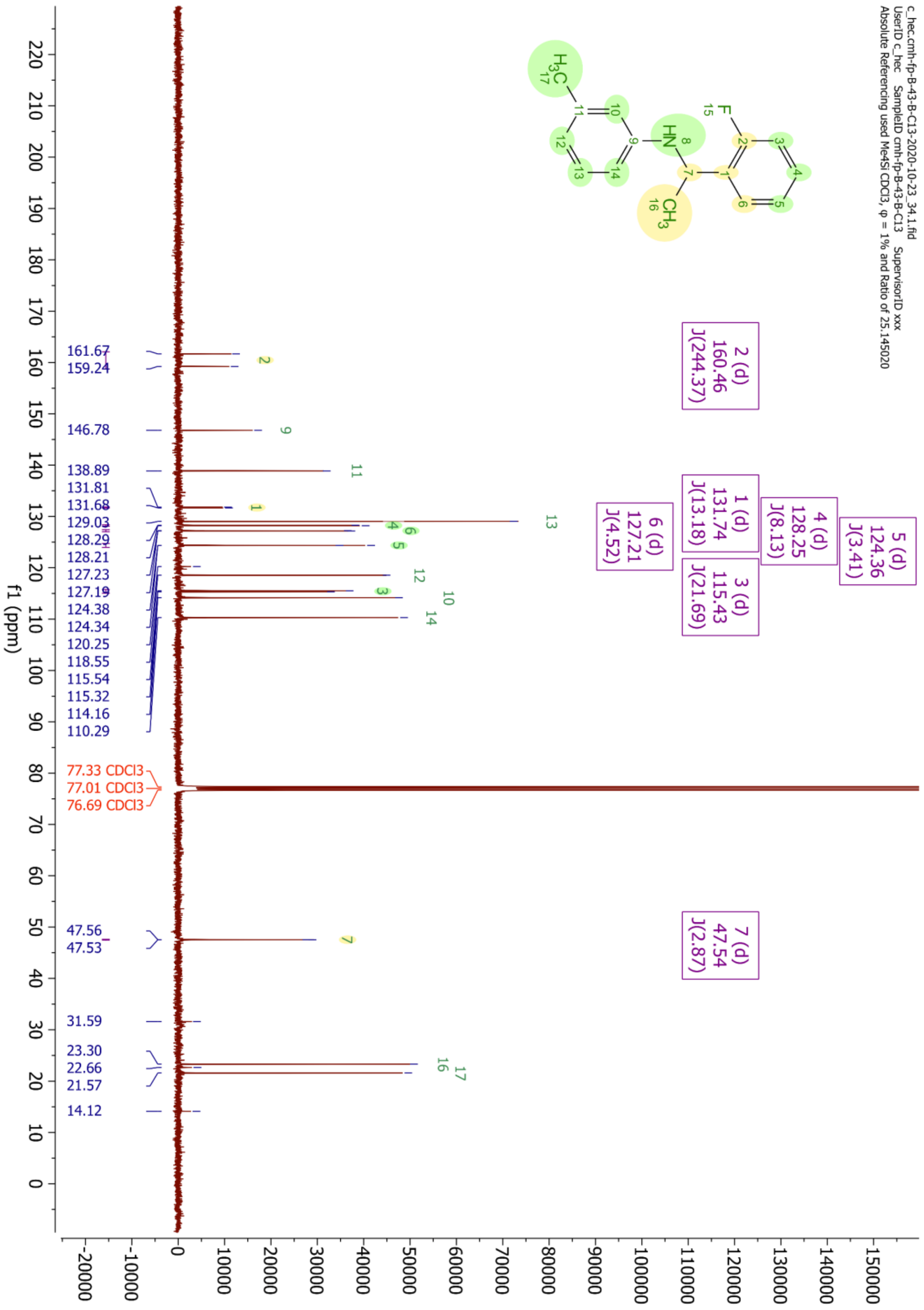

5.4.3 ^{19}F-NMR

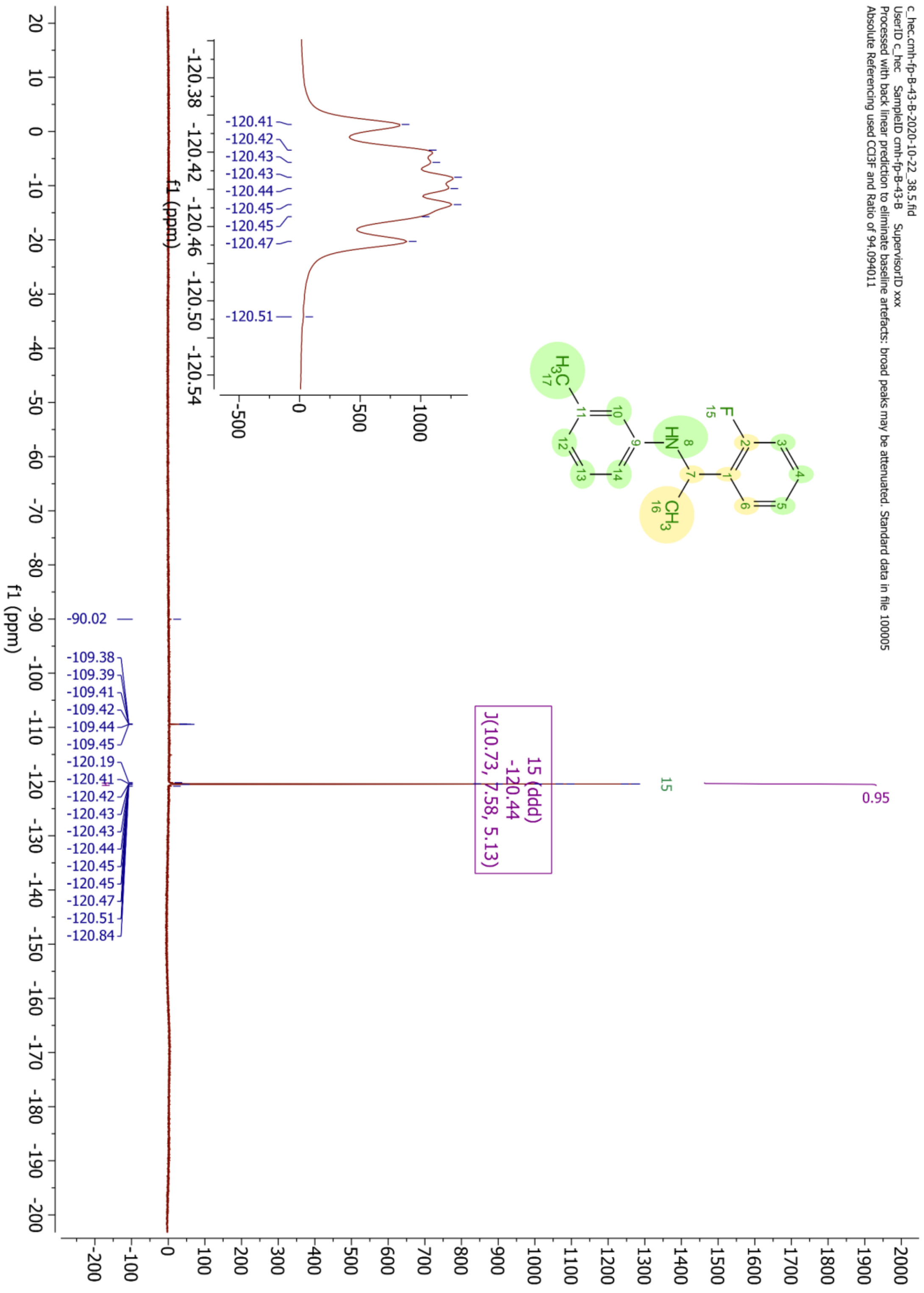

c_hec.cmh-fp-B-43-B-2020-10-22_38.5.fid
UserID c_hec SampleID cmh-fp-B-43-B SupervisorID xxx
Processed with back linear prediction to eliminate baseline artefacts: broad peaks may be attenuated. Standard data in file 100005
Absolute Referencing used CCl3F and Ratio of 94.094011
15 (ddd)
-120.44
J(10.73, 7.58, 5.13)
0.95
-90.02
-109.38
-109.39
-109.41
-109.42
-109.44
-109.45
-120.19
-120.41
-120.42
-120.43
-120.43
-120.44
-120.45
-120.45
-120.47
-120.51
-120.84
f1 (ppm)

5.4.4 ^{1}H-^{1}H-COSY

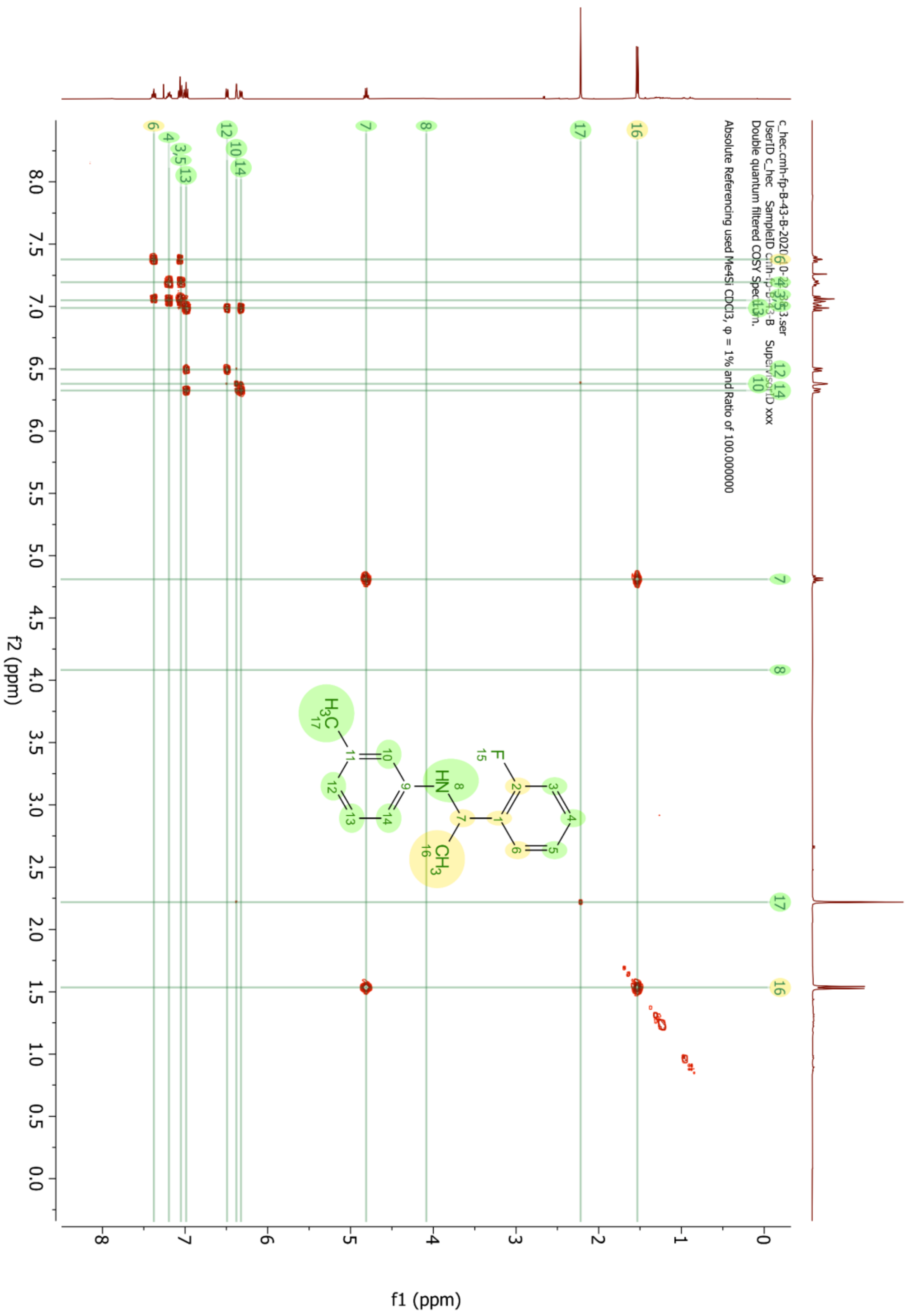

5.4.5 ^{1}H-^{13}C-HSQC-ME

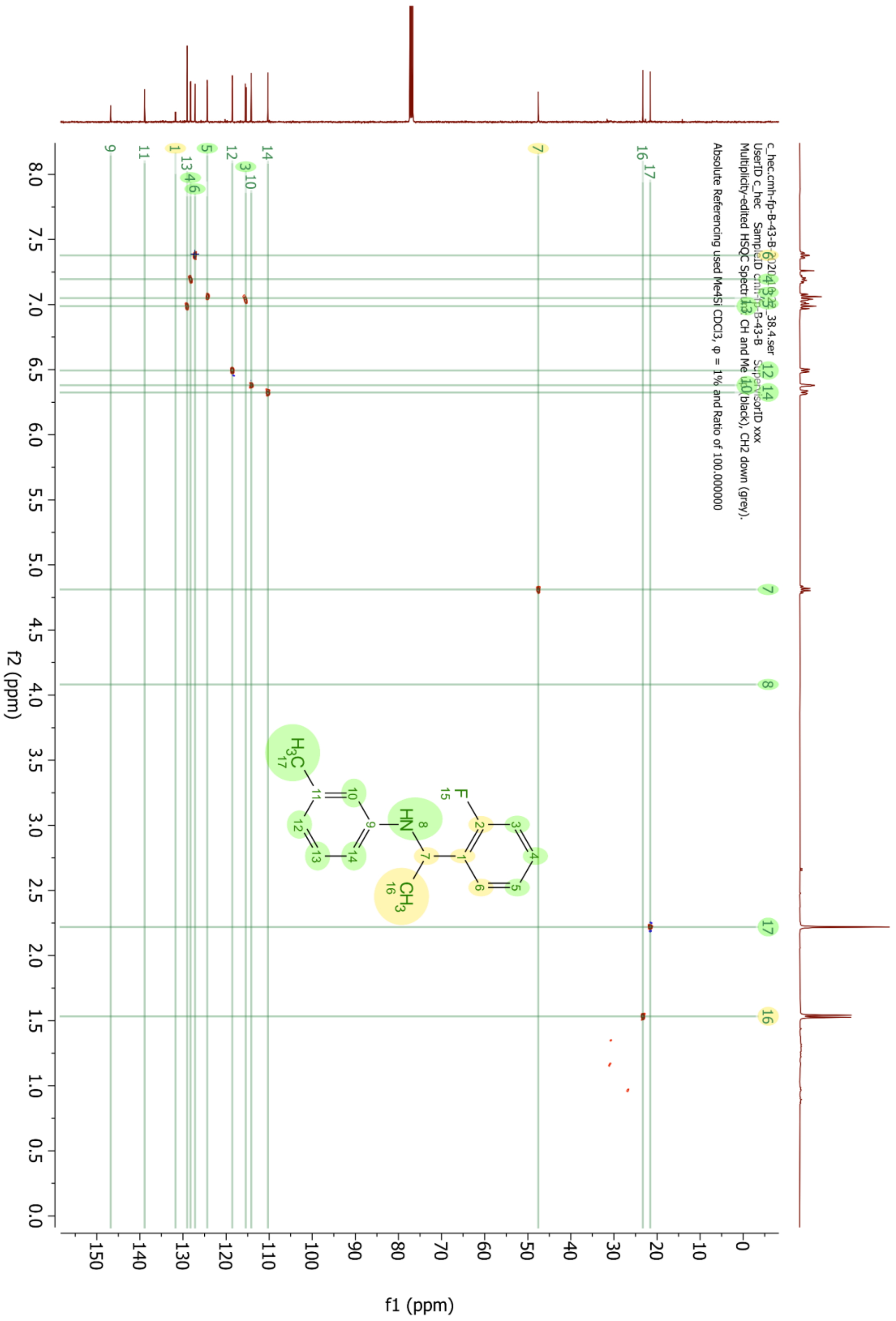

5.4.6 ^{1}H-^{13}C-HMBC

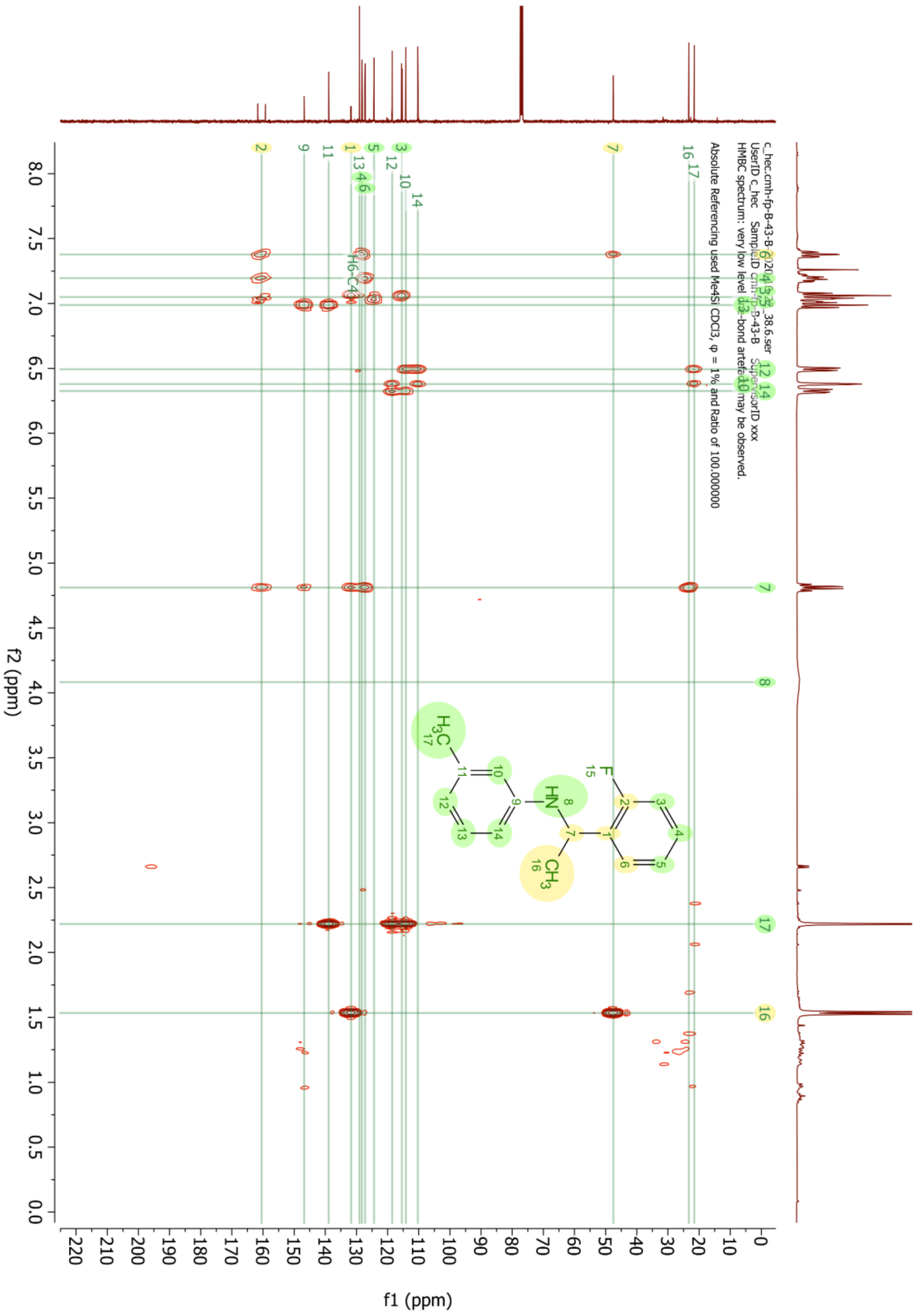

5.5 *(S)-3-(2-phenoxyisopropylamino)toluene (S)-**3c***

5.5.1 *^{1}H-NMR*

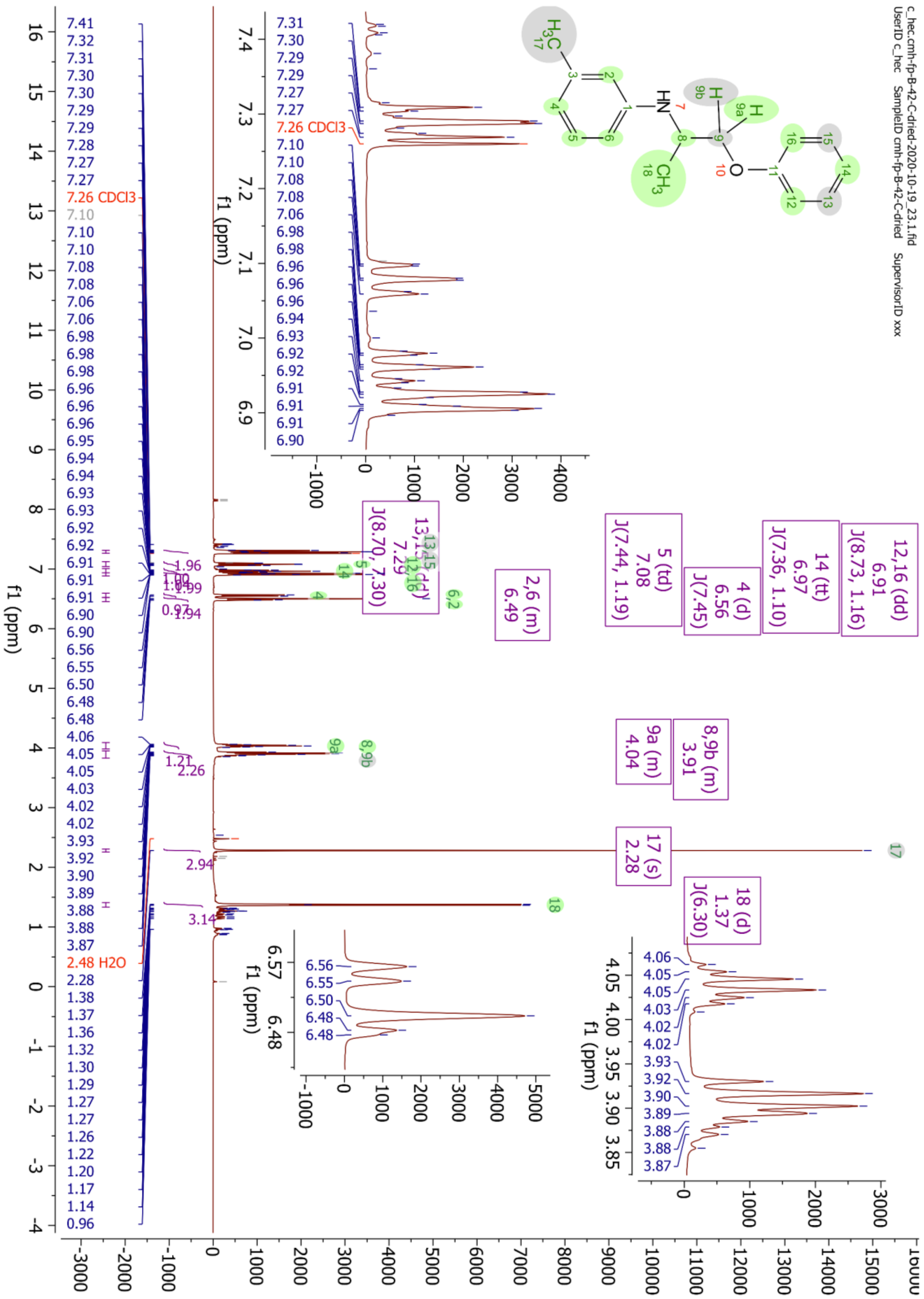

5.5.2 ^{13}C-{^{1}H}-NMR

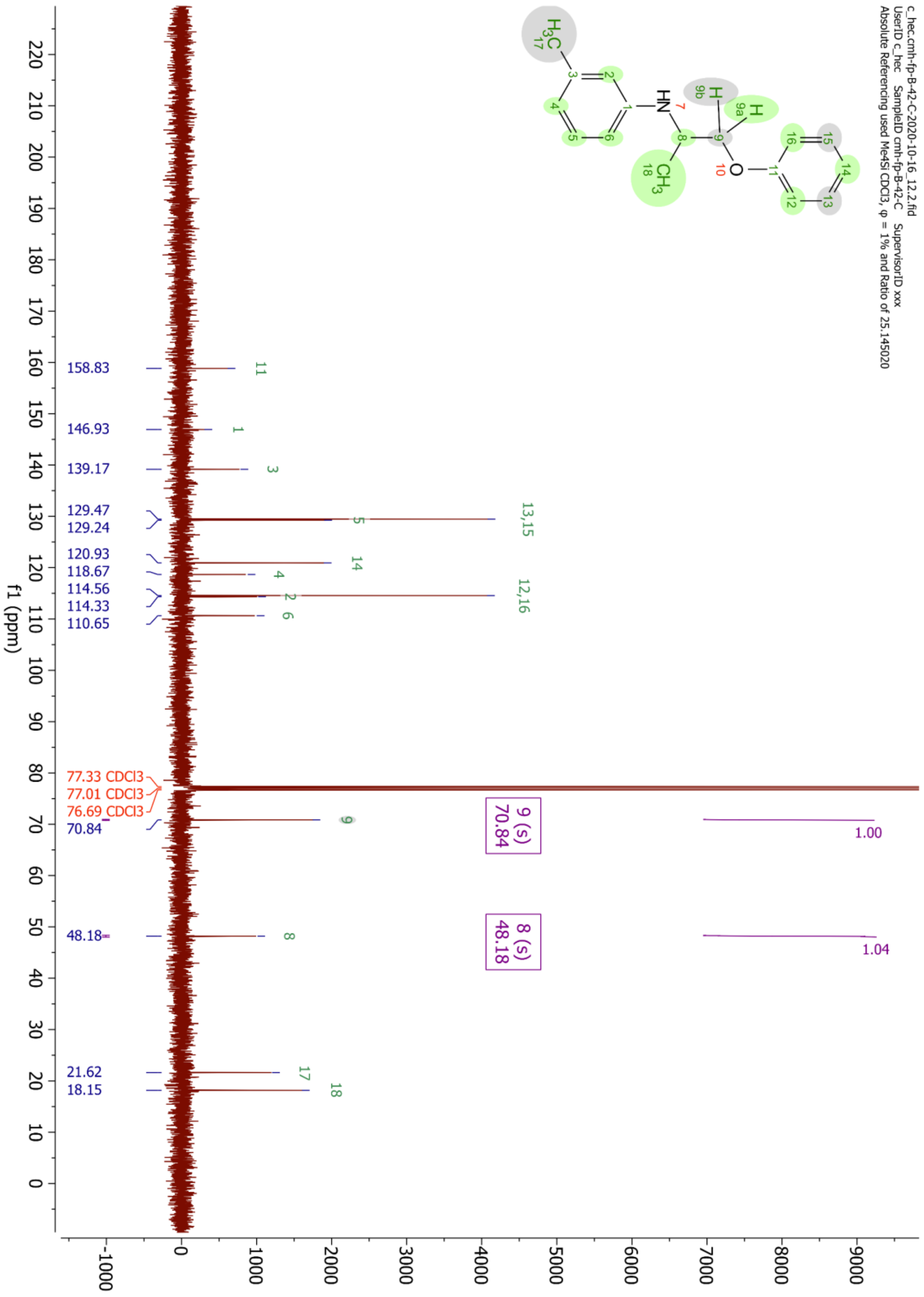

5.5.3 ^{1}H-^{1}H-COSY

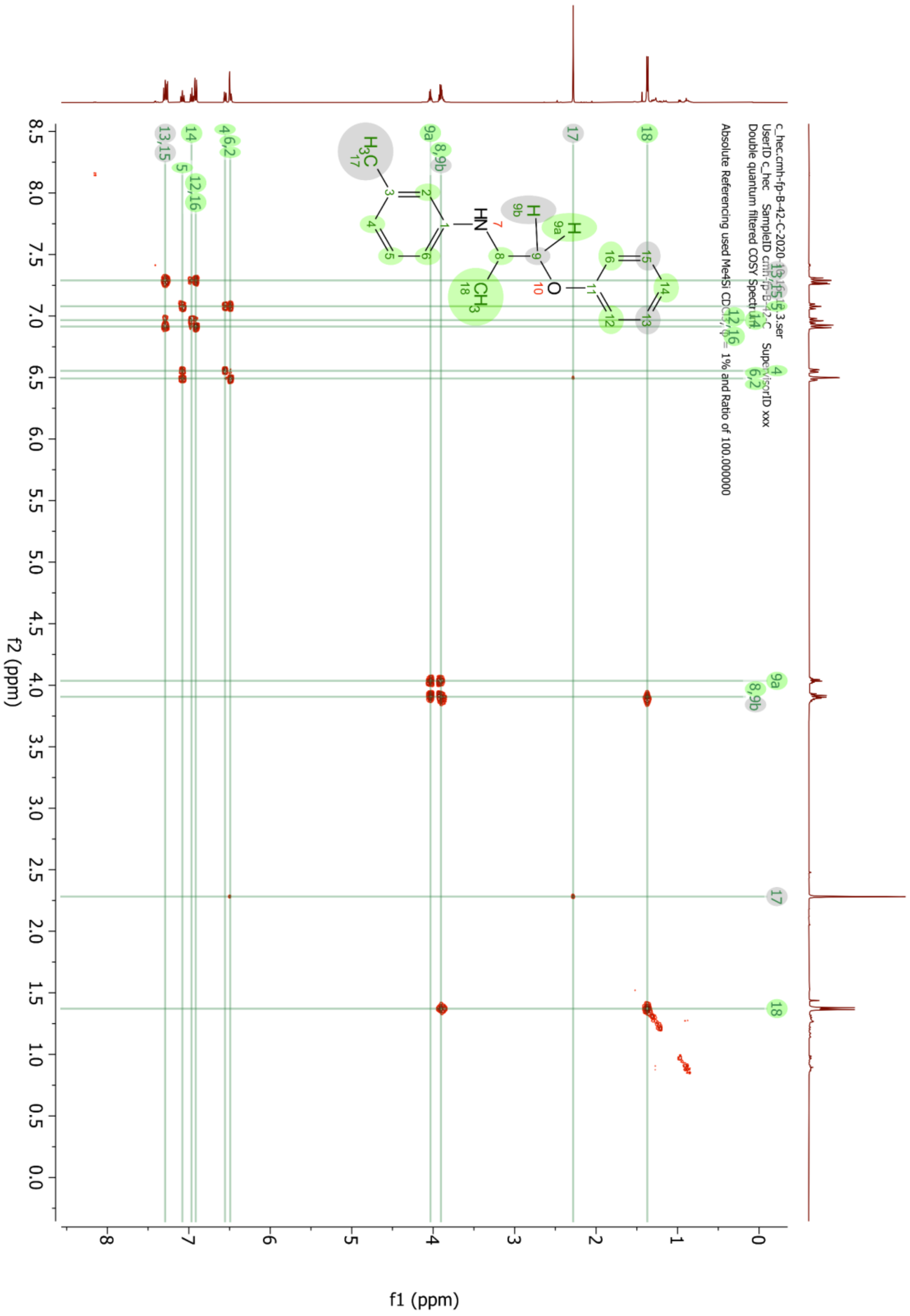

5.5.4 ^{1}H-^{13}C-HSQC-ME

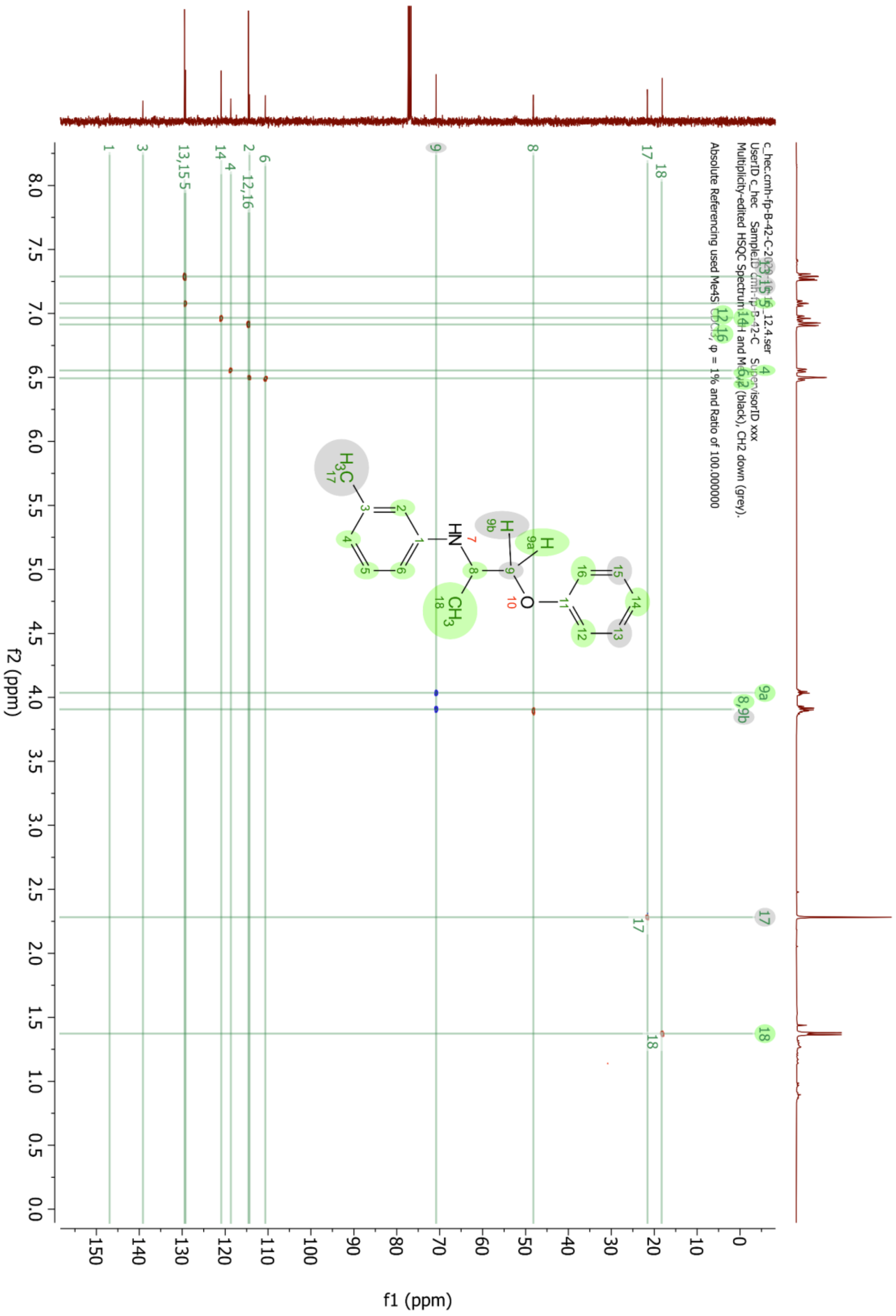

5.5.5 ^{1}H-^{13}C-HMBC

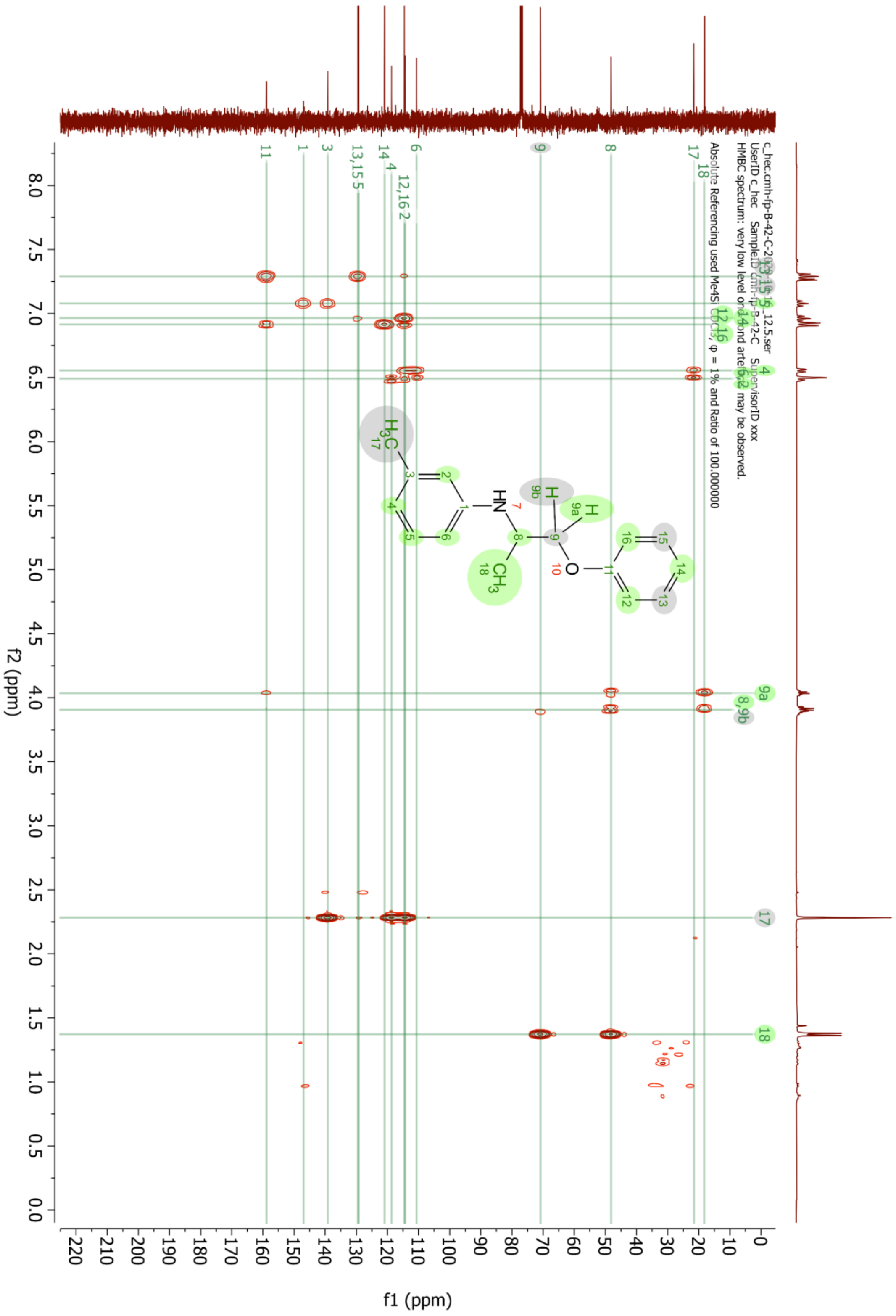

5.6 *3-(hexan-2-amino)toluene **3d***

5.6.1 *^{1}H-NMR*

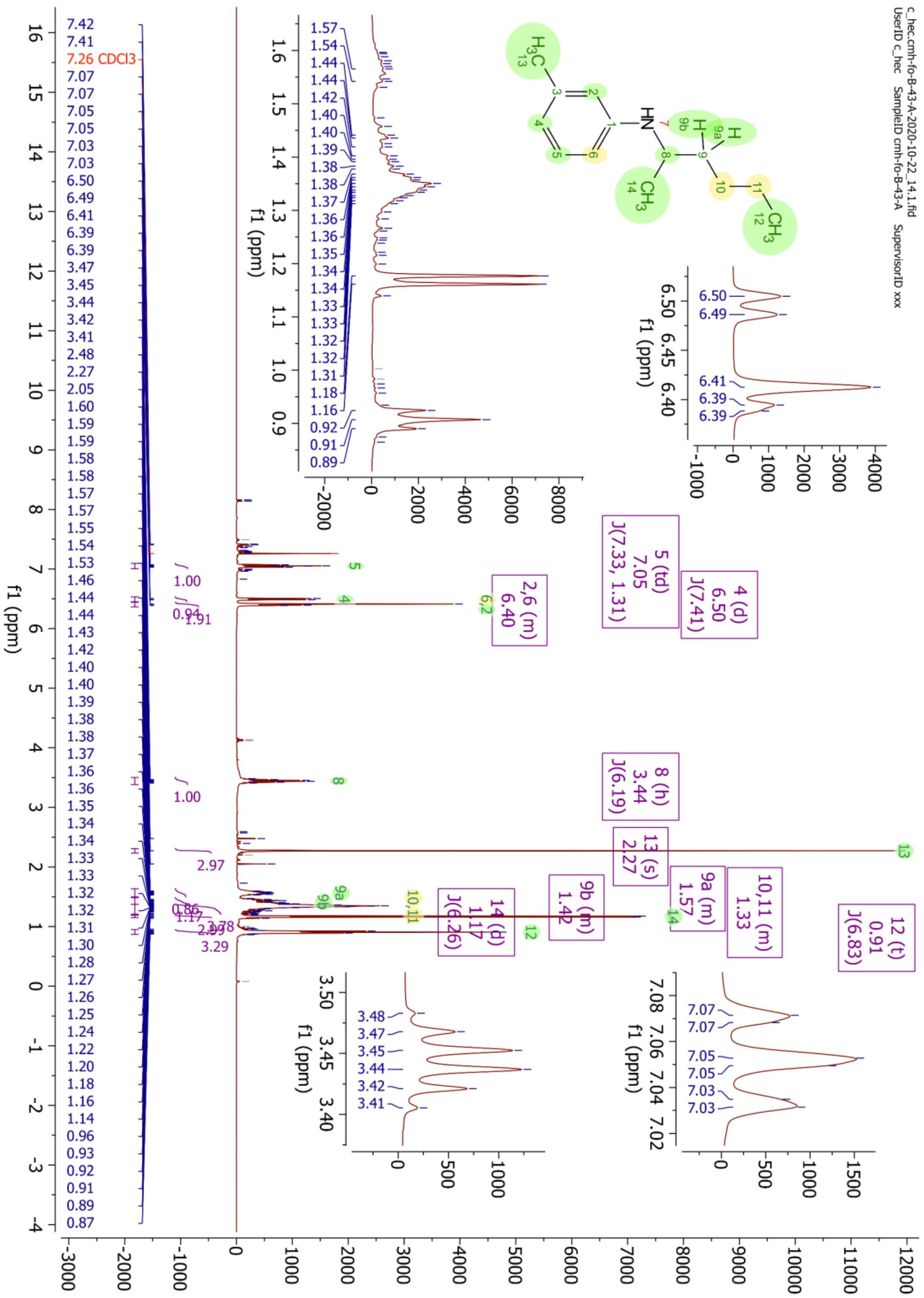

5.6.2 ^{13}C-{^{1}H}-NMR

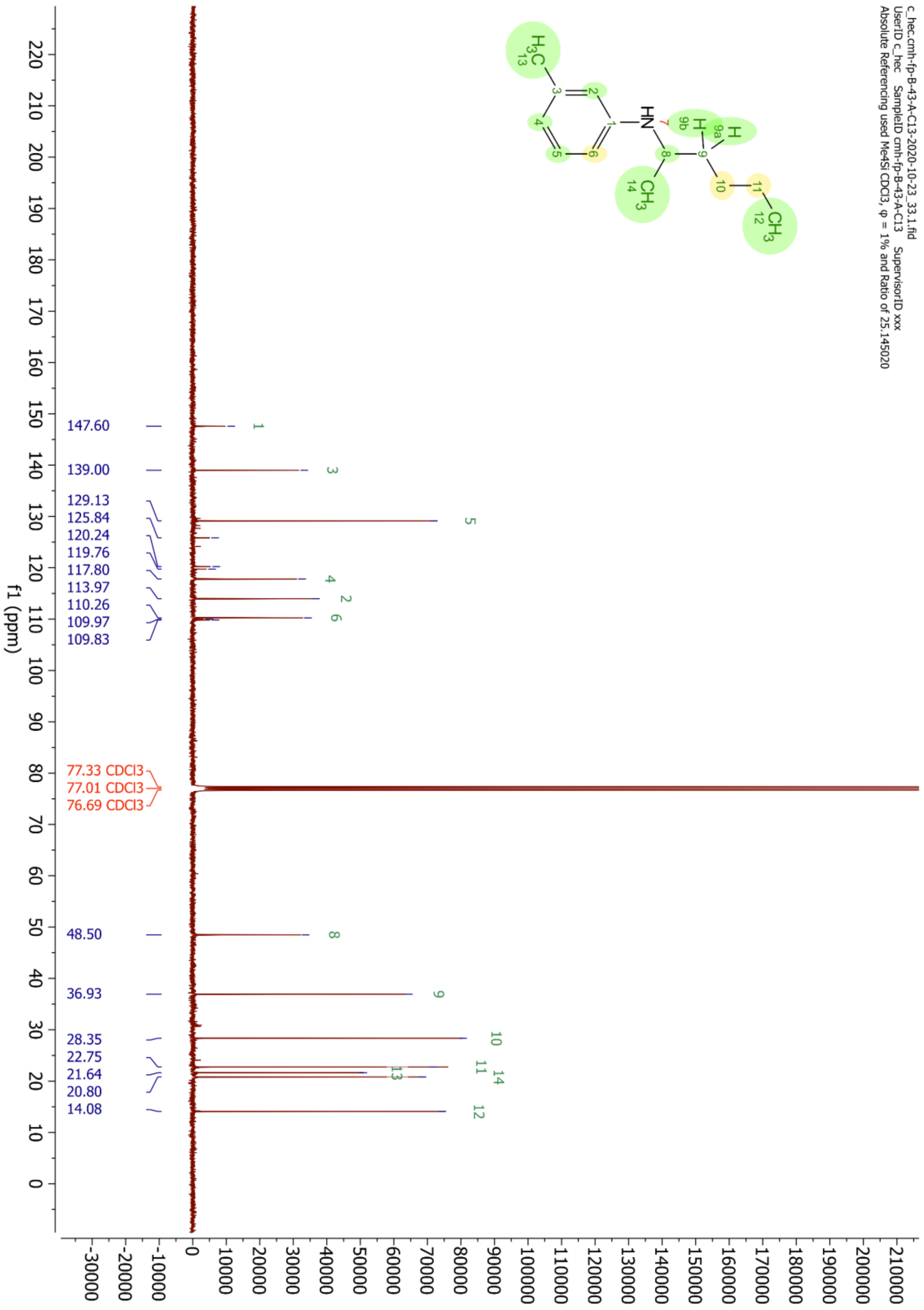

5.6.3 ^{1}H-^{1}H-COSY

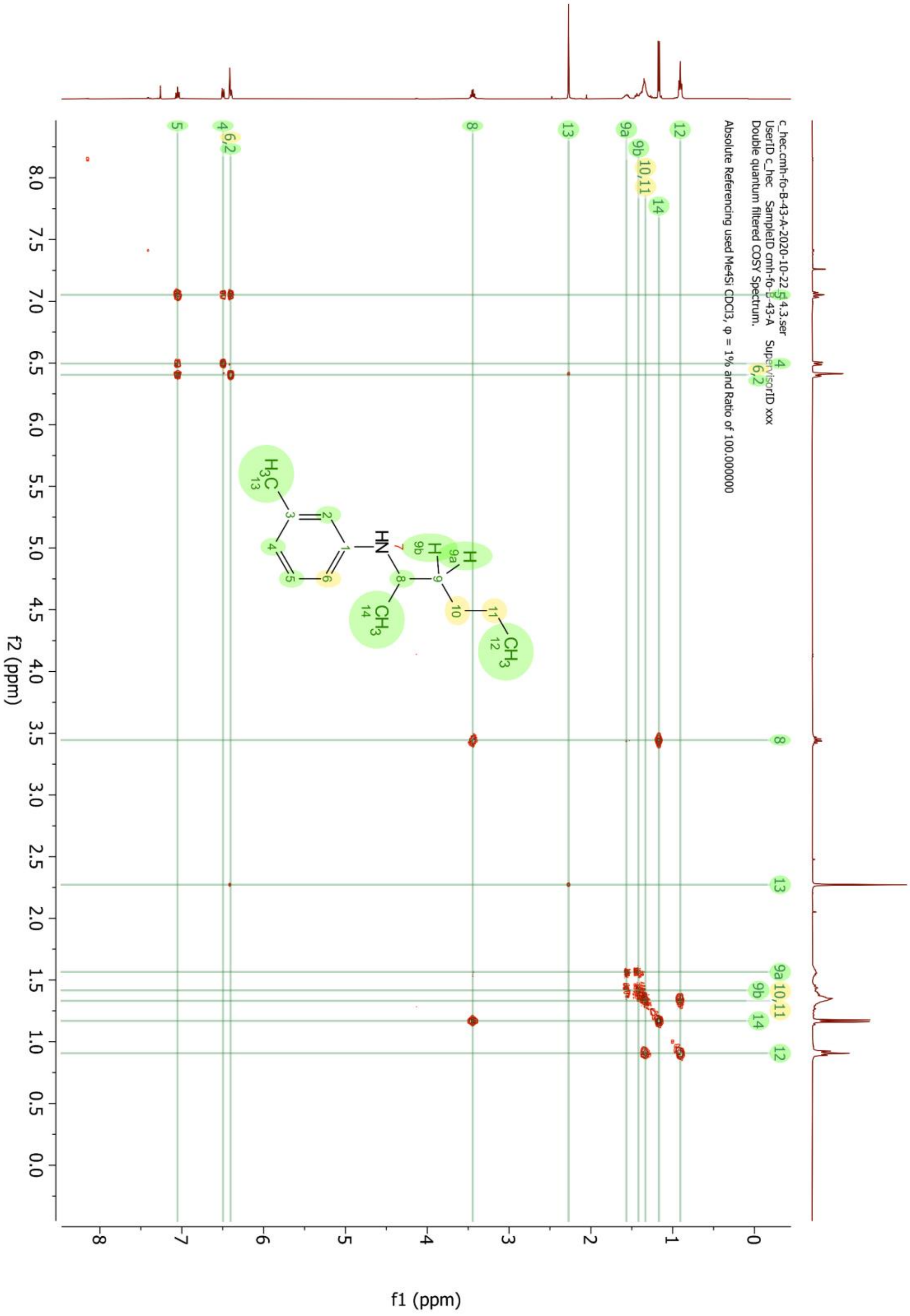

5.6.4 ^{1}H-^{13}C-HSQC-ME

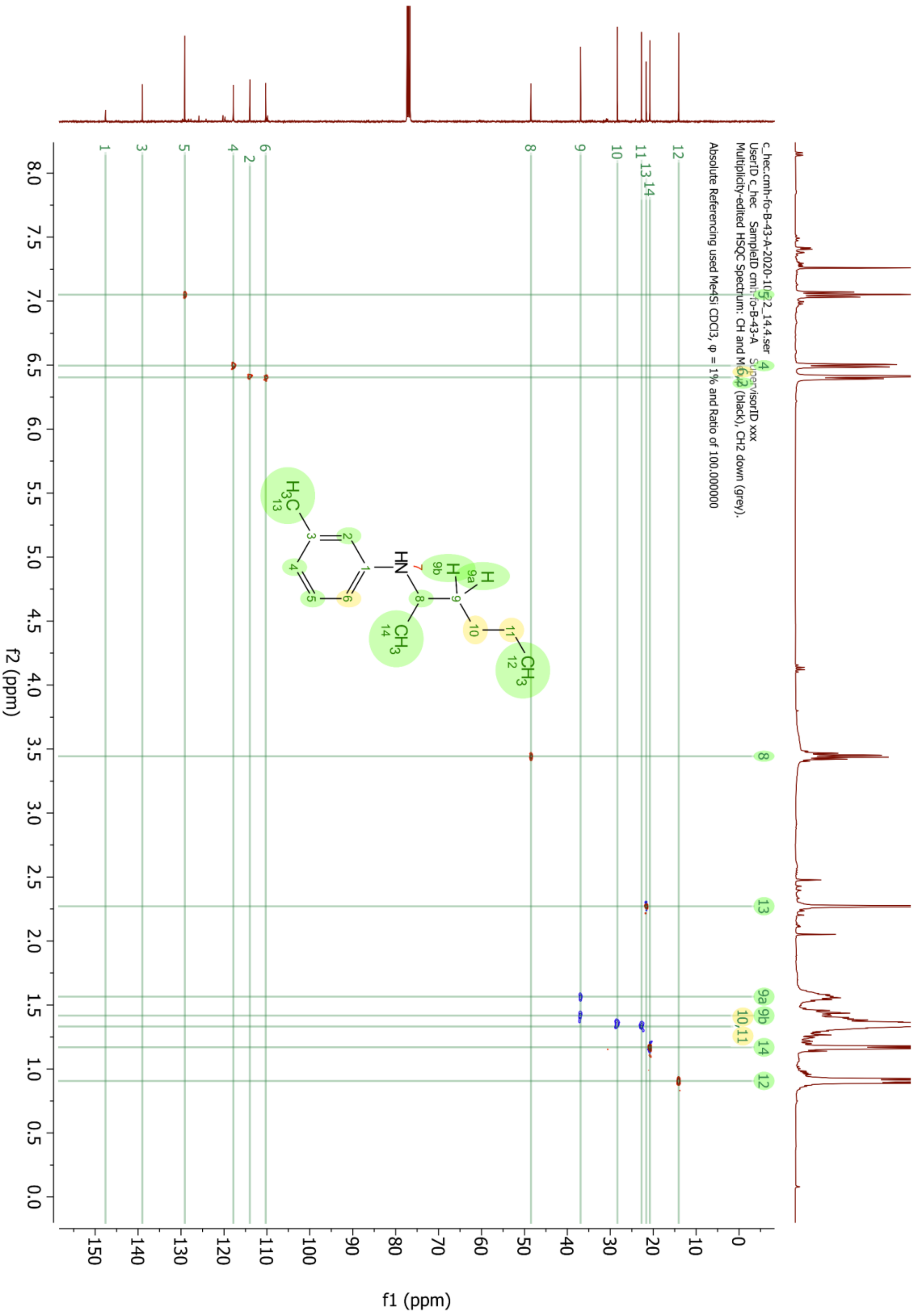

5.6.5 ¹H-¹³C-HMBC

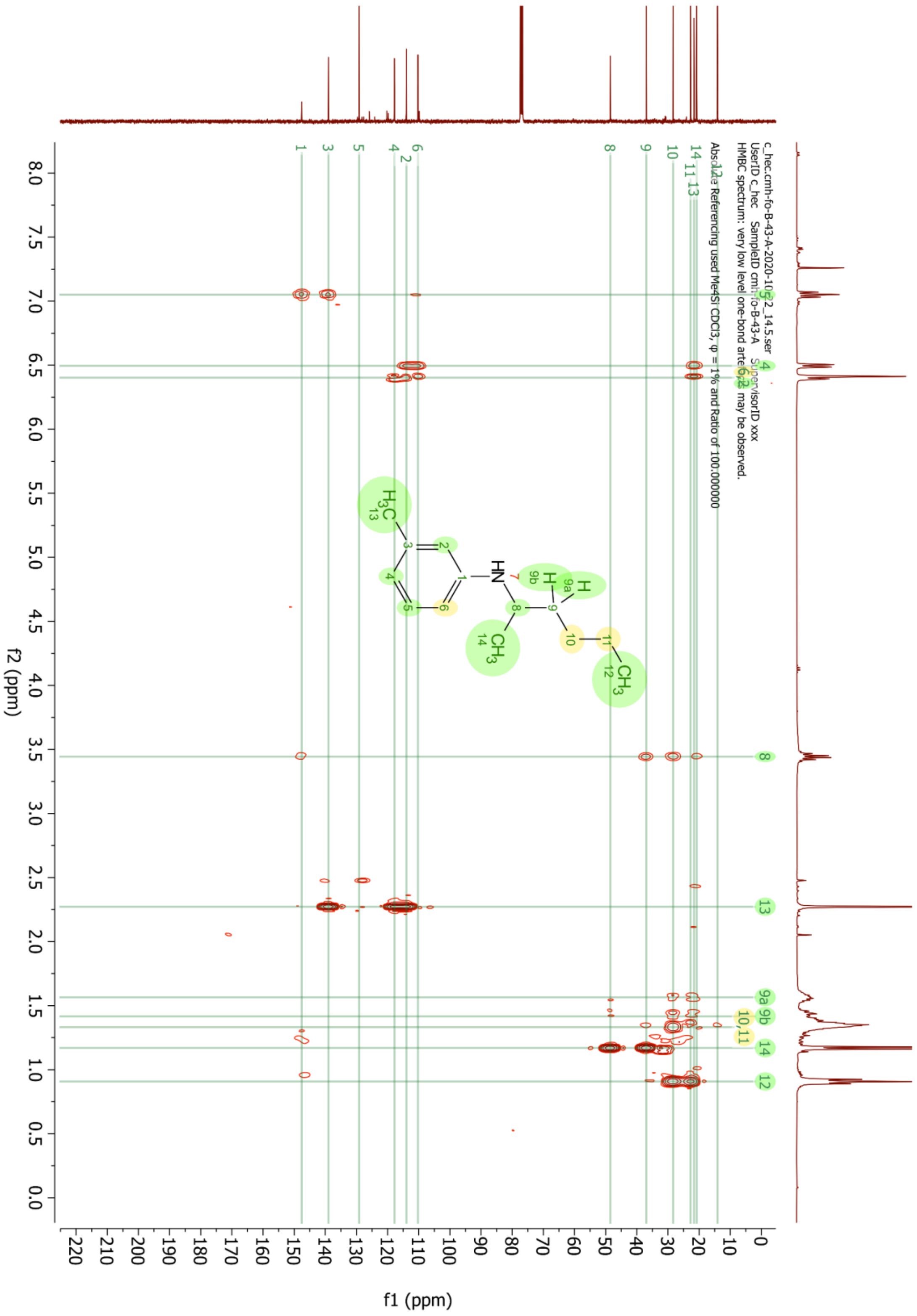

5.7 *3-(1-(thiazol-2-yl)ethylamino)toluene **3e***

5.7.1 *¹H-NMR*

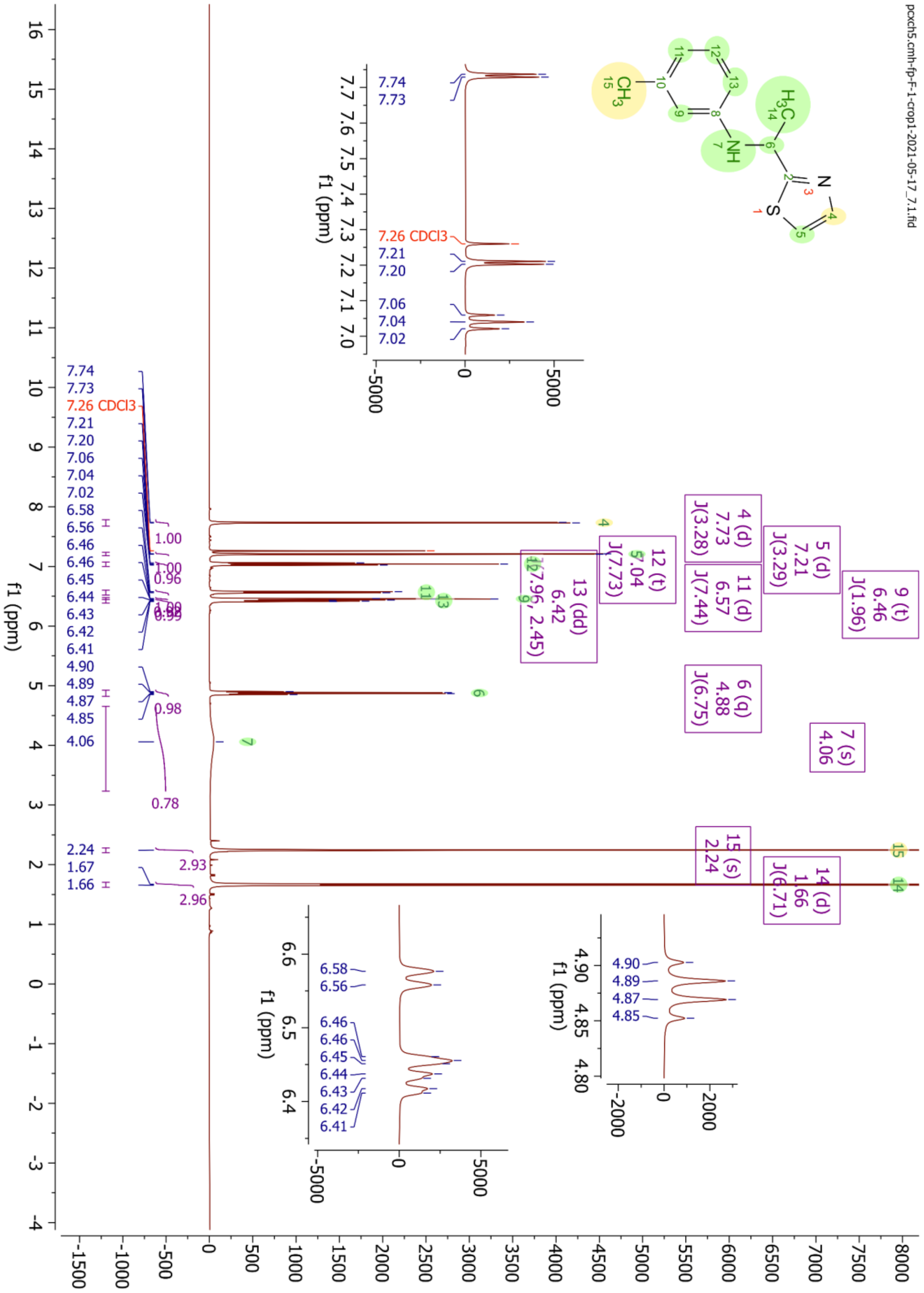

5.7.2 ^{13}C-{^{1}H}-NMR

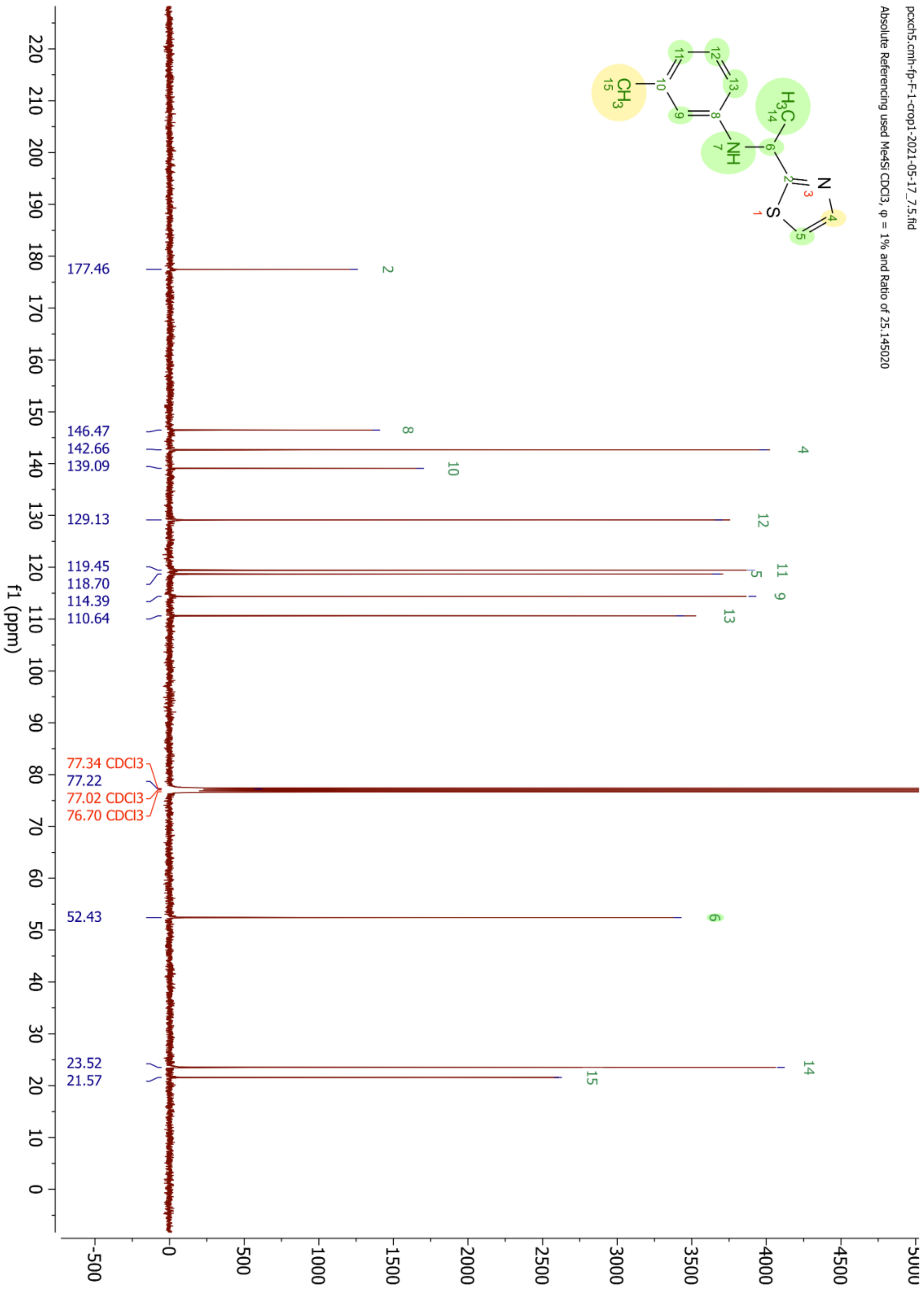

5.7.3 ^1H-^1H-COSY

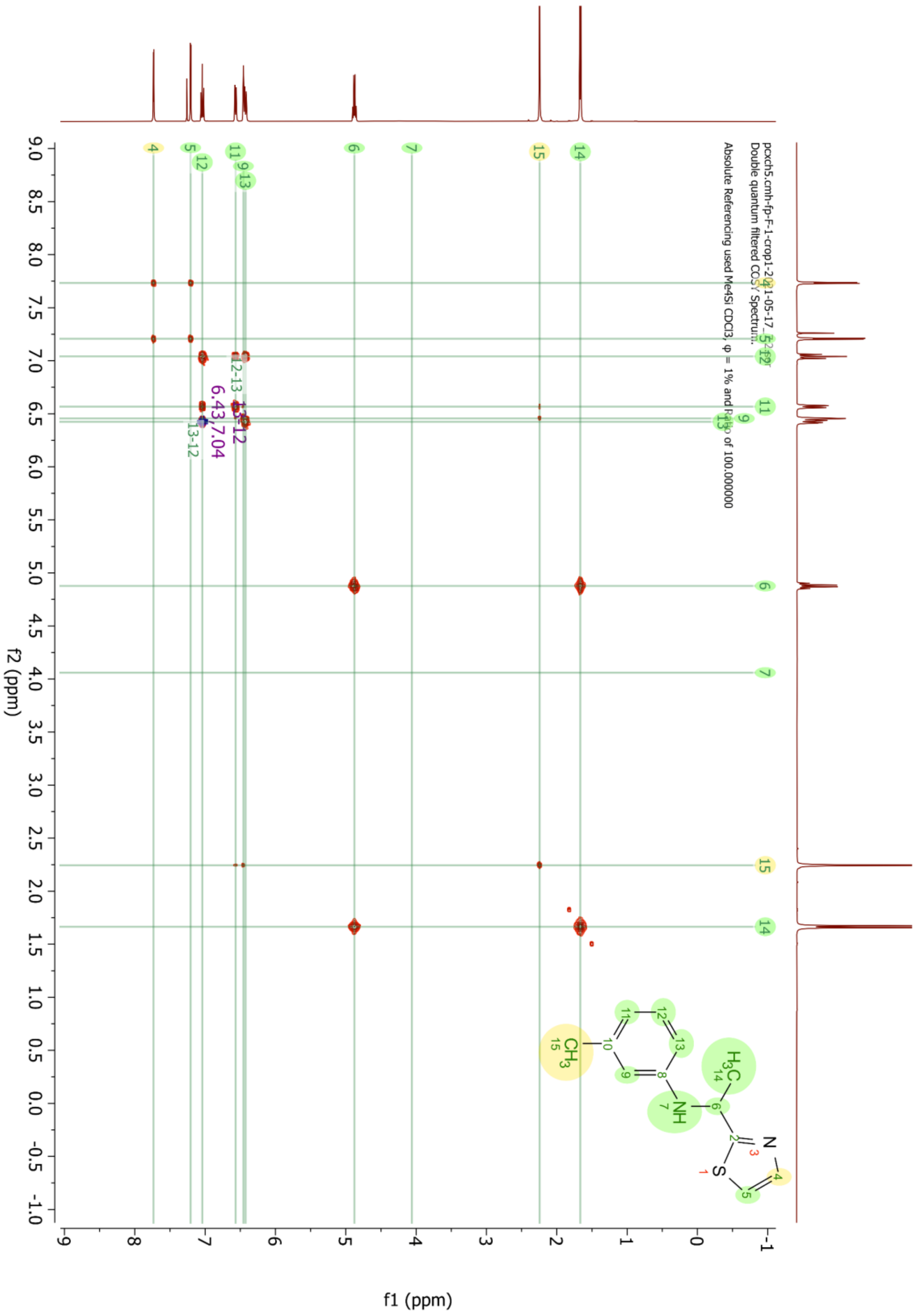

5.7.4 ^{1}H-^{13}C-HSQC-ME

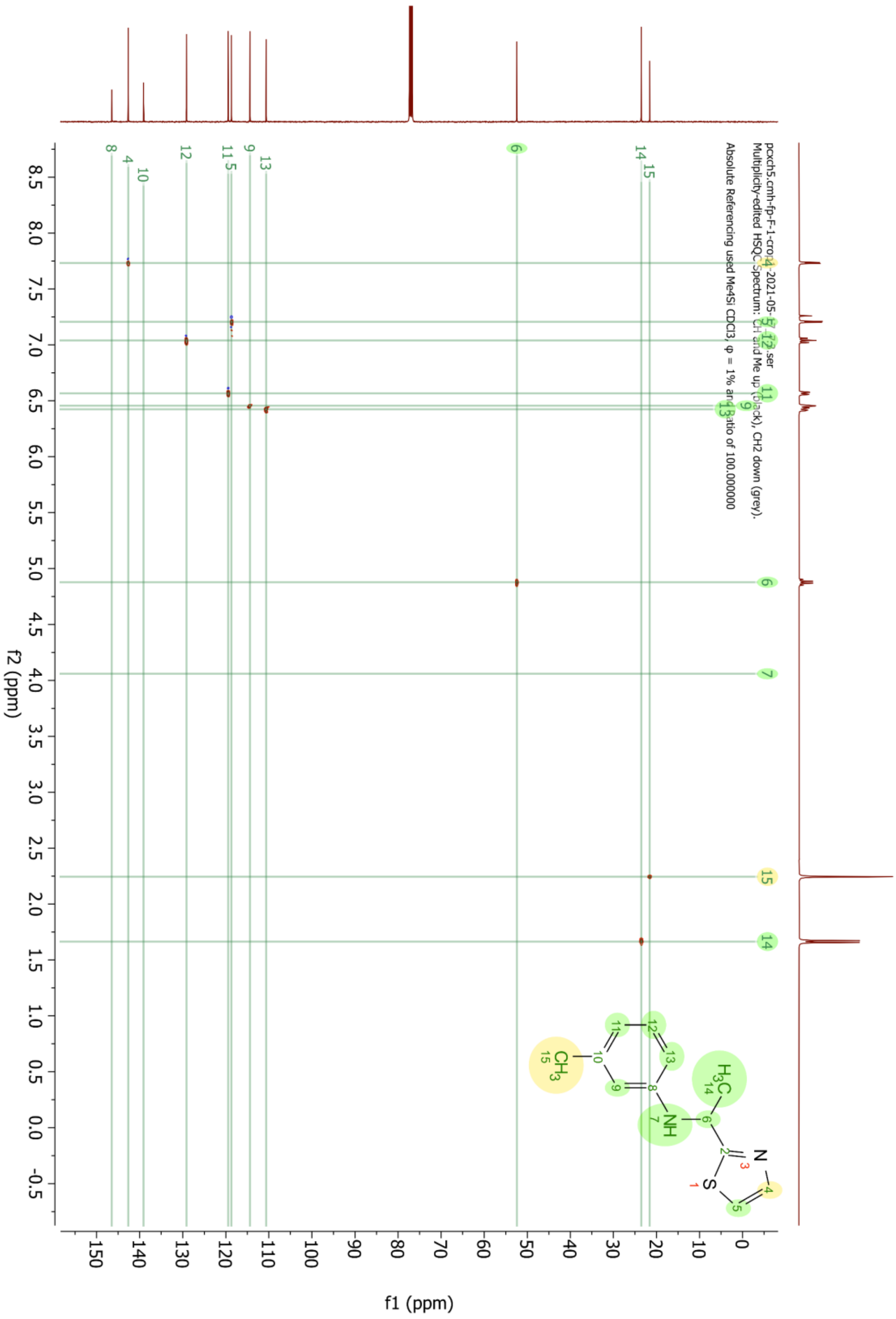

5.7.5 ^{1}H-^{13}C-HMBC

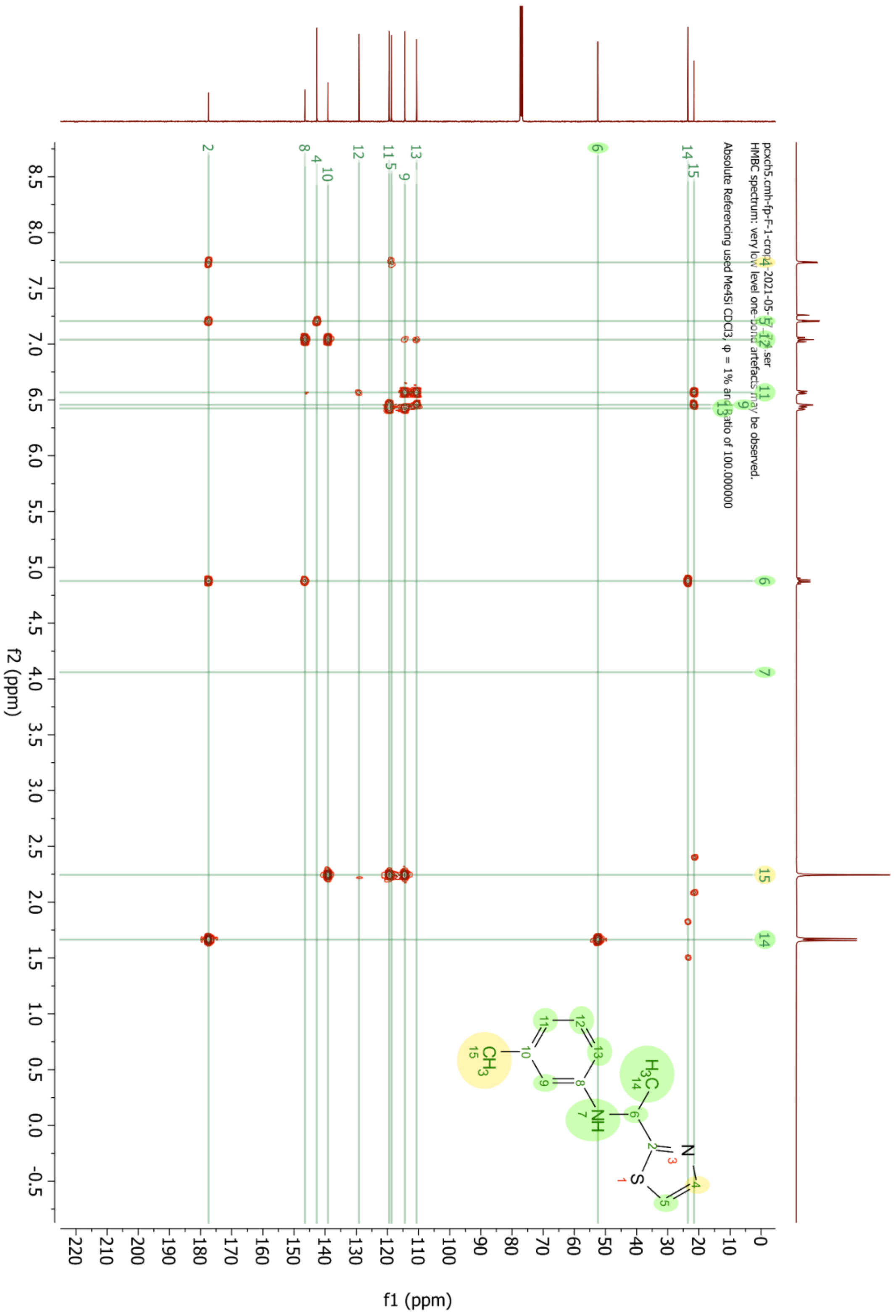

5.8 *3-(o-fluoro-α-methylbenzylamino)pyridine **6a***

5.8.1 *^{1}H-NMR*

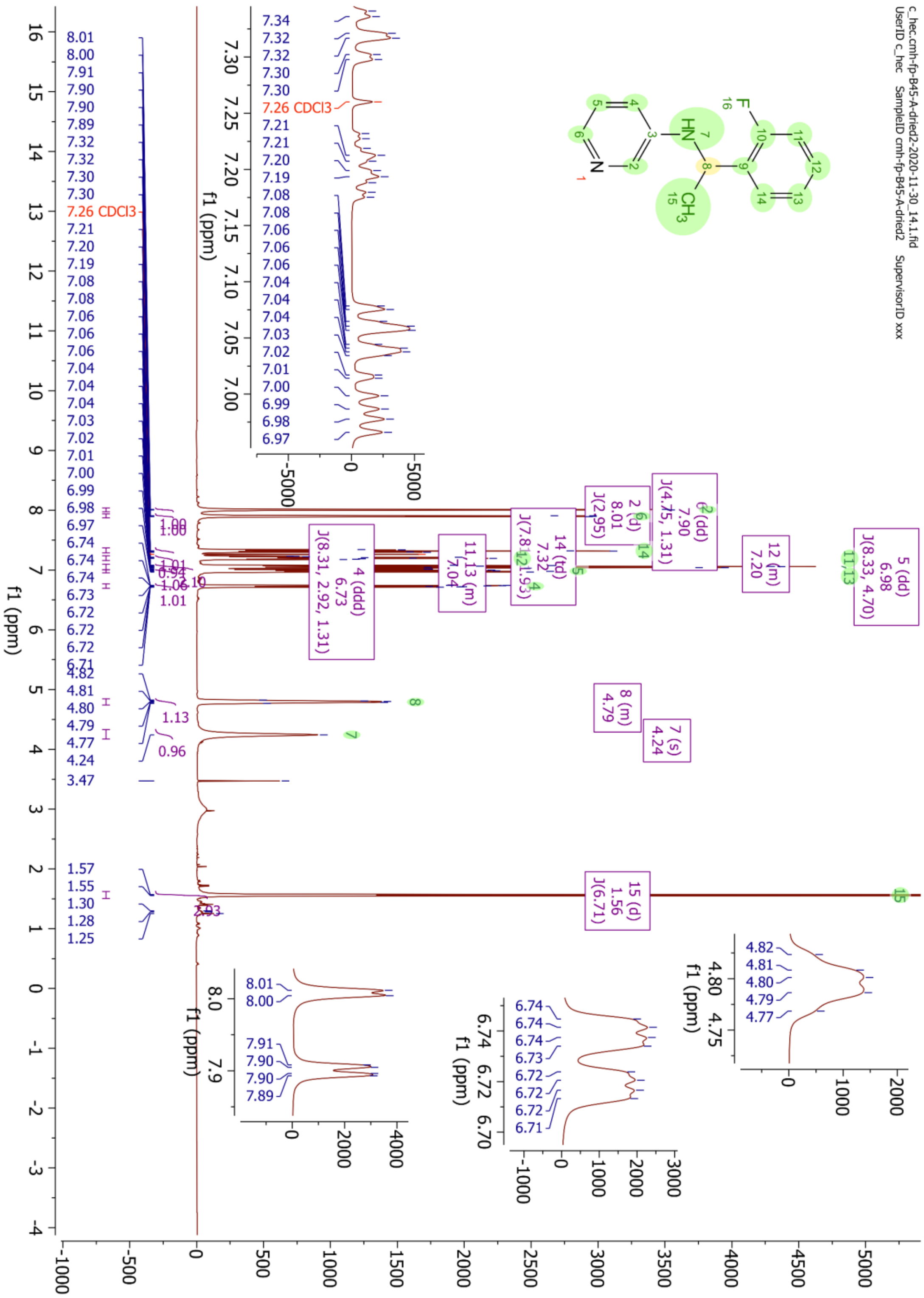

5.8.2 ¹³C-{¹H}-NMR

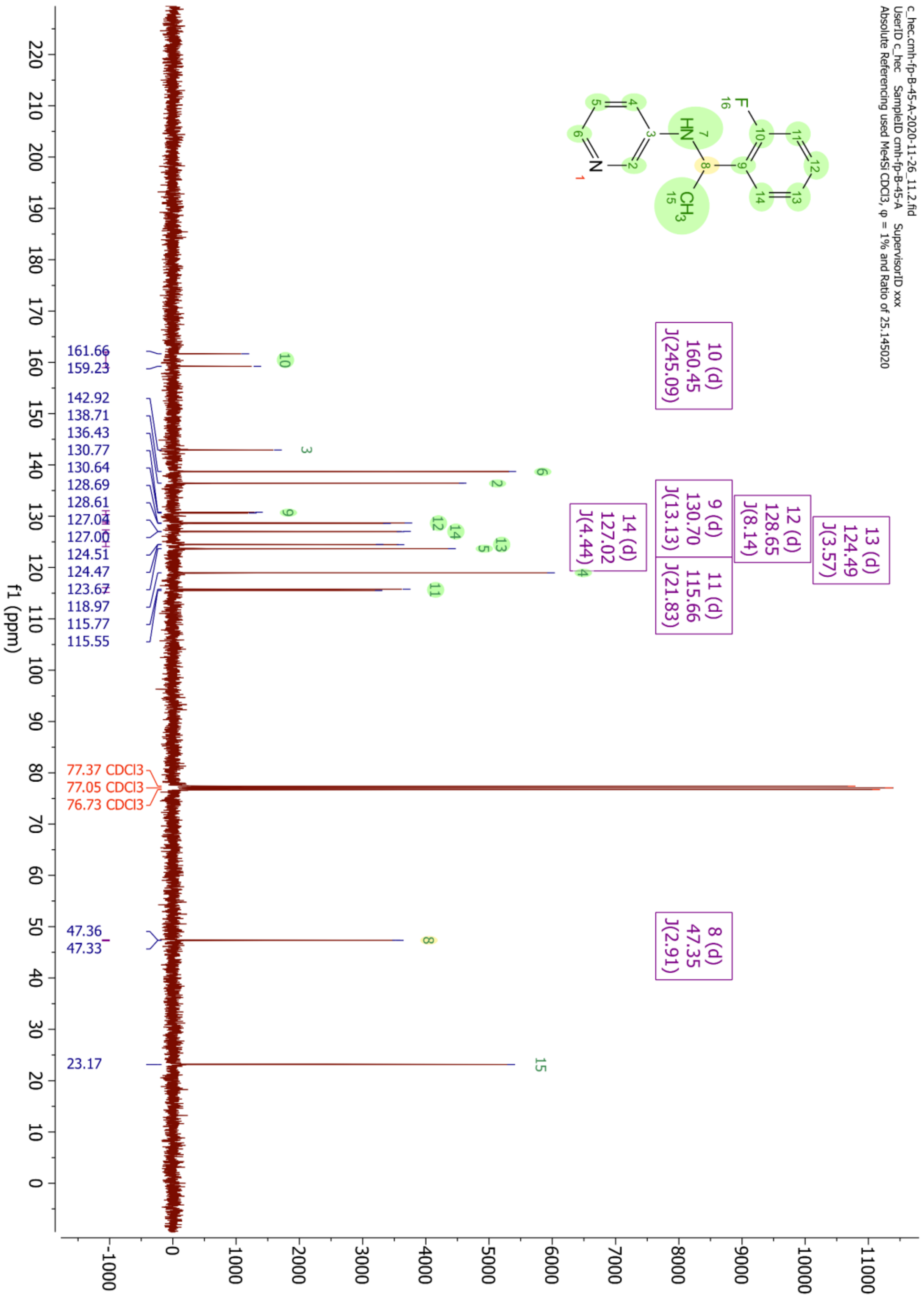

5.8.3 ^{19}F-NMR

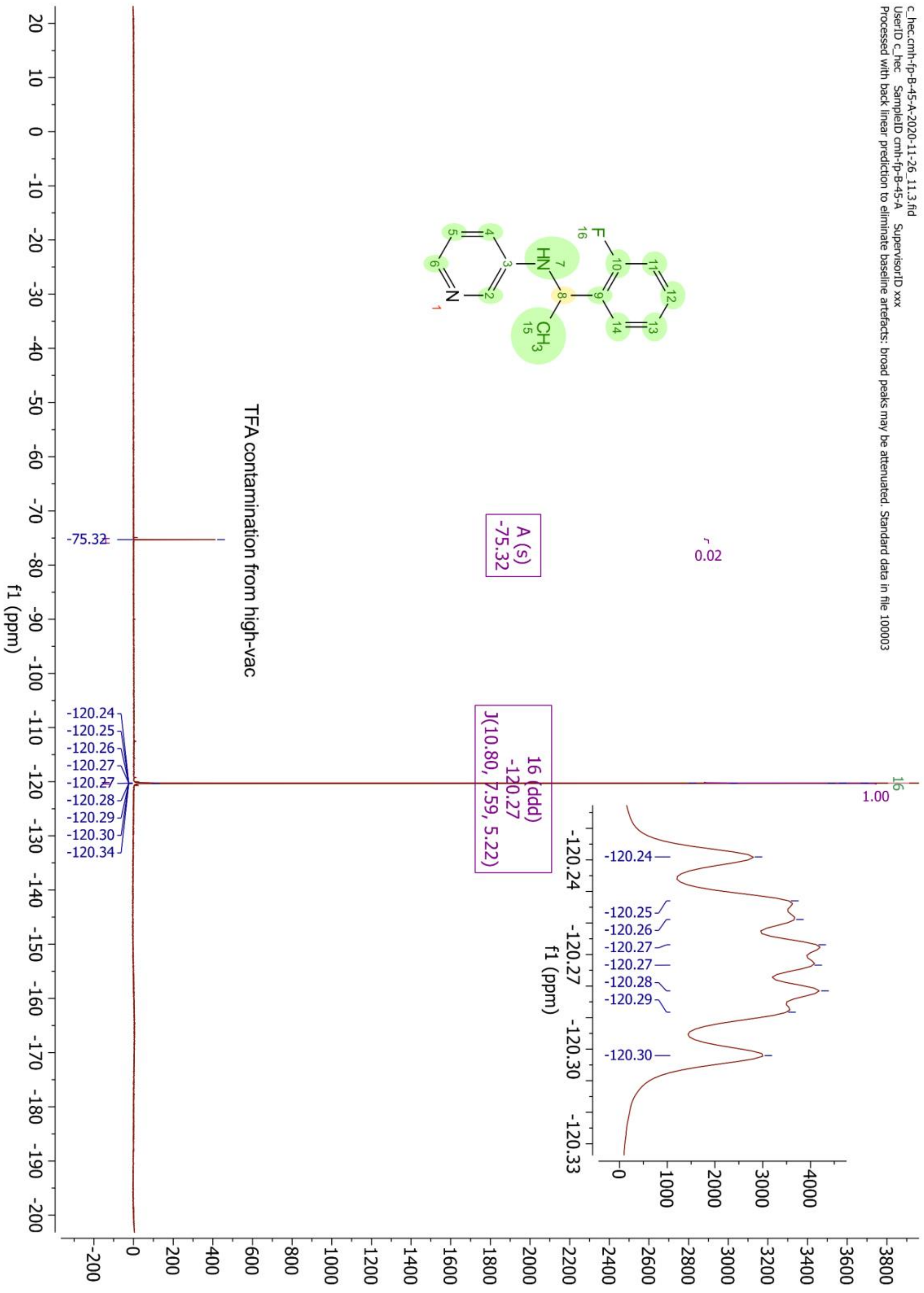
c_hec.cmh-fp-B-45-A-2020-11-26_11.3.fid
UserID c_hec SampleID cmh-fp-B-45-A SupervisorID xxx
Processed with back linear prediction to eliminate baseline artefacts: broad peaks may be attenuated. Standard data in file 100003
TFA contamination from high-vac
A (s)
-75.32
16 (ddd)
-120.27
J(10.80, 7.59, 5.22)
f1 (ppm)

5.8.4 ^{1}H-^{1}H-COSY

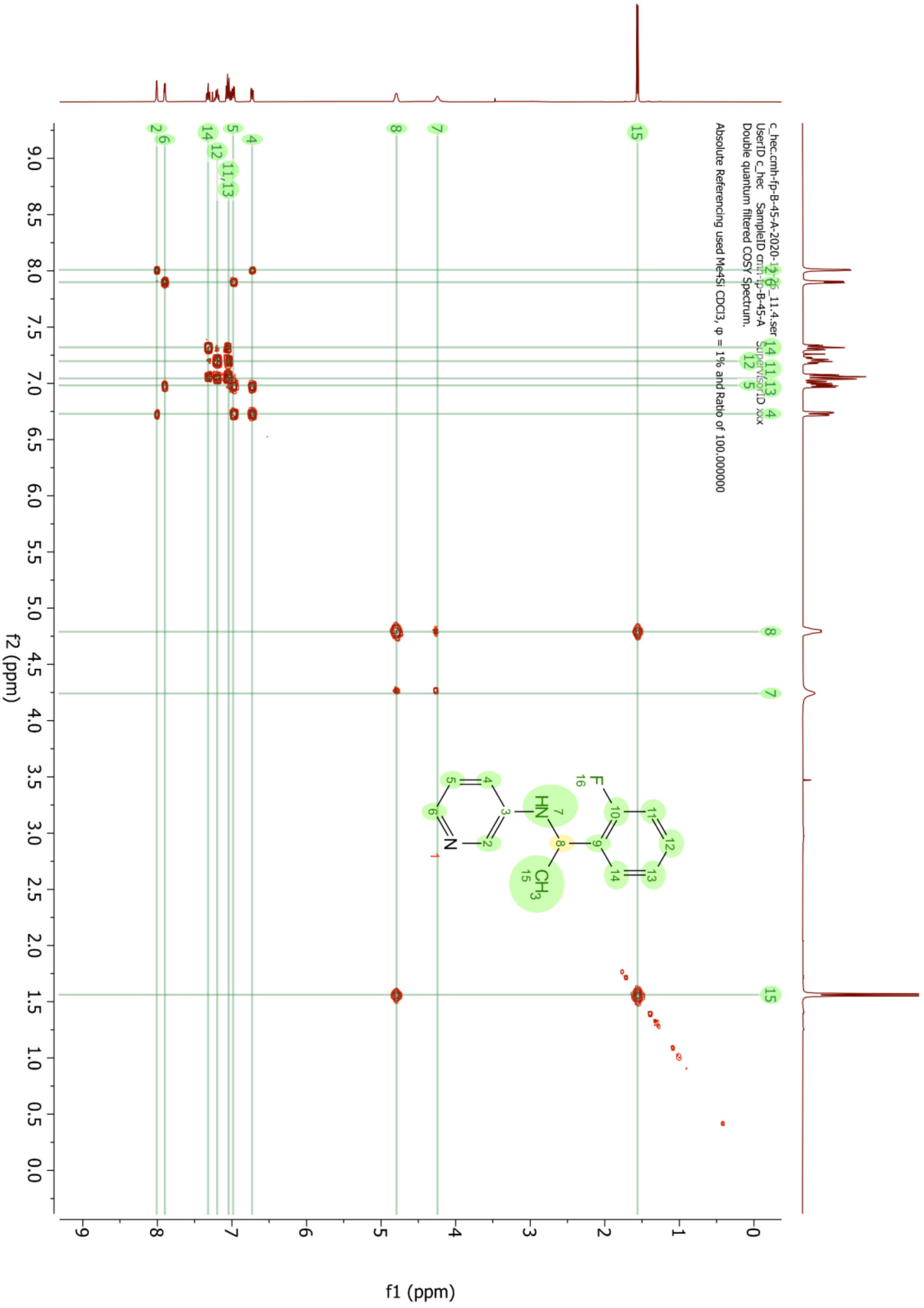

5.8.5 ^{1}H-^{13}C-HSQC-ME

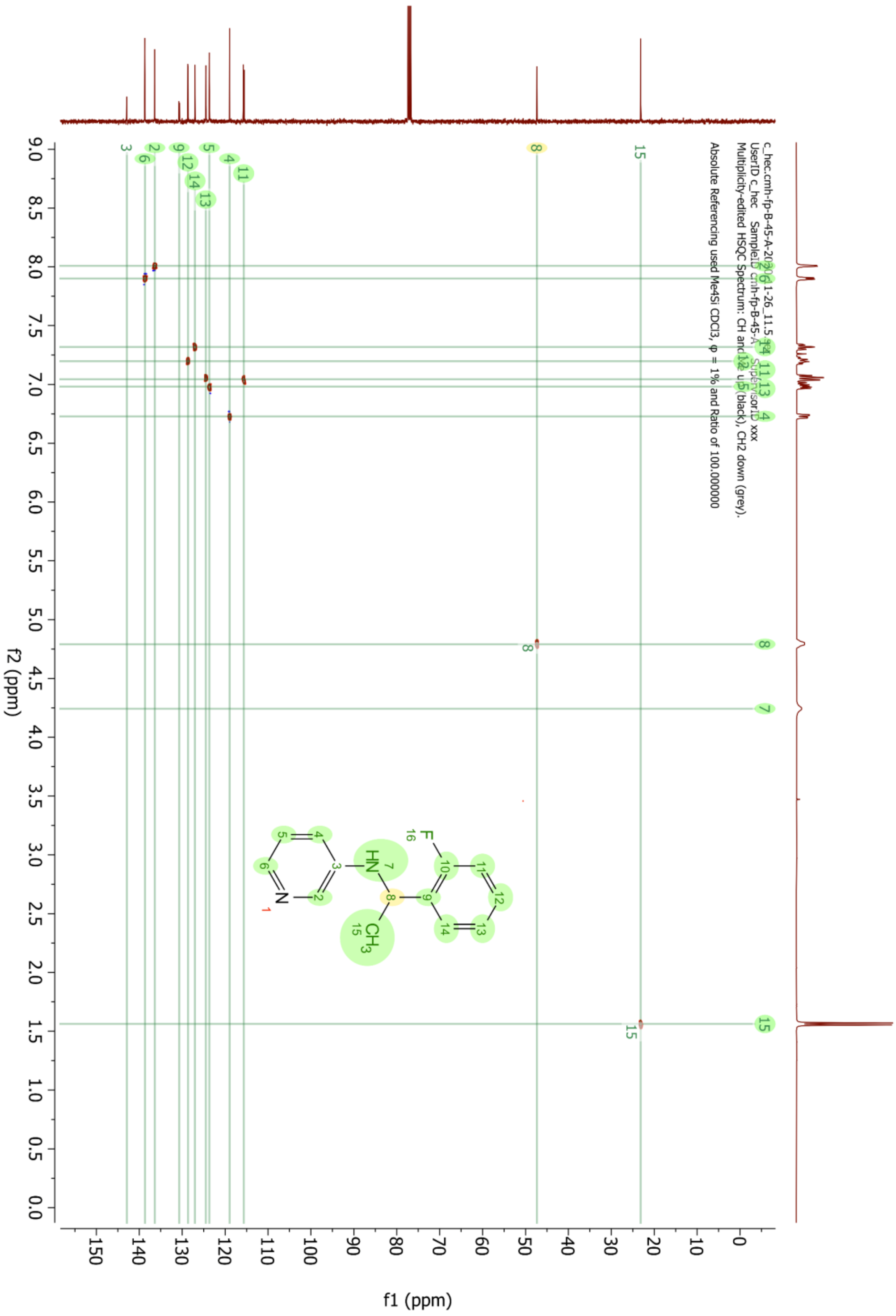

5.8.6 ^{1}H-^{13}C-HMBC

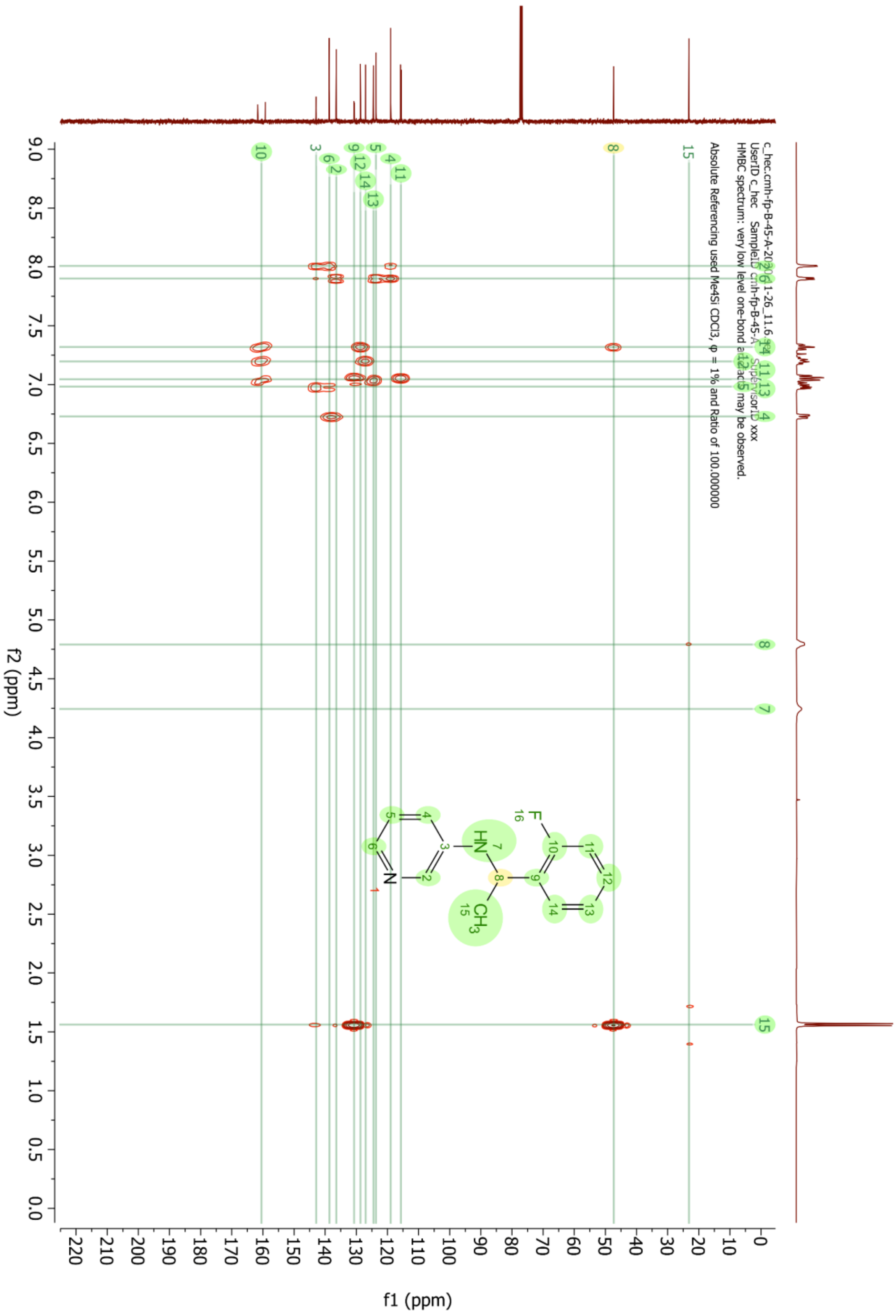

5.9 *4-(o-fluoro-α-methylbenzylamino)isoquinoline* ***6b***

5.9.1 *^{1}H-NMR*

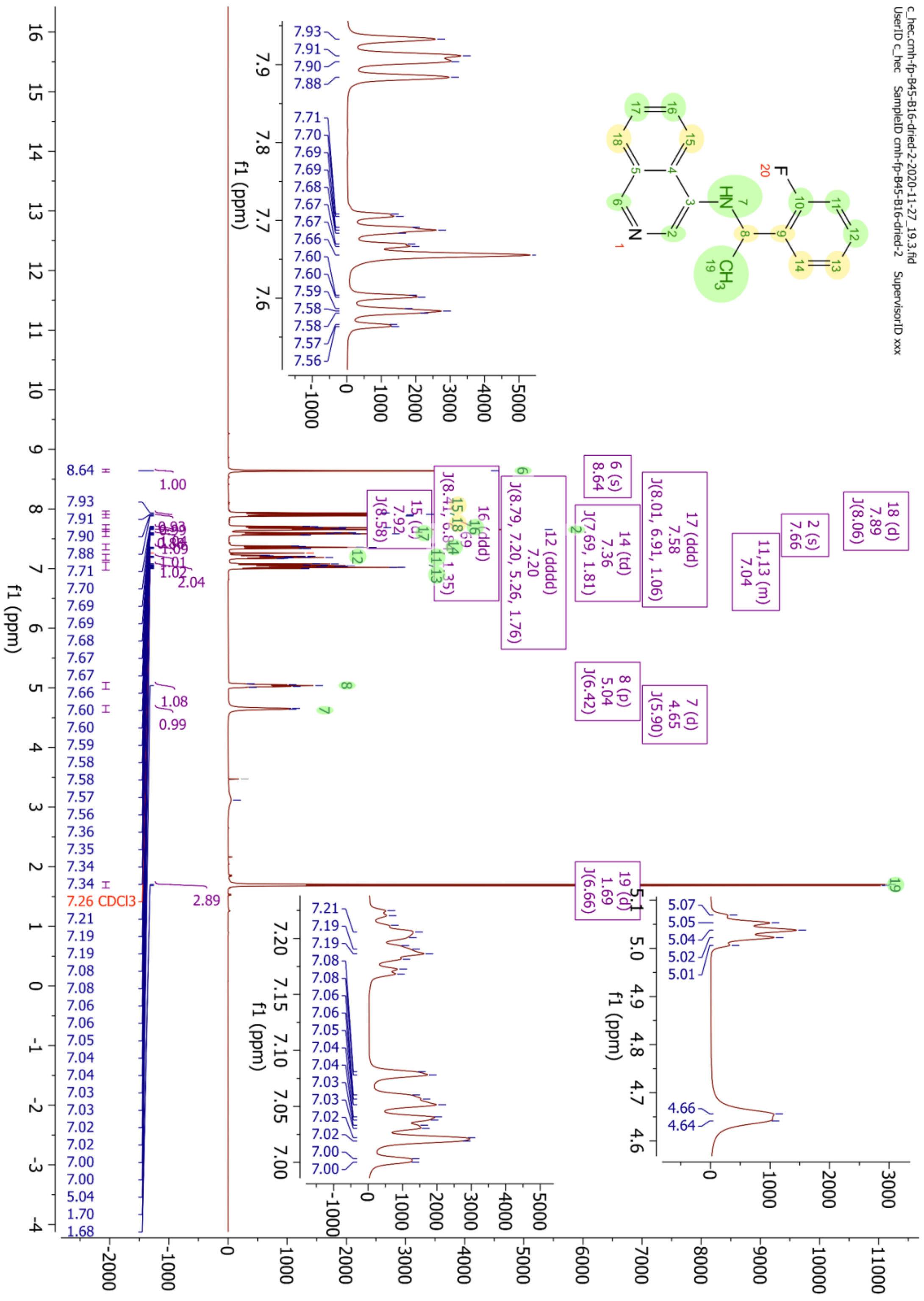

5.9.2 ^{13}C-{^{1}H}-NMR

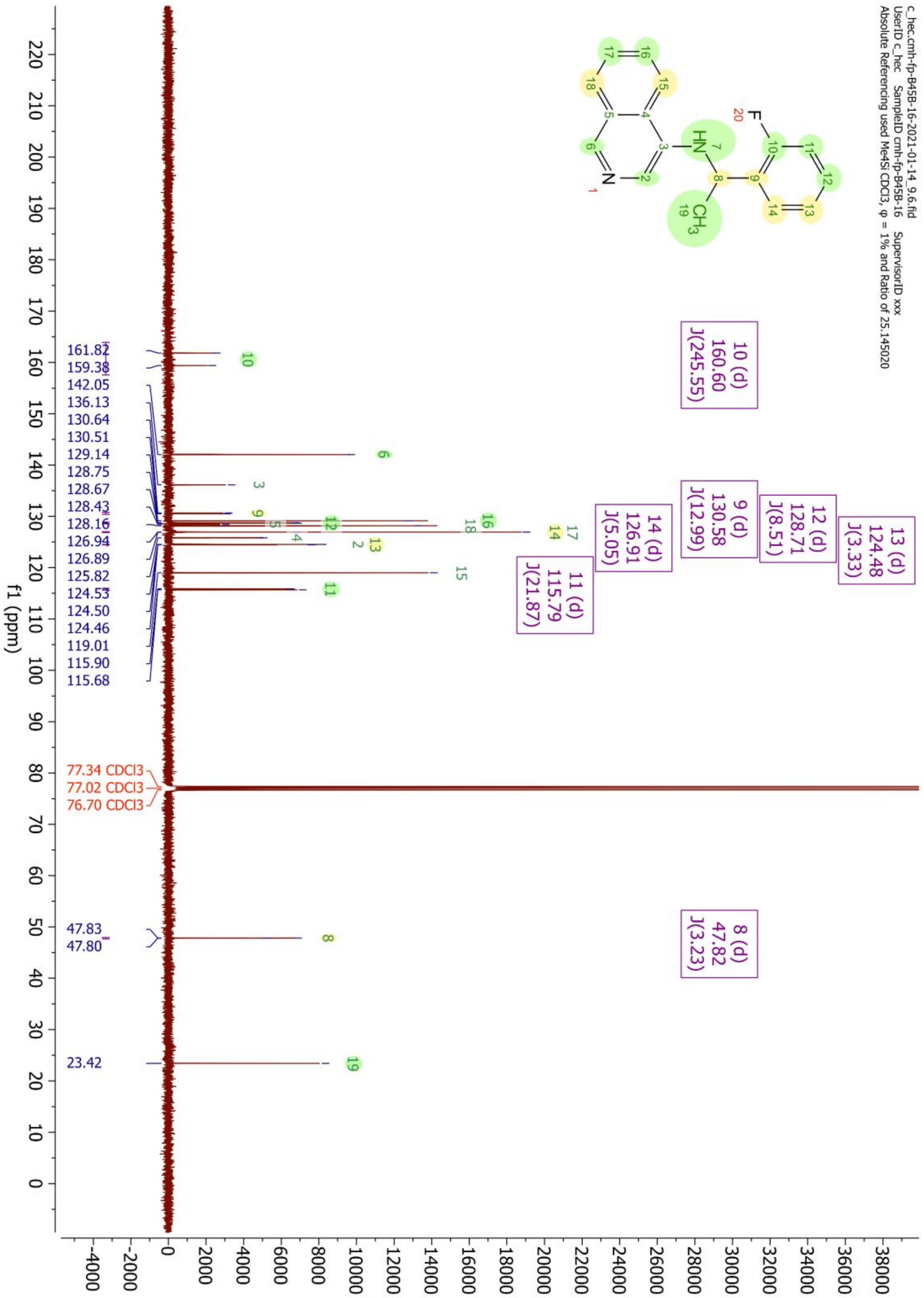

5.9.3 ^{19}F-NMR

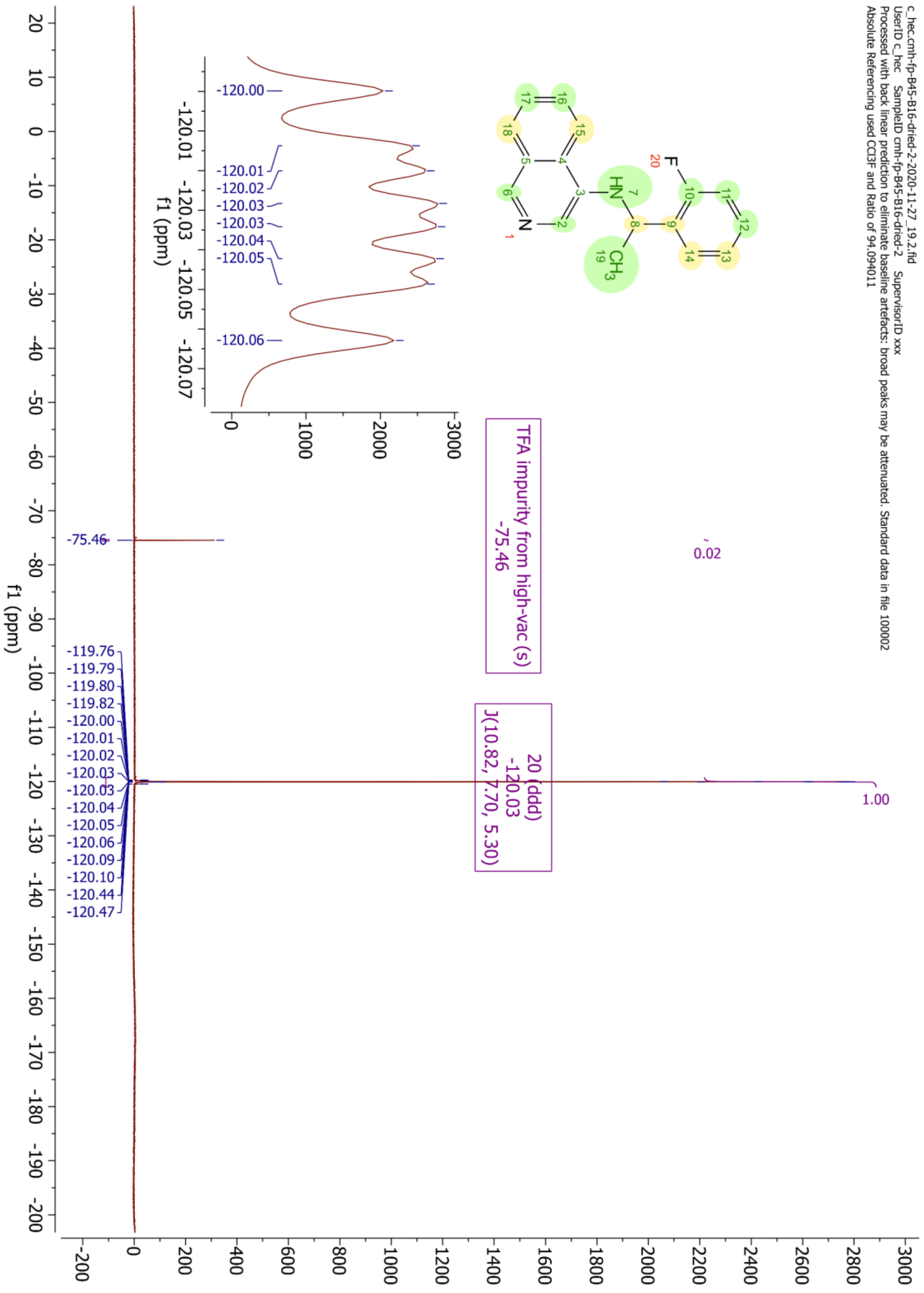

c_hec.cmh-fp-B45-B16-dried-2-2020-11-27_19.2.fid
UserID c_hec SampleID cmh-fp-B45-B16-dried-2 SupervisorID xxx
Processed with back linear prediction to eliminate baseline artefacts: broad peaks may be attenuated. Standard data in file 100002
Absolute Referencing used CCl3F and Ratio of 94.094011
TFA impurity from high-vac (s)
-75.46
20 (ddd)
-120.03
J(10.82, 7.70, 5.30)
0.02
1.00
-75.46
-119.76
-119.79
-119.80
-119.82
-120.00
-120.01
-120.02
-120.03
-120.03
-120.04
-120.05
-120.06
-120.09
-120.10
-120.44
-120.47
f1 (ppm)

5.9.4 ^{1}H-^{1}H-COSY

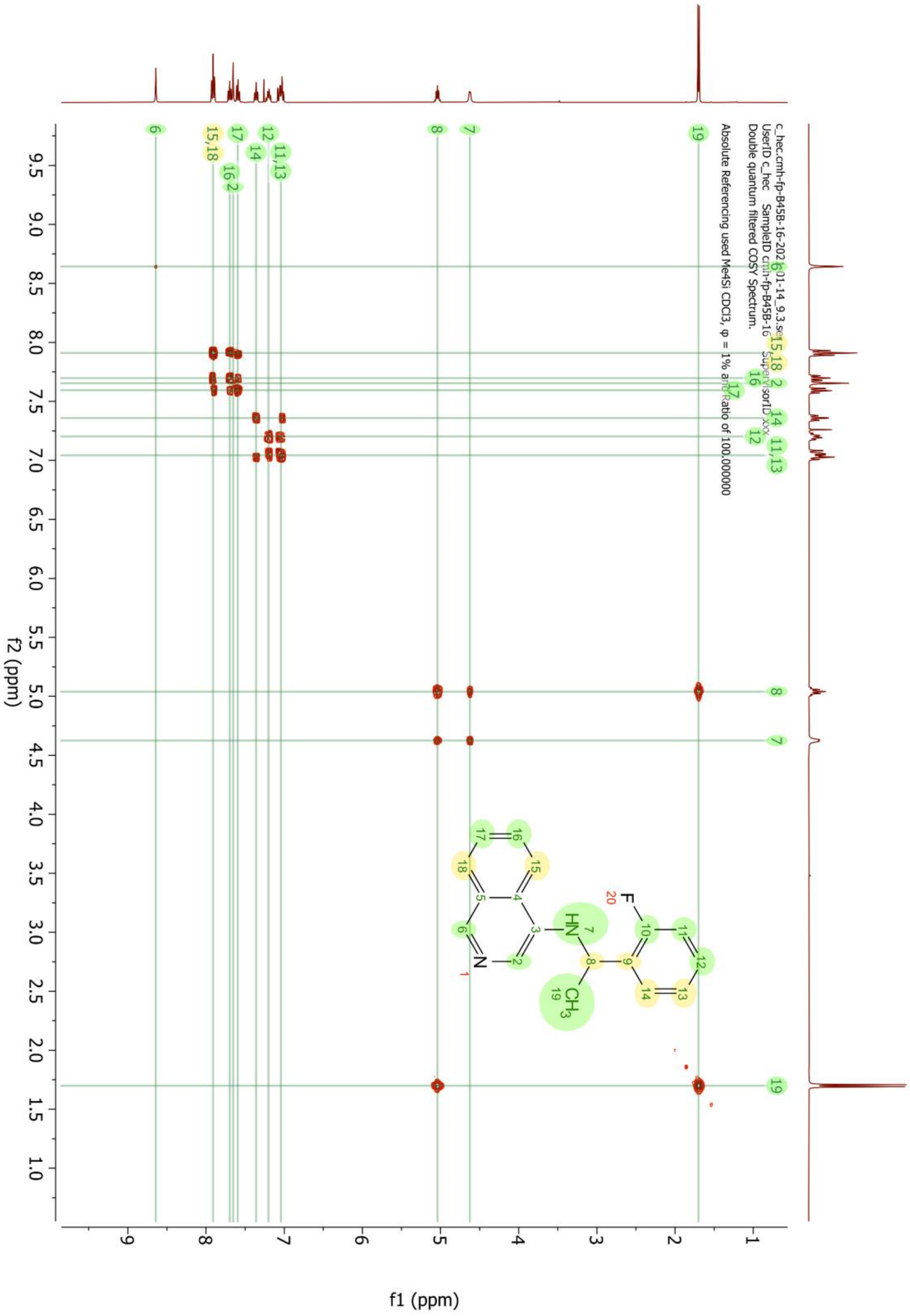

5.9.5 ^{1}H-^{13}C-HSQC-ME

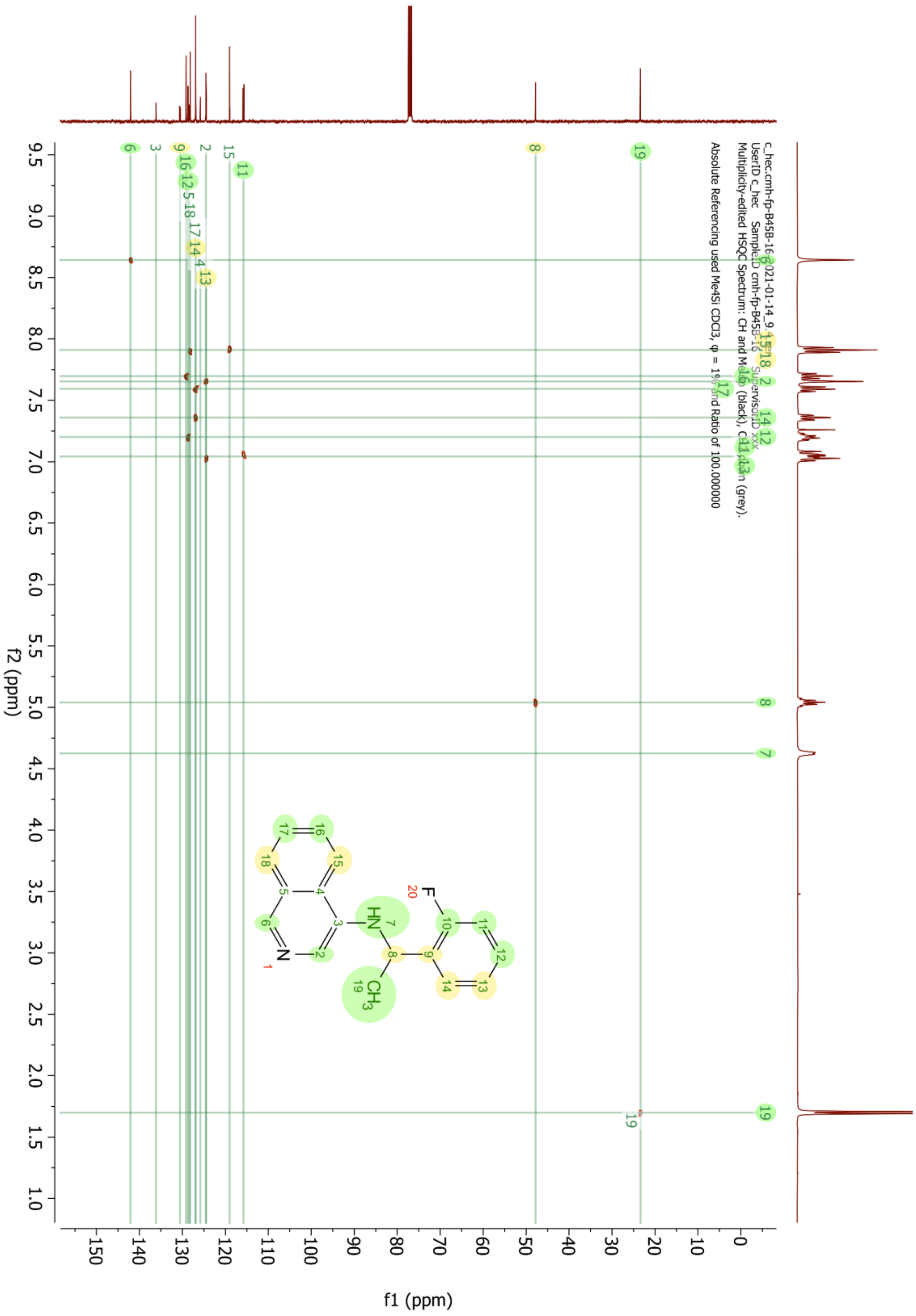

5.9.6 ¹H-¹³C-HMBC

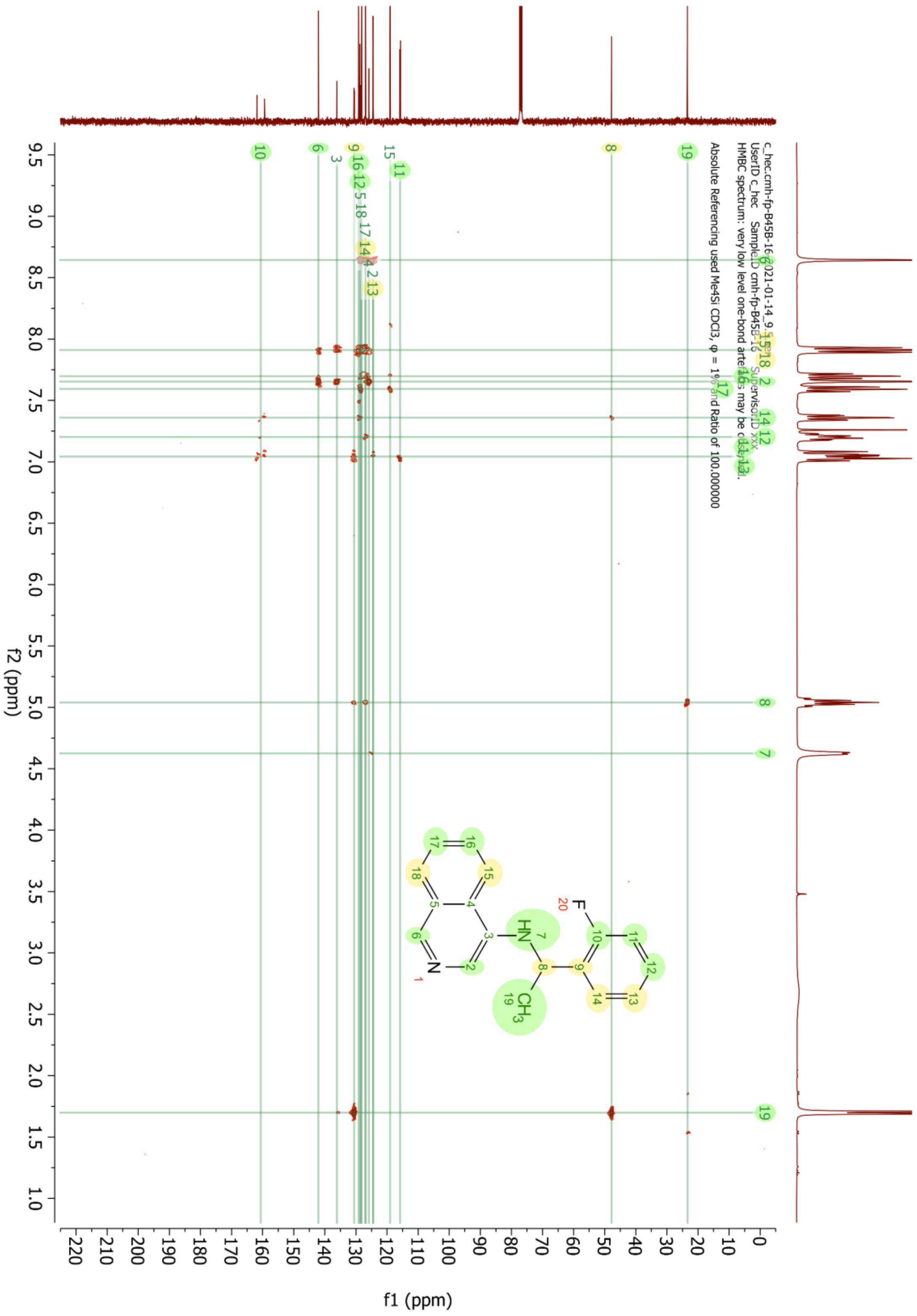

5.10 *4-(o-fluoro-α-methylbenzylamino)benzonitirile* ***6c***

5.10.1 ^{1}H-NMR

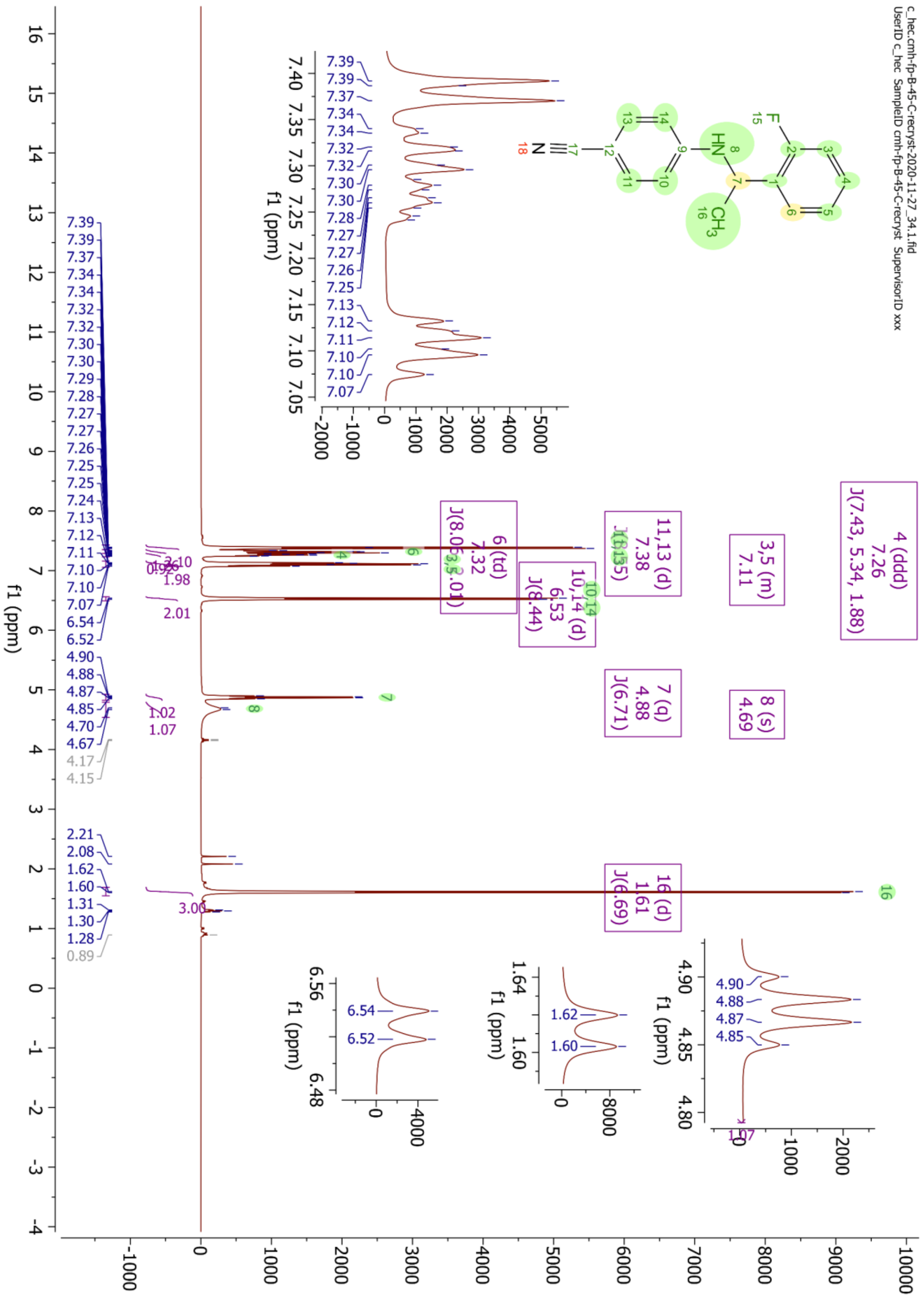

5.10.2 ^{13}C-{^{1}H}-NMR

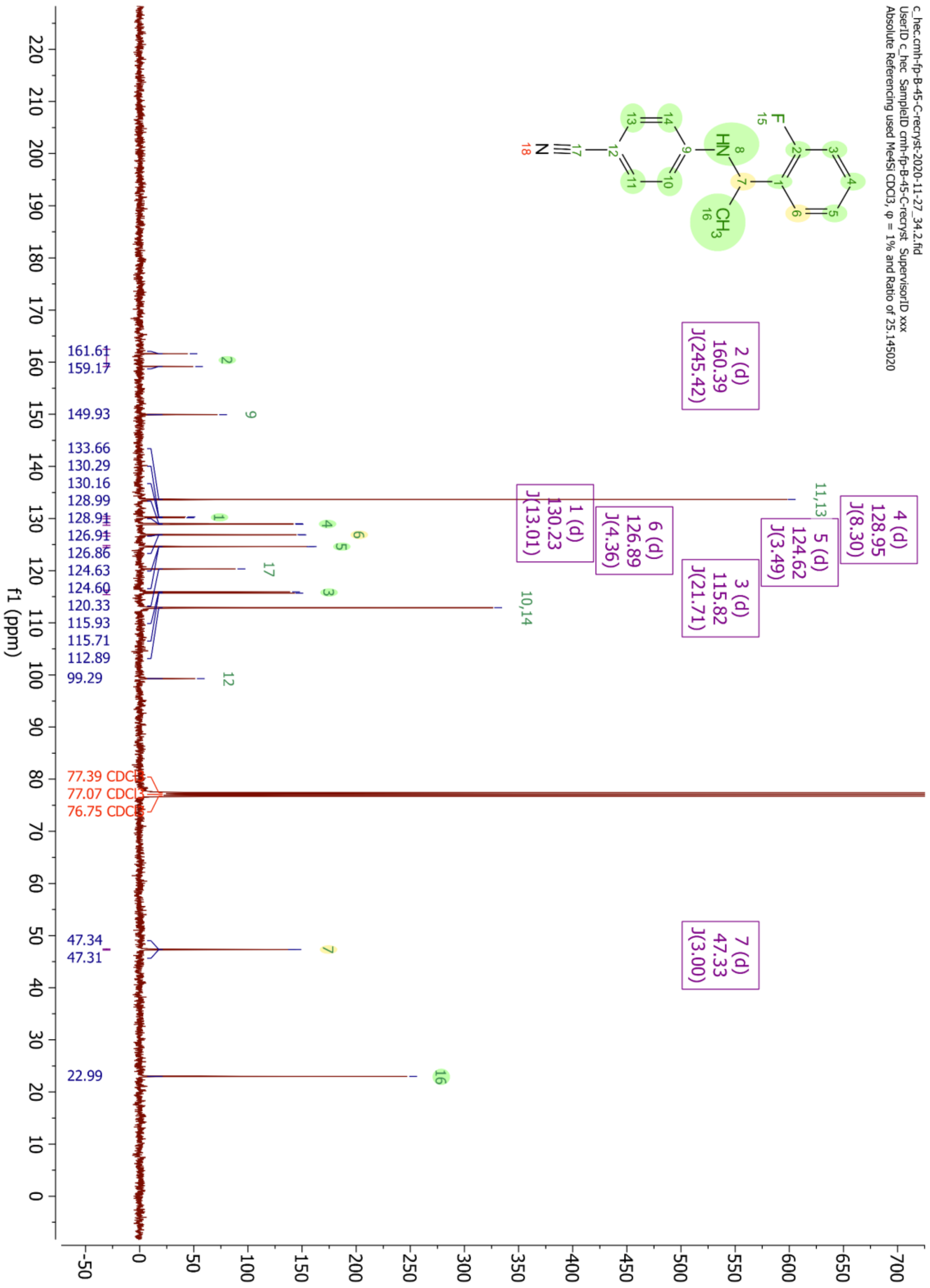

5.10.3 ^{19}F-NMR

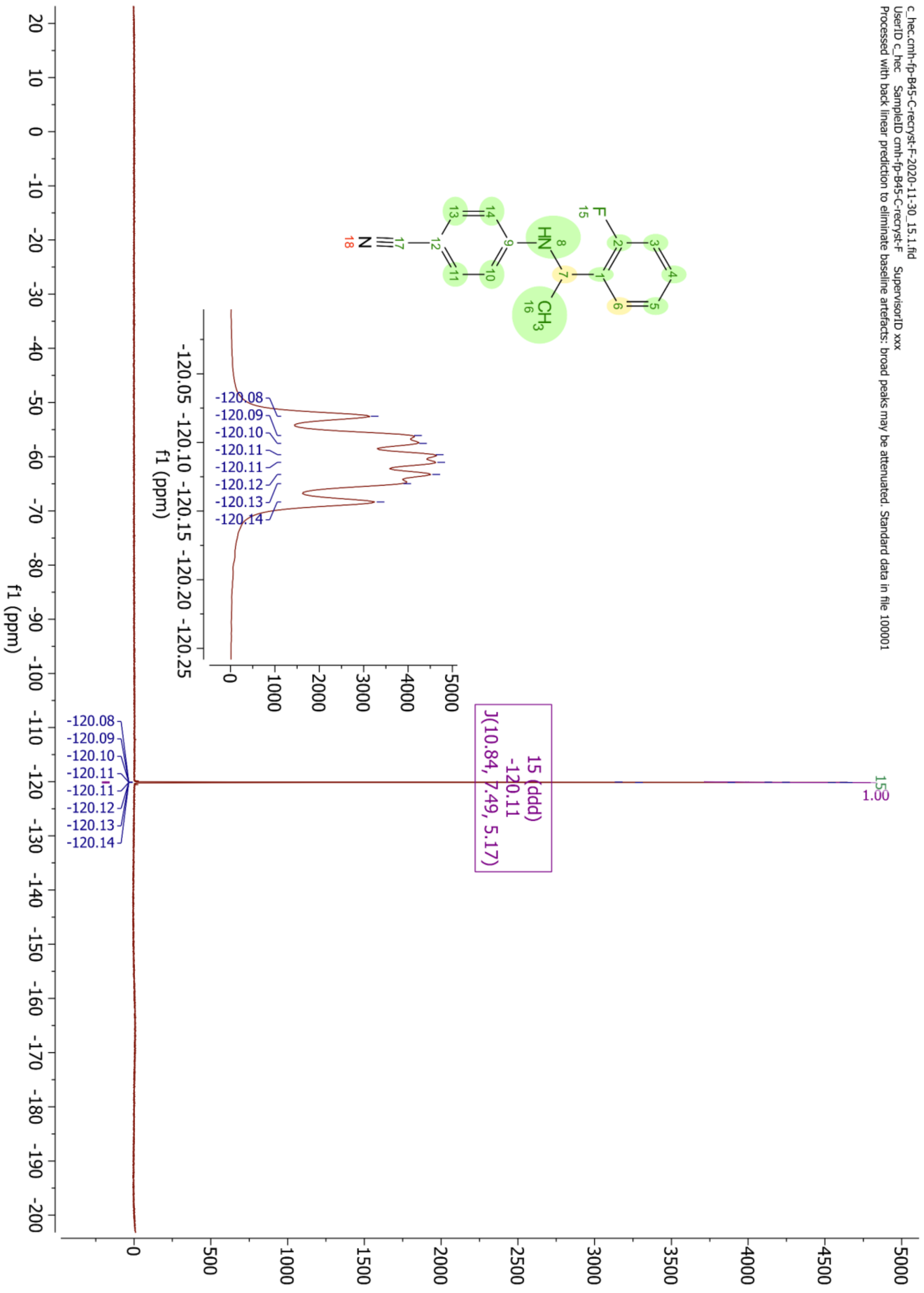
c_hec.cmh-fp-B45-C-recryst-F-2020-11-30_15.1.fid
UserID c_hec SampleID cmh-fp-B45-C-recryst-F SupervisorID xxx
Processed with back linear prediction to eliminate baseline artefacts: broad peaks may be attenuated. Standard data in file 100001
15 (ddd)
-120.11
J(10.84, 7.49, 5.17)
-120.08
-120.09
-120.10
-120.11
-120.11
-120.12
-120.13
-120.14
f1 (ppm)

5.10.4 ^{1}H-^{1}H-COSY

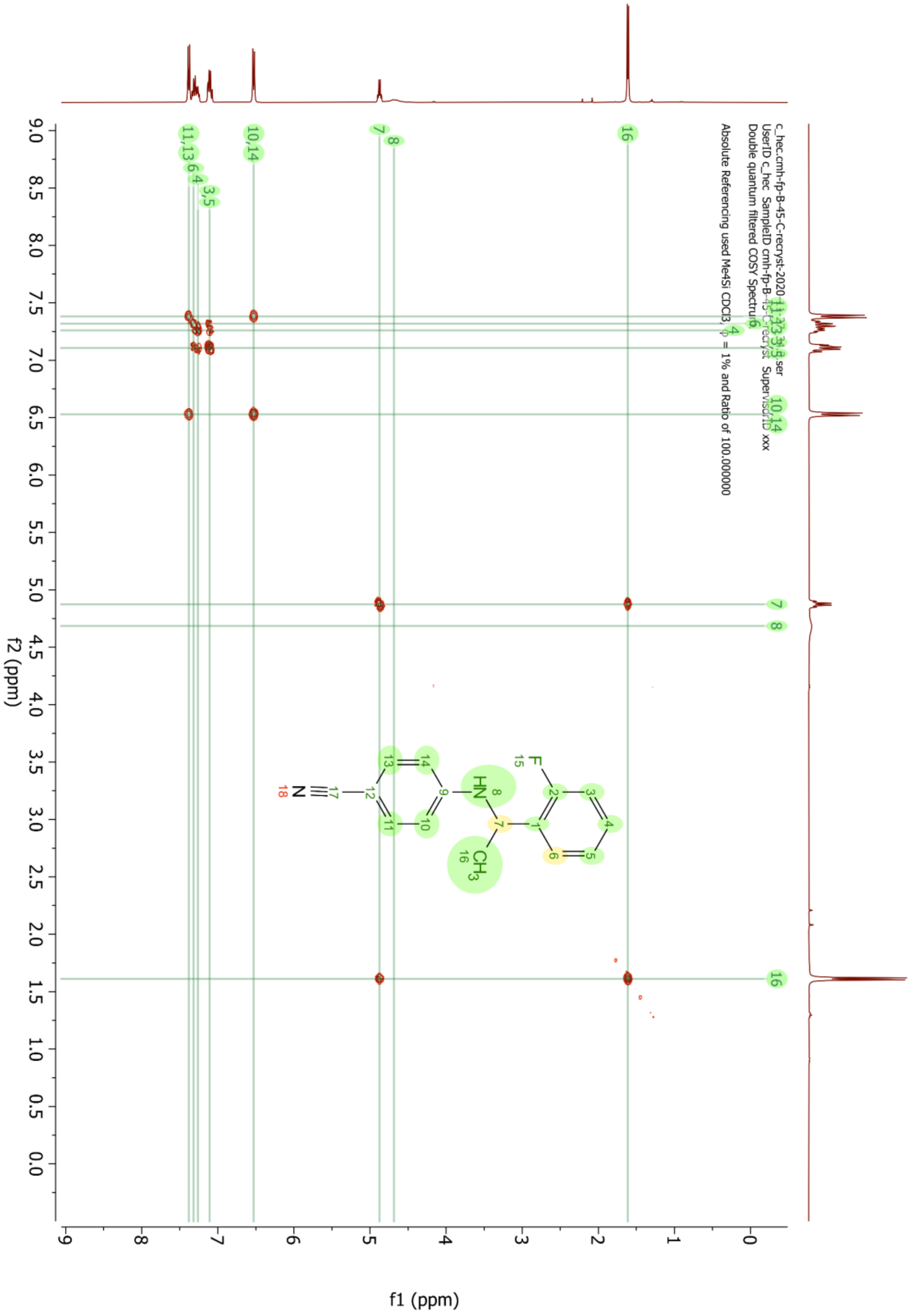

5.10.5 ^{1}H-^{13}C-HSQC-ME

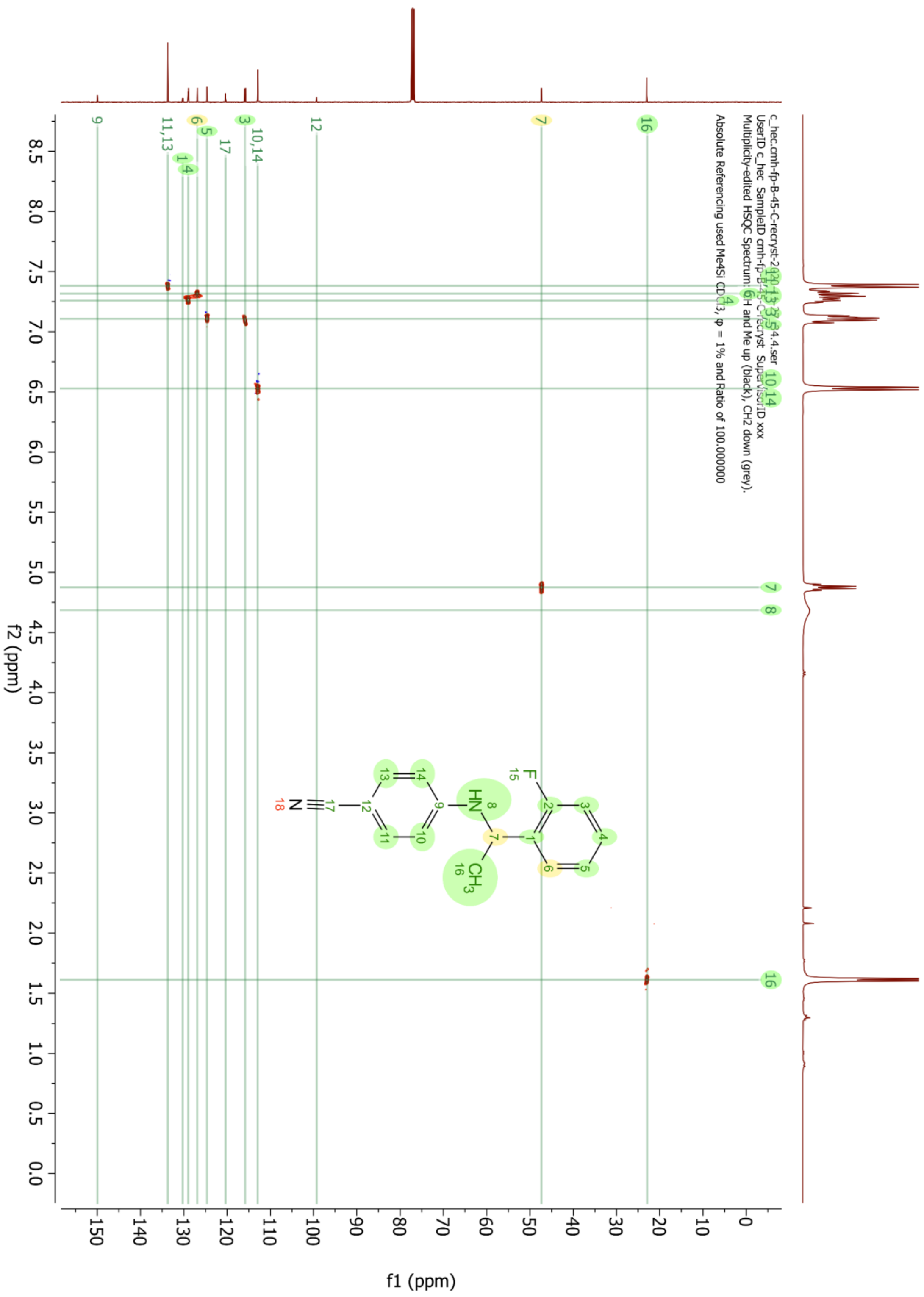

5.10.6 ^{1}H-^{13}C-HMBC

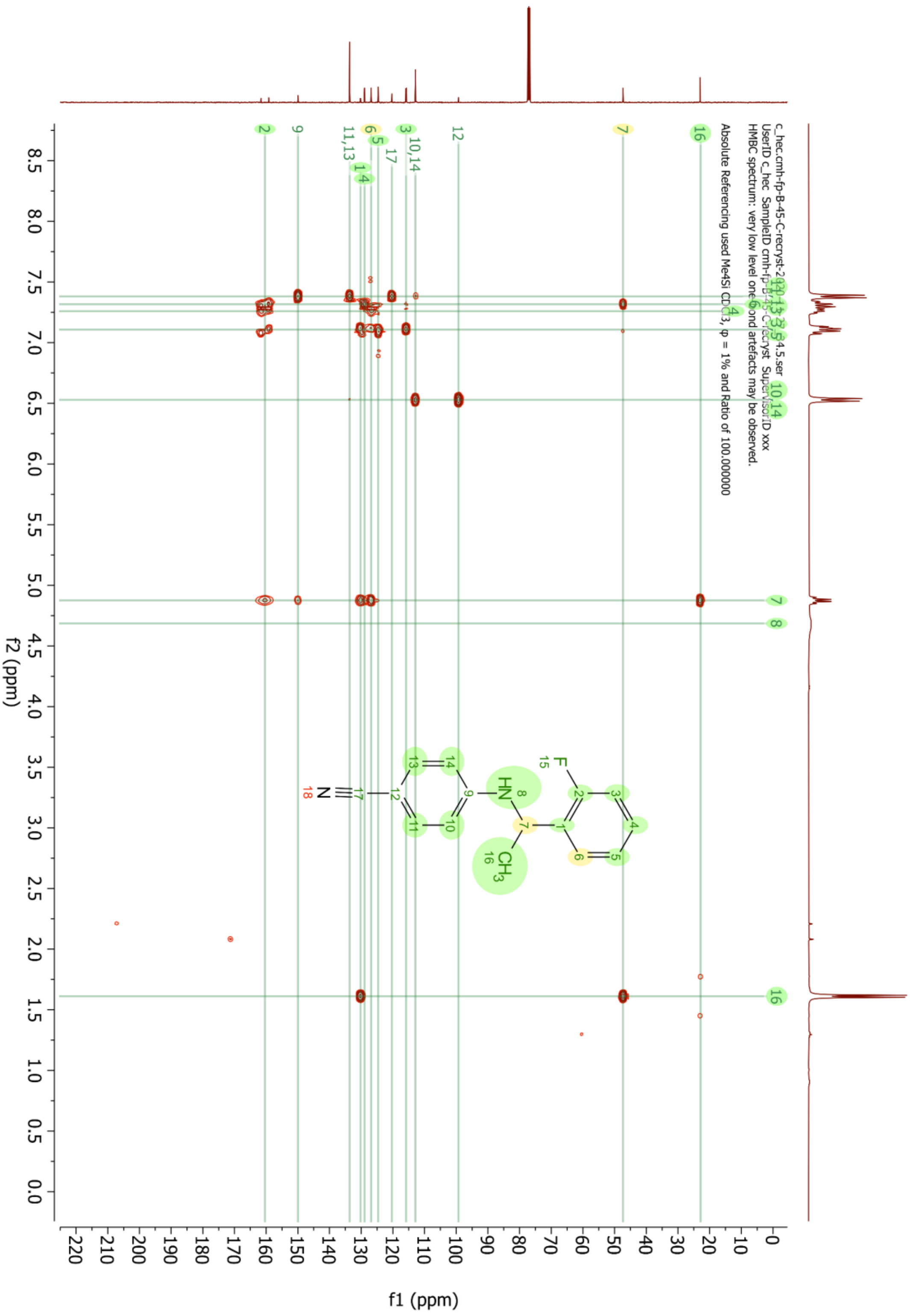

5.11 *4-(o-fluoro-α-methylbenzylamino)anisole* ***6d***

5.11.1 ^{1}H-NMR

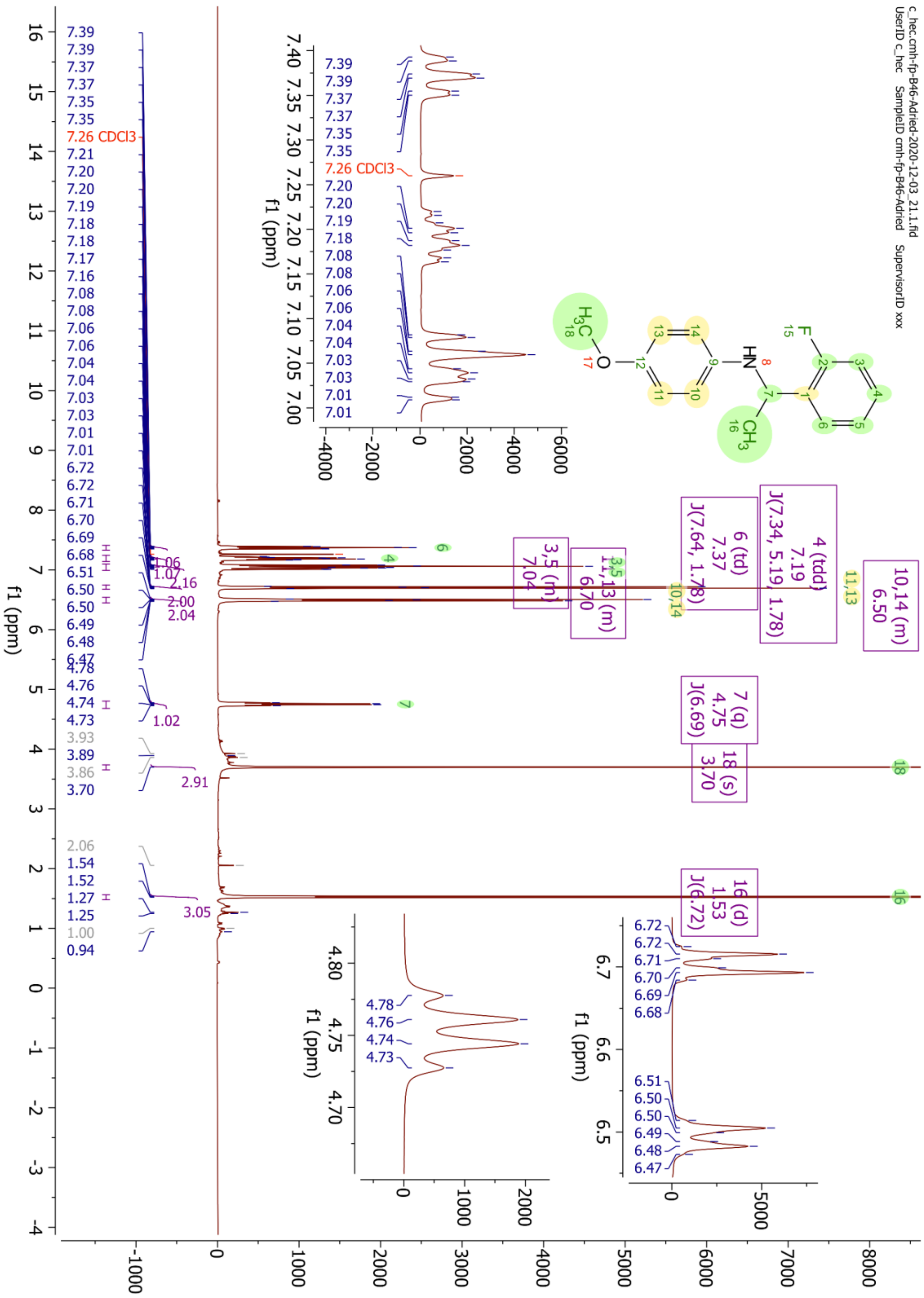

5.11.2 ^{13}C-{^{1}H}-NMR

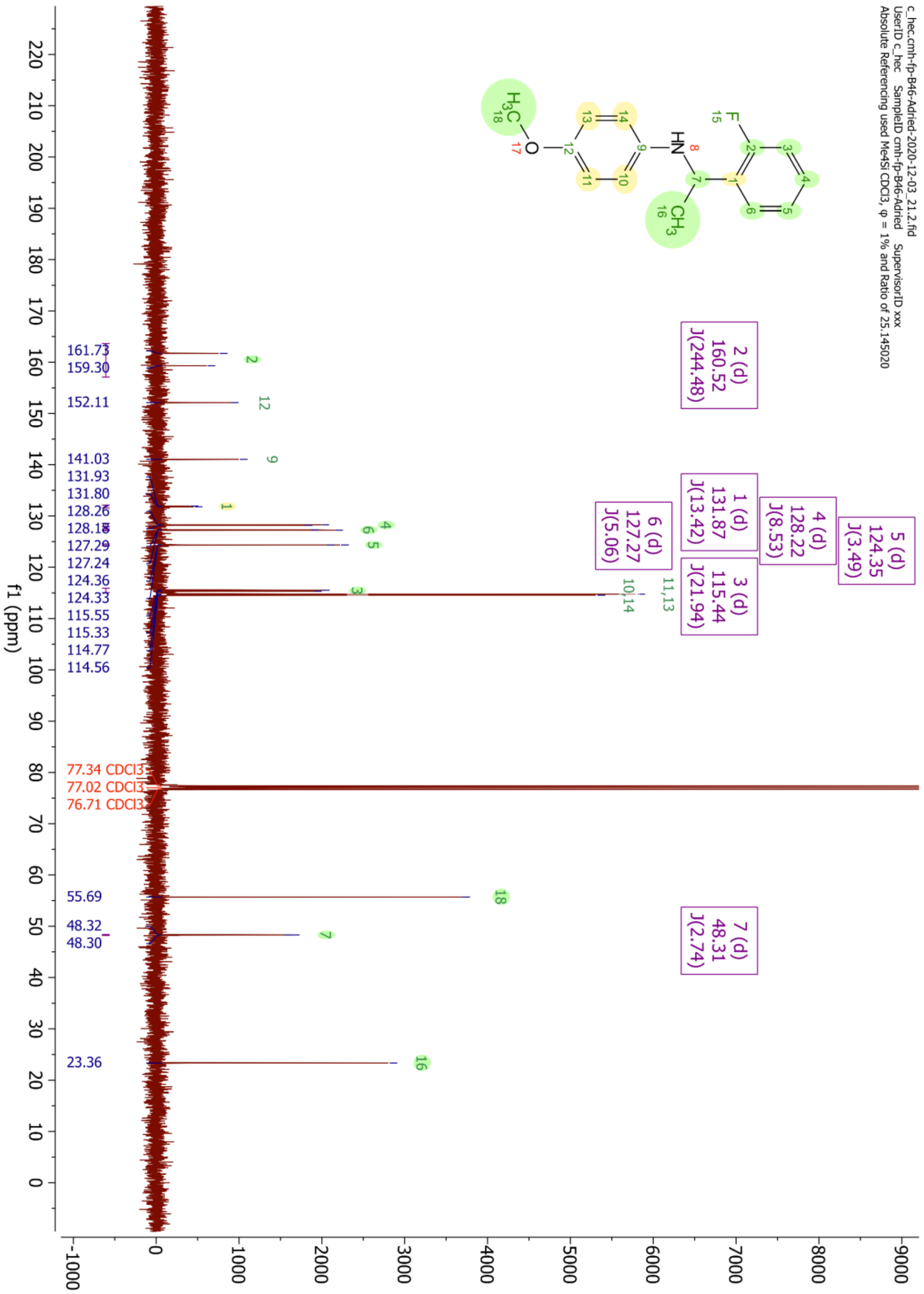

5.11.3 ^{19}F-NMR

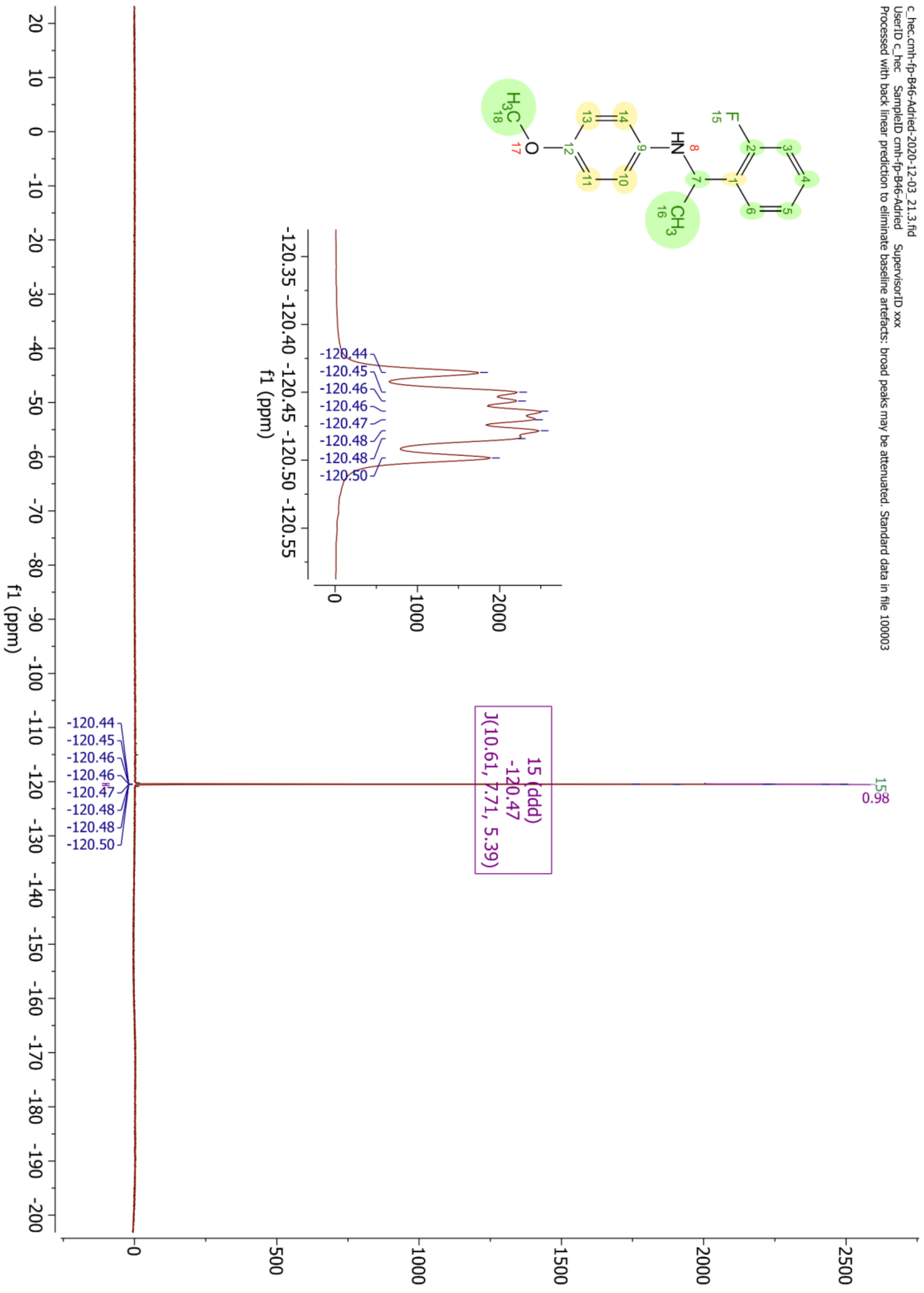
c_hec.cmh-fp-B46-Adried-2020-12-03_21.3.fid
UserID c_hec SampleID cmh-fp-B46-Adried SupervisorID xxx
Processed with back linear prediction to eliminate baseline artefacts: broad peaks may be attenuated. Standard data in file 100003
15 (ddd)
-120.47
J(10.61, 7.71, 5.39)
-120.44
-120.45
-120.46
-120.46
-120.47
-120.48
-120.48
-120.50
f1 (ppm)
0.98

5.11.4 ^{1}H-^{1}H-COSY

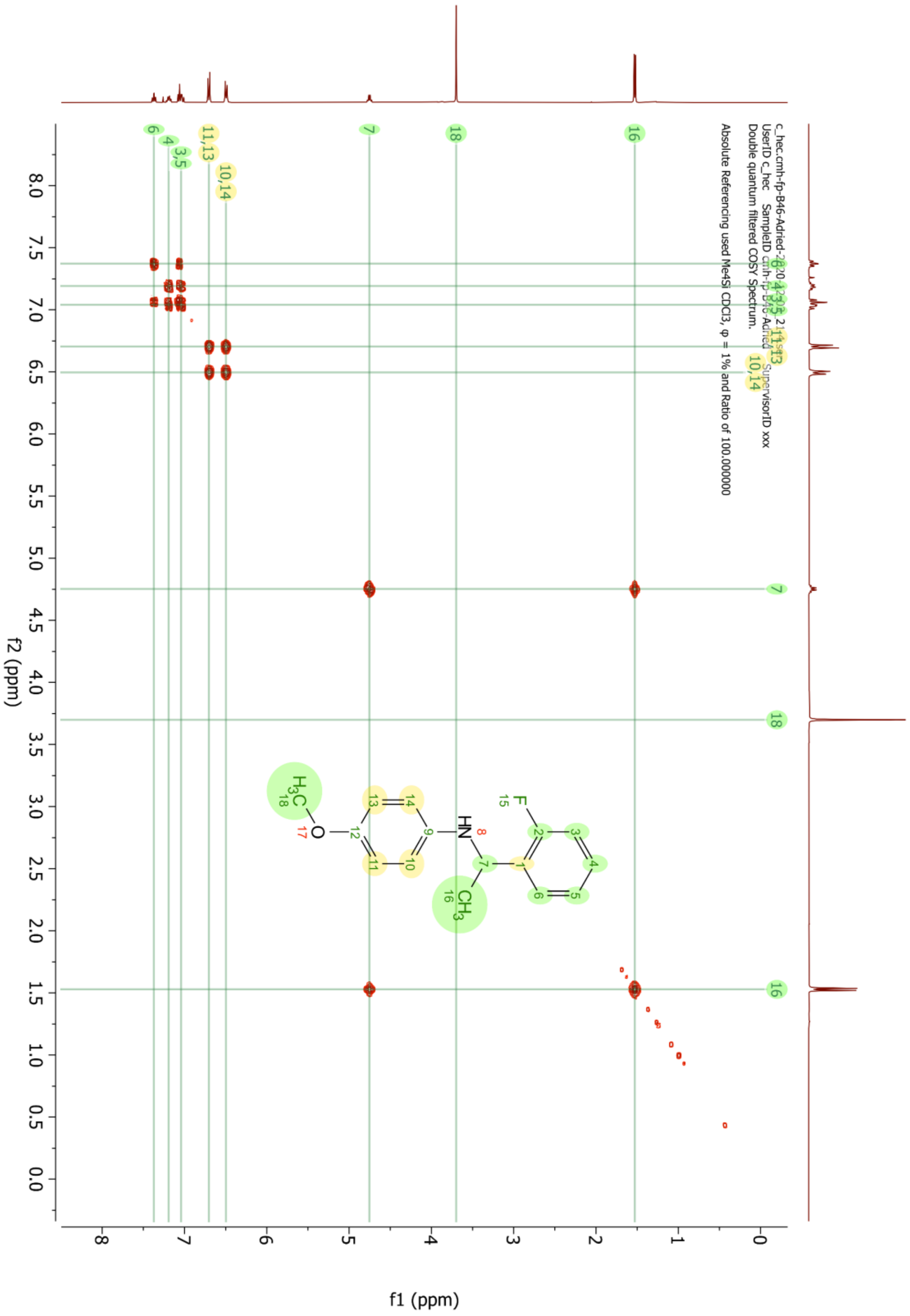

5.11.5 ¹H-¹³C-HSQC-ME

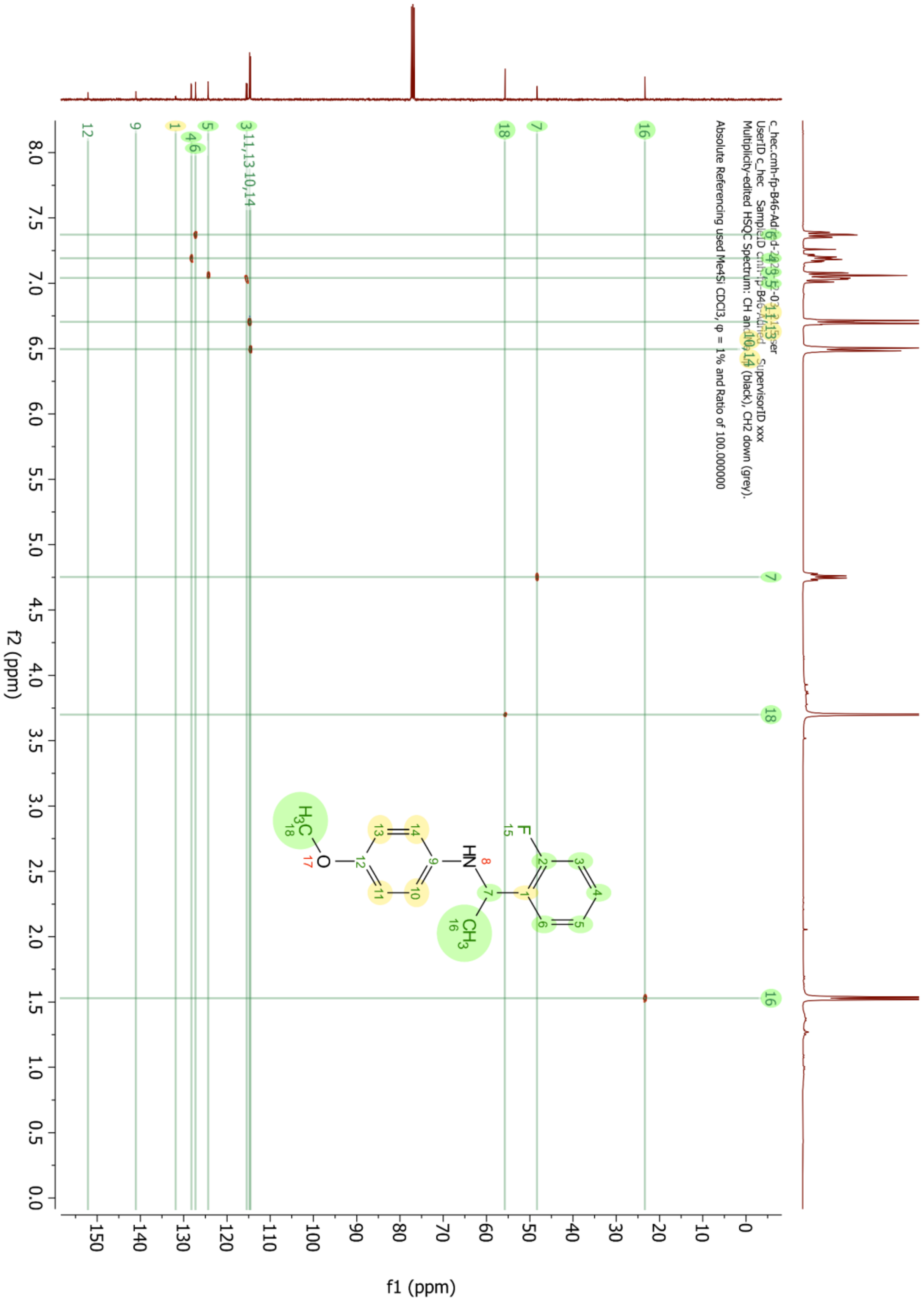

5.11.6 ¹H-¹³C-HMBC

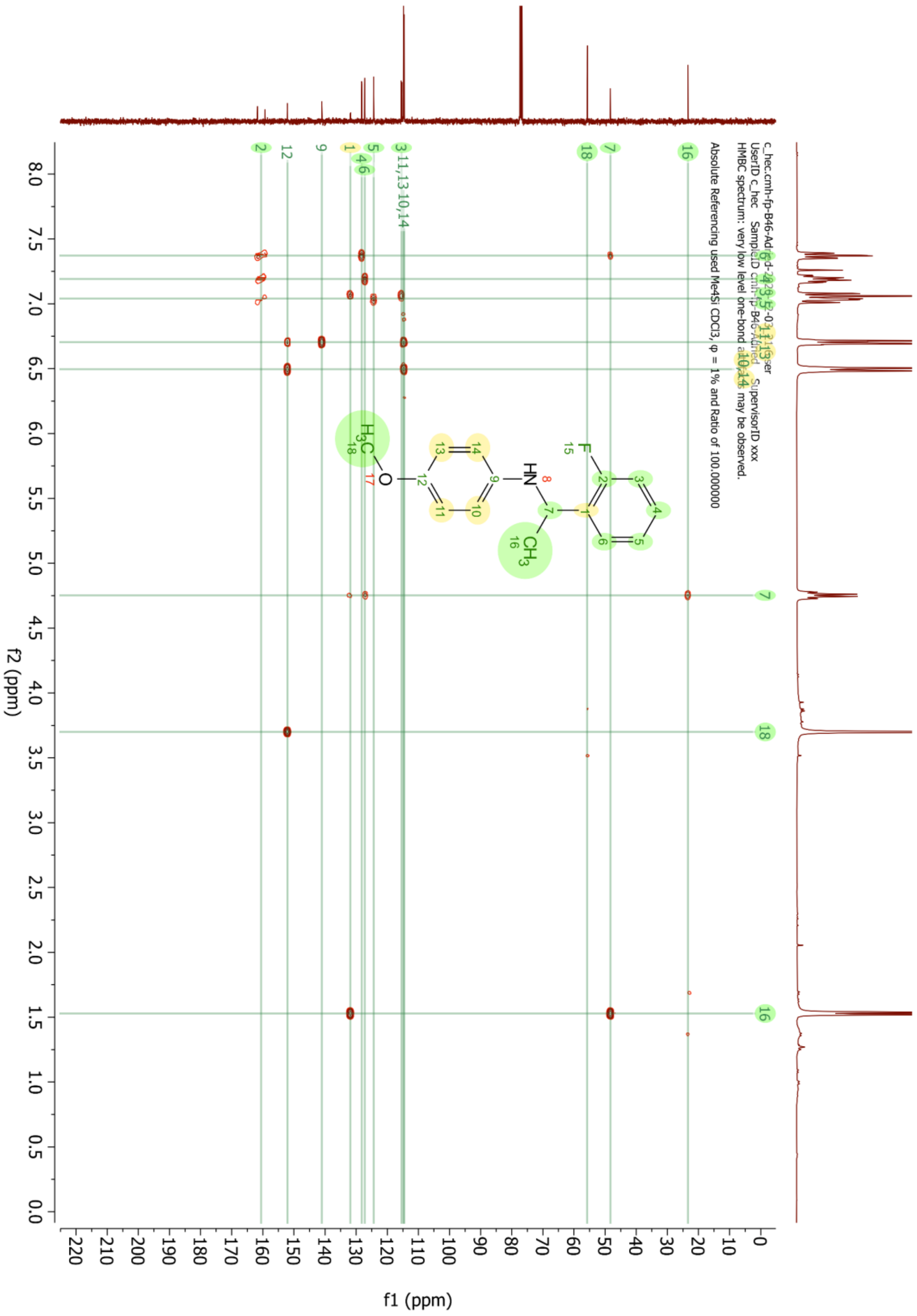

6 Supporting References

[1] C. M. Heckmann, L. J. Gourlay, B. Dominguez, F. Paradisi, *Front. Bioeng. Biotechnol.* **2020**, *8*,

707.

[2] F. Dall'Oglio, M. L. Contente, P. Conti, F. Molinari, D. Monfredi, A. Pinto, D. Romano, D. Ubiali, L. Tamborini, I. Serra, *Catal. Commun.* **2017**, *93*, 29–32.

[3] L. Cerioli, M. Planchestainer, J. Cassidy, D. Tessaro, F. Paradisi, *J. Mol. Catal. B: Enzym.* **2015**, *120*, 141–150.

[4] F. W. Studier, *Protein Expr. Purif.* **2005**, *41*, 207–234.

[5] S. Schätzle, M. Höhne, E. Redestad, K. Robins, U. T. Bornscheuer, *Anal. Chem.* **2009**, *81*, 8244–8248.
